# Recent Trends in Electrochemical Sensors for Vital Biomedical Markers Using Hybrid Nanostructured Materials

**DOI:** 10.1002/advs.201902980

**Published:** 2020-05-12

**Authors:** K. Koteshwara Reddy, Harshad Bandal, Moru Satyanarayana, Kotagiri Yugender Goud, Kauveri Vengatajalabathy Gobi, Tippabattini Jayaramudu, John Amalraj, Hern Kim

**Affiliations:** ^1^ Smart Living Innovation Technology Centre Department of Energy Science and Technology Myongji University Yongin Gyeonggi‐do 17058 Republic of Korea; ^2^ Laboratory of Materials Science Instituto de Química de Recursos Naturales Universidad de Talca P.O. Box 747 Talca 3460000 Chile; ^3^ Department of Chemistry National Institute of Technology Warangal Warangal Telangana 506004 India

**Keywords:** biomarkers, electrochemical biosensors, hybrid nanocomposites, hybrid nanostructures, recognition elements

## Abstract

This work provides a succinct insight into the recent developments in electrochemical quantification of vital biomedical markers using hybrid metallic composite nanostructures. After a brief introduction to the biomarkers, five types of crucial biomarkers, which require timely and periodical monitoring, are shortlisted, namely, cancer, cardiac, inflammatory, diabetic and renal biomarkers. This review emphasizes the usage and advantages of hybrid nanostructured materials as the recognition matrices toward the detection of vital biomarkers. Different transduction methods (fluorescence, electrophoresis, chemiluminescence, electrochemiluminescence, surface plasmon resonance, surface‐enhanced Raman spectroscopy) reported for the biomarkers are discussed comprehensively to present an overview of the current research works. Recent advancements in the electrochemical (amperometric, voltammetric, and impedimetric) sensor systems constructed with metal nanoparticle‐derived hybrid composite nanostructures toward the selective detection of chosen vital biomarkers are specifically analyzed. It describes the challenges involved and the strategies reported for the development of selective, sensitive, and disposable electrochemical biosensors with the details of fabrication, functionalization, and applications of hybrid metallic composite nanostructures.

## Graphical abstract

Biomarkers indicate the physiological state of a disease. Accurate and selective measurement of such biomarkers in physiological body fluids is the most prerequisite in the healthcare sector. This review emphasizes the hybrid metallic composite nanostructure‐based electrochemical sensors for five types of crucial biomedical markers relevant to the most acute diseases namely, cancer, cardiac, inflammatory, diabetic, and renal biomarkers.

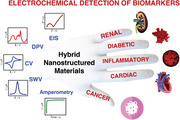

## 1 Introduction

Biosensors are indispensable research tools in the medical diagnostics as they can analyze biomolecular interactions and quantify biomolecules. IUPAC defines the biosensor as “a device that uses specific biochemical reactions mediated by isolated enzymes, immune systems, tissues, organelles or whole cells to detect chemical compounds usually by electrical, thermal or optical signals.”^[^ ^1^ ^]^ There are two elementary constituents linked in series: a chemical (biomolecular) recognition system (receptor) and a physicochemical transducer. Biosensors provide specific quantitative and semiquantitative analytical information using a biological recognition element, which is in direct spatial contact with a transduction element. In addition to these components, biosensors also include signal amplification, control, and processing components for the assurance of efficient signal transduction. The major attributes of a good biosensor system are specificity, sensitivity, reliability, possibility of miniaturization, portability, real‐time analysis, and user‐friendly operation. The field of biosensors is significantly developed from the last decade, because it offers simple, rapid, cost‐effective, and field‐portable screening methods. Many biosensors reported in the literature indicated that these devices are promising and quite effective analytical tools. In today's market, only a few biosensors are commercially available (e.g., sensors for microbial contamination, glucose, and biological oxygen demand), while many are still in the development stage for commercial application.

### 1.1 Biomarkers: Categories

Biomarker is a substance, which indicates the physiological state of a disease and helps to arrive at the exact disease mechanism thereby helps to diagnose and treat specific disease at a given time. Origin and classification of biomarkers for various disease diagnoses were explicitly described by numerous researchers.^[^ ^2^, ^3^, ^4^, ^5^ ^]^ Accurate and specific detection of biomarkers leads to disease prevention, better patient health outcomes, and importantly reduced healthcare costs. Diagnosis of the most severe diseases involves the selective quantification of clinically relevant biomolecules such as glucose, H_2_O_2_, superoxide, ascorbic acid, uric acid, and neurotransmitters along with the other significant biomarkers. Simple, trouble‐free, selective, and accurate measurement of such biomarkers in physiological body fluids is the most prerequisite in the healthcare sector.

This review article aims to bring together the recent research works reported on five types of the below selected crucial biomarkers relevant to the most acute diseases—namely, cancer, cardiac, inflammatory, diabetic and renal biomarkers. The following subsections present the important review articles reported on these selected biomarkers.

#### 1.1.1 List of Selected Cancer Biomarkers

Specific quantification of cancer biomarkers can be helpful in the early cancer detection, categorization, severity level, and assessment of resistance to chemotherapy.^[^ ^6^, ^7^, ^8^, ^9^, ^10^, ^11^ ^]^ Precise recognition of prostate‐specific antigen (PSA) helps to examine prostate cancer,^[^ ^12^, ^13^, ^14^, ^15^ ^]^ carcinoembryonic antigen (CEA) for colon cancer,^[^ ^16^, ^17^, ^18^, ^19^, ^20^ ^]^ *α*‐fetoprotein (AFP) for liver cancer,^[^ ^11^, ^21^, ^22^, ^23^, ^24^ ^]^ neuron‐specific enolase (NSE) for lung cancer,^[^ ^25^, ^26^, ^27^, ^28^, ^29^ ^]^ and ferritin for breast and pancreatic cancers.^[^ ^30^, ^31^, ^32^, ^33^, ^34^, ^35^ ^]^

#### 1.1.2 List of Selected Cardiac Biomarkers

Accurate measurement of cardiac biomarkers will pave a better platform for the on‐site healthcare diagnostics.^[^ ^36^, ^37^, ^38^, ^39^, ^40^, ^41^, ^42^, ^43^ ^]^ Human cardiac troponin (troponin I, T, and C) is the crucial cardiac biomarker for myocardial infarction.^[^ ^44^, ^45^, ^46^, ^47^, ^48^, ^49^ ^]^ Myoglobin is a biomarker (normal levels in serum 30–90 ng mL^−1^) for detecting acute myocardial infarction.^[^ ^29^, ^50^, ^51^, ^52^, ^53^, ^54^ ^]^ Myoglobin together with troponin is used as cardiac biomarker in the diagnosis of heart attack.^[^ ^29^, ^55^ ^]^ Superoxide radical and superoxide dismutase^[^ ^56^, ^57^, ^58^, ^59^, ^60^, ^61^ ^]^ are quantified majorly in monitoring the diagnosis of heart attack. Myeloperoxidase helps to understand the cause behind sudden cardiac death.^[^ ^62^, ^63^, ^64^, ^65^, ^66^ ^]^ It is well known that selective quantification of thrombin helps to diagnose the patients with myocardial infarction. Though thrombin is not present in blood under normal conditions, its inactive form prothrombin is secreted into blood at a concentration of 1.2 × 10^−6^ m. It is reported that the *α*‐thrombin is much more active than the other proteolyzed forms of thrombin (*β* and *γ*).^[^ ^67^, ^68^, ^69^, ^70^, ^71^ ^]^

#### 1.1.3 List of Selected Inflammatory Disease Biomarkers

The word inflammation means “to set on fire” which was derived from the Latin word inflammare. German pathologist Rudolf Virchow defined the inflammation as the loss of function. Inflammatory biomarkers offer important information related to the chronic obstructive pulmonary diseases,^[^ ^72^, ^73^, ^74^ ^]^ Alzheimer's disease,^[^ ^75^ ^]^ and asthma.^[^ ^76^ ^]^ Nitric oxide^[^ ^77^, ^78^, ^79^, ^80^, ^81^, ^82^ ^]^ is the active radical species, which is recognized as one of the major inflammatory disease biomarkers. Tumor necrosis factor alpha (TNF‐*α*) also known as cachectin is one of the key inflammatory biomarkers.^[^ ^83^, ^84^, ^85^, ^86^, ^87^ ^]^ C‐reactive protein (CRP)^[^ ^88^, ^89^, ^90^, ^91^, ^92^ ^]^ in serum is considered as the biomarker for inflammation associated with cardiovascular diseases.^[^ ^89^ ^]^ Interleukins (IL) existed in several types, among which IL‐6 is considered as the most analyzed biomarker for the inflammatory disease.^[^ ^93^, ^94^, ^95^, ^96^, ^97^, ^98^, ^99^ ^]^

#### 1.1.4 List of Selected Diabetic Biomarkers

Several research reports are available on the selective detection of diabetic biomarkers.^[^ ^100^, ^101^, ^102^, ^103^, ^104^ ^]^ Selective measurement of blood glucose is the standard for monitoring diabetes mellitus.^[^ ^105^, ^106^, ^107^, ^108^, ^109^, ^110^ ^]^ Frequent testing of physiological glucose levels is critical to confirm that treatment is working effectively and to avoid a diabetic emergency.^[^ ^111^ ^]^ Glycated hemoglobin (HbA1c)^[^ ^112^, ^113^, ^114^, ^115^, ^116^, ^117^, ^118^, ^119^ ^]^ is another gold standard for the monitoring of diabetes. 2‐aminoadipic acid has also been reported as a potential biomedical marker for the prediction of diabetes risk.^[^ ^120^ ^]^

#### 1.1.5 List of Selected Renal Biomarkers

Renal biomarkers indicate the exact functioning status of kidneys.^[^ ^121^, ^122^ ^]^ More than 350 biomarkers have been tested for acute kidney injury. However, the most promising candidates in this category such as neutrophil gelatinase‐associated lipocalin (NGAL), KIM, IL‐18, and L‐FABP exhibit conflicting and controversial results and are still far from implication into clinical practice. Serum creatinine is the most tested renal biomarker.^[^ ^123^, ^124^, ^125^, ^126^, ^127^, ^128^ ^]^

Core theme of this review is to present the latest trend in the detection of chosen vital biomarkers and to emphasize the advances in electrochemical sensor systems, which utilized hybrid nanostructures as the recognition matrices. The following section explains the characteristics and advantages of the hybrid nanostructures.

### 1.2 Hybrid Nanostructured Materials as the Recognition Elements

IUPAC has defined the hybrid material as “material composed of an intimate mixture of inorganic components, organic components, or both types of components. The components usually interpenetrate on scale of less than 1 µm.”^[^ ^129^ ^]^ IUPAC has defined the nanocomposite as “composite in which at least one of the phase domains has at least one dimension of the order of nanometers.”[^129^, ^130^, ^131^ ^]^ Hybrid nanostructures offer distinct advantages compared to the individual components, and at the same time may exhibit new properties and functions for practical applications.^[^ ^132^ ^]^ Their enhanced properties arise from the synergism between different components due to increased interactions among them, the large common interface owing to similar dimensions, and the changed dynamics of the charge carriers in the resultant hybrid nanostructures.^[^ ^133^ ^]^

The components of a hybrid nanostructure can be chosen from a wide range of materials such as enzymes,^[^ ^134^, ^135^ ^]^ antibodies,^[^ ^136^, ^137^ ^]^ nucleic acids,^[^ ^138^, ^139^, ^140^ ^]^ aptamers,^[^ ^141^, ^142^ ^]^ peptides,^[^ ^143^ ^]^ dendrimers,^[^ ^144^, ^145^ ^]^ ion channels,^[^ ^146^, ^147^ ^]^ calixarenes,^[^ ^148^, ^149^ ^]^ hydrogels,^[^ ^150^, ^151^ ^]^ self‐assembled monolayers,^[^ ^152^, ^153^ ^]^ organic conducting polymers,^[^ ^154^, ^155^ ^]^ molecular imprinted polymers,^[^ ^156^, ^157^ ^]^ mesoporous materials,^[^ ^158^, ^159^ ^]^ metal–organic frameworks,^[^ ^160^, ^161^ ^]^ silica materials,^[^ ^162^, ^163^, ^164^, ^165^ ^]^ fibers,^[^ ^166^ ^]^ quantum dots,^[^ ^167^, ^168^ ^]^ magnetic nanomaterials,^[^ ^169^, ^170^, ^171^, ^172^, ^173^ ^]^ metallic/mixed metallic nanoparticles,^[^ ^174^, ^175^, ^176^, ^177^, ^178^, ^179^, ^180^, ^181^, ^182^, ^183^ ^]^ 2D inorganic nanomaterials (boron nitride, black phosphorous),^[^ ^184^ ^]^ carbonaceous materials (CNT,^[^ ^185^, ^186^, ^187^ ^]^ graphene,^[^ ^188^, ^189^ ^]^ graphene oxide derivatives^[^ ^190^, ^191^ ^]^), etc.

Hybrid nanostructures exhibit much better characteristics like enhanced active surface area, excellent adsorption ability,^[^ ^192^ ^]^ facile biomolecular conjugation, improved conductivity,^[^ ^193^ ^]^ enzyme mimicking with peroxidase like activity,^[^ ^194^ ^]^ and electrocatalytic activity.^[^ ^195^, ^196^, ^197^ ^]^ Hybrid nanostructures have been utilized as nanocarriers^[^ ^198^, ^199^, ^200^ ^]^ and immune probes^[^ ^201^, ^202^, ^203^ ^]^ toward the detection of biomarkers. Such characteristics of the hybrid nanostructures bring about superior detection limits (sub femtomolar levels^[^ ^204^, ^205^, ^206^ ^]^) in a wide concentration range, high selectivity,^[^ ^207^, ^208^ ^]^ very good stability and reproducibility to the resultant sensor systems. The enhanced catalytic activity due to the presence of metal nanoparticles, mixed metal derivatives (oxide/sulfide/selenide), and improved electrochemical conductivity of carbon nanomaterials (functionalized CNT/graphene) can act synergistically, and as a result, the hybrid nanostructures offer promising results in the fabrication of highly selective electrochemical sensor systems.

This review summarizes electrochemical sensor systems constructed with hybrid metallic composite nanostructures as the recognition matrices in which the predominant inorganic components are—single/bi/mixed metallic nanoparticles or metal chalcogenide derivatives; and the organic components are—antibodies, aptamers, polymers, biomolecules, and the carbon nanomaterial derivatives.

## 2 Objectives and Scope of the Present Review

Ample range of literature review was performed on various types of transduction methods and innovative approaches in constructing novel molecular recognition elements for the above selected vital biomedical marker molecules. A variety of recognition elements reported for the detection of selected biomarkers have been compiled. Six different transduction approaches reported toward the selective quantification of crucial biomarkers were summarized scrupulously to present an overview of the current research works. The review further focused predominantly on the electrochemical sensors, which used highly selective and highly stable advanced hybrid metallic composite nanostructures. This review emphasizes the recent trend in the innovative schemes of preparing hybrid nanostructures. Strategies resulting to enhanced sensitivity, high selectivity, reduced analytical times, and reusability have been proposed. Transduction methodologies, nanocomposite materials, and electrode fabrication methods leading to the development of efficient electrochemical sensor systems have been discussed.

The major objectives of this review are to summarize as follows:

- research progress in the selective detection of cancer, cardiac, inflammatory, diabetic, and renal biomarkers,
- synthetic strategies involved in the advanced hybrid metallic composite nanostructures
- electrochemical sensors developed using amperometry, cyclic voltammetry, differential pulse voltammetry, square wave voltammetry, and electrochemical impedance spectroscopy, and
- applicability of electrochemical sensor systems for the detection of vital biomarkers selectively from physiological samples—serum, urine, saliva, etc.

**Figure 1:**
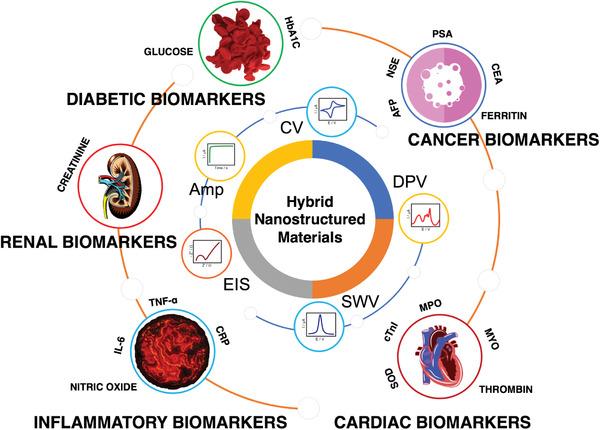
Electrochemical detection of vital biomarkers using the advanced hybrid nanostructured materials—a schematic representation.

Though there are several review articles reported on the theme of electrochemical sensors^[^ ^209^, ^210^ ^]^ for the detection of biomarkers,^[^ ^211^, ^212^, ^213^ ^]^ most of them are limited to either one specific biomarker,^[^ ^79^, ^80^, ^118^, ^214^, ^215^ ^]^ one type of biomarkers,^[^ ^9^, ^10^, ^20^, ^212^, ^216^, ^217^, ^218^, ^219^, ^220^ ^]^ one specific transduction,^[^ ^221^, ^222^, ^223^ ^]^ one type of electrode^[^ ^224^, ^225^, ^226^ ^]^ or one type of recognition materials.^[^ ^39^, ^188^, ^227^, ^228^, ^229^, ^230^, ^231^, ^232^ ^]^ In this aspect, the present review helps to understand collectively the recent literature reports on the electrochemical sensors of a set of five different crucial biomedical markers based on advanced hybrid metallic composite nanostructures as recognition materials. The core theme of review is expressed in the form of a schematic representation (**Figure** **1**).

This review will help the budding researchers working in the areas of electrochemical sensors and materials chemistry to come up with new innovative combinations of metal nanoparticles, functionalized nanocomposite materials, and other biomolecules to develop electrochemical sensors of reusability and on‐site analysis for highly necessitated biomarkers as well as other critical target analytes.

## 3 Different Transduction Approaches toward the Detection of Biomarkers

Laboratory‐based sensors for different biomolecules were reported using wide variety of transduction methods such as UV–vis spectroscopy,^[^ ^233^ ^]^ Fourier transform infrared spectroscopy,^[^ ^234^ ^]^ Raman spectroscopy,^[^ ^235^ ^]^ surface‐enhanced Raman spectroscopy (SERS),^[^ ^184^ ^]^ capillary electrophoresis,^[^ ^236^ ^]^ chemiluminescence,^[^ ^237^ ^]^ electrochemiluminescence,^[^ ^238^ ^]^ fluorescence spectroscopy,^[^ ^239^ ^]^ field‐effect transistor,^[^ ^240^ ^]^ quantum photonic sensing,^[^ ^143^ ^]^ plasmonic nanopore sensing,^[^ ^240^ ^]^ surface plasmon resonance (SPR),^[^ ^241^ ^]^ gas chromatography,^[^ ^242^ ^]^ high performance liquid chromatography,^[^ ^243^ ^]^ liquid chromatography coupled with mass spectrometry, and quartz crystal micro/nanobalance.^[^ ^244^ ^]^

The following subsections present an overview of six different transduction methods reported toward the detection of crucial biomedical markers particularly which include hybrid nanostructures in the recognition matrix. A number of review articles have described the research progress in the detection of above selected biomarkers using the transduction methods such as fluorescence spectroscopy,^[^ ^245^, ^246^, ^247^, ^248^ ^]^ chemiluminescence,^[^ ^249^ ^]^ electrochemiluminescence,^[^ ^250^ ^]^ electrophoresis,^[^ ^251^, ^252^, ^253^ ^]^ SPR,^[^ ^254^, ^255^, ^256^, ^257^ ^]^ and SERS.^[^ ^258^, ^259^, ^260^, ^261^ ^]^

### 3.1 Fluorescence Spectroscopy

A fluorescent biosensor was constructed for the detection of PSA using biocompatible CdTe@SiO_2_ core–shell nanoparticles as labels and PSA antibody–functionalized magnetic Fe_3_O_4_ nanoparticles (Fe_3_O_4_–Ab1) as the capturing probes. The captured PSA was then immunorecognized by CdTe@SiO_2_ labeled with PSA detection antibodies (CdTe@SiO_2_–Ab2) by forming the sandwich complex Fe_3_O_4_–Ab1/PSA/Ab2–CdTe@SiO_2_. The proposed immunosensor exhibited good selectivity to PSA with an LOD value of 3 pg mL^−1^ in the concentration range of 0.01 to 5 ng mL^−1^.^[^ ^262^ ^]^ Another fluorescent sensor for PSA was reported with antibody functionalized CdTe QDs as label and aptamer decorated polyamidoamine–Au nanoparticles (NPs) as capturing probe. A sandwich immunocomplex formed between PSA and aptamer, i.e., QDs‐Ab/PSA/AuNPs–PAMA/aptamer has facilitated the detection of PSA by decreasing the fluorescence intensity of CdTe QDs with PSA addition. A superior sensitivity of 1 pg mL^−1^ was achieved in the linear range of 0.01–100 ng mL^−1^ PSA. Presence of aptamer increased the selectivity toward PSA whereas the CdTe QDs along with dendrimer enhanced the sensitivity.^[^ ^263^ ^]^

**Figure 2:**
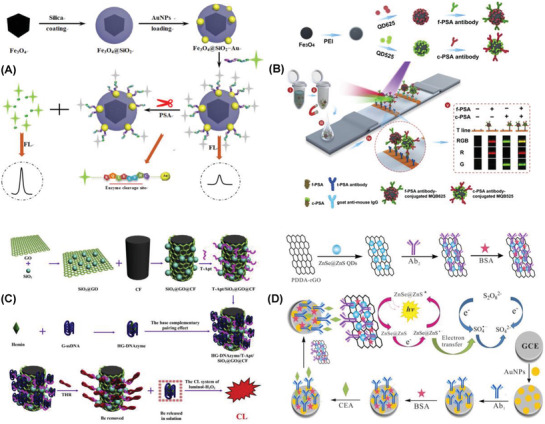
Fluorescent detection of PSA using A) 5‐FAM labeled peptide/Fe_3_O_4_@SiO_2_–Au nanocomposite. Reproduced with permission.^[^ ^264^ ^]^ Copyright 2018, Elsevier. B) Fe_3_O_4_@PEI@QDs composite. Reproduced with permission.^[^ ^265^ ^]^ Copyright 2019, Elsevier. C) Chemiluminescent detection of thrombin using HG‐DNAzyme/T‐Apt/SiO_2_@GO@CF composite. Reproduced with permission.^[^ ^266^ ^]^ Copyright 2018, Elsevier. D) Electrochemiluminescent detection of CEA using PDDA–rGO/ZnSe@ZnS QDs composite. Reproduced with permission.^[^ ^267^ ^]^ Copyright 2019, Elsevier.

A fluorescent biosensor constructed with 5‐FAM labeled peptide/Fe_3_O_4_@SiO_2_–Au nanocomposite has offered the detection limit of 0.3 pg mL^−1^ PSA in the range of 1 pg mL^−1^–1 ng mL^−1^. In situ growth of Au nanoparticles on the SiO_2_ encapsulated single Fe_3_O_4_ nanocubes led to the formation of the hybrid nanocomposite which displayed robust salt stability, easy magnetic separation, and eventually minimized the background fluorescence (**Figure** **2**A). The PSA specifically recognized and cleaved the 5‐FAM‐labeled peptides leading to the fluorescence recovery.^[^ ^264^ ^]^

Fluorescent detection of PSA was reported using a hybrid core–shell composite of Ag@SiO_2_@SiO_2_–RuBpy. The sensor system worked based on metal‐enhanced fluorescence and magnetic separation using the immunomagnetic nanospheres and immunofluorescent nanoparticles which helped to capture and identify the target molecules. The observed‐enhanced fluorescence intensity was attributed to the interaction between the doped RuBpy molecules in the outer silica layer and the silver core. It was reported that larger amounts of RuBpy was incorporated into the silica shell as the self‐quenching of RuBpy was minimized in the presence of silica. The detection limit of PSA was 27 pg mL^−1^ in the concentration range of 0.1–100 ng mL^−1^.^[^ ^268^ ^]^

An ultrasensitive fluorescent sensor system was constructed for PSA using graphene oxide quantum dots@silver (GQDs@Ag) core–shell nanocrystals as the sensor probe and magnetic beads–PSA antibody (MBs–Ab1) as the capture probe. The critical part of the sensor system was the assembly of more GQDs in one GQDs@Ag probe as the ratio of probe to target significantly increased, which ultimately enhanced the fluorescent signal. H_2_O_2_ led to the removal of silver shell by oxidative etching and released the incorporated GQDs more readily which in turn caused little change to the fluorescence. LOD of the resultant immunosensor was 0.3 pg mL^−1^ PSA in the range of 1 pg mL^−1^–20 ng mL^−1^.^[^ ^269^ ^]^

A low‐cost and portable smartphone readout device was developed for the fluorescent detection of free and complexed PSA using red and green magnetic‐quantum dot nanobeads. The fluorescent probes were constructed with Fe_3_O_4_ magnetic cores which covered with polyethyleneimine‐mediated electrostatic adsorption of numerous quantum dots and further conjugated with free and complexed PSA antibodies (Fe_3_O_4_@PEI@QDs) (Figure 2B). Fluorescent imaging was achieved by incorporating the above fluorescent probes in test strip and t‐PSA antibody on the test line. The observed detection limits were 9 and 87 pg mL^−1^ of free and complexed PSA, respectively. The sensor system can be a promising point‐of‐care diagnostics technique for the accurate diagnosis of prostate cancer even in resource‐limited settings.^[^ ^265^ ^]^ Potential application of all these fluorescent sensors has been demonstrated in the analysis of clinical serum samples.

### 3.2 Chemiluminescence

An interesting “signal‐on” chemiluminescence thrombin biosensor was constructed with aptamer functionalized magnetic sodium alginate (Ca^2+^‐Malg‐Apt1) hydrogel which helped in separating and enriching thrombin. Chelation between ethylenediamine tetraacetic acid (EDTA) and Ca^2+^ caused to release thrombin by dissolving the hydrogel. Further, the chemiluminescent signal was amplified with metalloporphyrinic metal–organic framework nanosheet and DNA–functionalized gold nanoparticles Cu‐TCPP(Co) MOFs/Au–ssDNA. The recognition matrix was thrombin aptamer2‐functionalized magnetic carbon nanotubes (MCNTs‐Apt2), which facilitated the adsorption of Cu‐TCPP(Co) MOFs/Au‐ssDNA. Thrombin presence triggered the release of Cu‐TCPP(Co) MOFs/Au‐ssDNA, which generated the chemiluminescent signal. The observed LOD value was 0.2 × 10^−12^ m thrombin in the range of 0.89 × 10^−12^ m–0.59 × 10^−9^ m with an excellent selectively.^[^ ^270^ ^]^

Femtomolar sensitive detection of thrombin was reported using the hybrid recognition matrix of aptamer‐conjugated and hemin/G‐quadruplex DNAzyme signal‐amplified carbon fiber composite (HG‐DNAzyme/T‐Apt/SiO_2_@GO@CF). The presence of thrombin led to the desorption of HG‐DNAzyme from the surface of T‐Apt/SiO_2_@GO@CF which in turn catalyzed the system of luminol‐H_2_O_2_ (Figure 2C). The observed LOD value was 6.3 × 10^−15^ m in the linear concentration range of 25 × 10^−12^ m to 15 × 10^−15^ m.^[^ ^266^ ^]^

Another hybrid recognition matrix of chitosan‐modified magnetic oxide graphene composite along with thrombin aptamer and iron porphyrin hemin CS@Fe_3_O_4_@GO@T‐Apt@HM was investigated toward the chemiluminescent detection of thrombin. The hybrid composite catalyzed the system of luminol–H_2_O_2_, which generated the signal. The existence of thrombin in solution caused the desorption of HM from the hybrid composite, which in turn led to the signal response. The observed LOD value was 1.5 × 10^−15^ m thrombin in the range of 250 × 10^−12^ m–5 × 10^−15^ m. All these hybrid composite‐based recognition matrices have been examined in serum samples which revealed the potential application toward thrombin detection in monitoring and diagnosis of human blood diseases.^[^ ^271^ ^]^

Simultaneous chemiluminescent detection of three cardiac biomarkers was reported with a 3D microfluidic paper‐analytical device (µPAD). Hybrid composite of Co^2+^/*N*‐(aminobutyl)‐*N*‐(ethylisoluminol)–Fe_3_O_4_@void@C‐CS/Au–Ab^[^ ^272^ ^]^ as the sensing probe and Ab1–Au/antigen/Co(II)–Ab2–luminol–Au as the amplified sensing probe^[^ ^273^ ^]^ offered detection limits toward cTnI as 0.50 and 0.40 pg mL^−1^, respectively. The proposed immunoassays claimed to be promising materials in early diagnosis and treatment of acute myocardial infarction.

### 3.3 Electrochemiluminescence

Electrochemiluminescence detection of cancer biomarker CEA was reported with AuNPs as a sensing platform and poly(diallyldimethylammonium chloride), reduced graphene oxide, zinc selenide–zinc sulfide quantum dots (PDDA–rGO/ZnSe@ZnS QDs) composite as the signal probe (Figure 2D). The hybrid composite provided larger activity sites which improved the sensitivity and stability of the resultant immunosensor. The observed LOD was 0.029 pg mL^−1^ CEA in a range of 0.1 pg mL^−1^–100 ng mL^−1^.^[^ ^267^ ^]^

In another report, a hybrid nanocomposite of magnetite‐silica‐polystyrene (Fe_3_O_4_@SiO_2_@PS) was examined as magnetic separable carrier toward the selective electrochemiluminescence immunosensing of CEA. CdTe QDs‐embedded mesoporous silica nanospheres (mSiO_2_/CdTe) were used as the signal probe, which encapsulated hundreds of QDs leading to the enhanced electrochemiluminescence intensity. The observed LOD value was 0.3 pg mL^−1^ in the range of 0.001 to 80 ng mL^−1^.^[^ ^274^ ^]^ A convenient, rapid and ultrasensitive electrochemiluminescence detection of CEA was achieved using ferrocene labeled Ru(bpy)_3_^2+^–SiO_2_ nanocomposite as the signal probe. Hybrid recognition matrix of magnetic core–shell Fe_3_O_4_@Au nanoparticles with DNA1 was used as the carrier platform as part of the cascade signal amplification strategy. The resultant immunosensor system was able to detect 3.5 fg mL^−1^ CEA in the range of 10 fg mL^−1^ to 10 ng mL^−1^.^[^ ^275^ ^]^

A simple and label‐free electrochemiluminescence detection of CEA was reported using the nanocomposite of ZnS–CdS@MoS_2_/chitosan along with the aptamer deposited on glassy carbon electrode. The resultant aptasensor offered an LOD value of 0.031 ng mL^−1^ in the linear range of 0.05–20 ng mL^−1^.^[^ ^276^ ^]^ Both these sensors were tested in the blood serum and displayed very good recovery limits of CEA.

An ultrasensitive label‐free electrochemiluminescence CEA sensor system was reported using the hybrid composite of porous platinum (Pt) nanostructures on ionic liquid‐functionalized graphene film (GR‐IL/pPt) as the platform and luminol as the signal probe. Biocompatibility, excellent electrocatalytic activity, and highly porous structure of the hybrid composite offered high loading density of the antibody which in turn amplified the signal response. The resultant sensor system displayed an extreme LOD of 0.0003 fg mL^−1^ CEA in the range of 0.001 fg mL^−1^ to 1 ng mL^−1^.^[^ ^277^ ^]^ All these hybrid nanocomposite‐based sensor systems have proven the potential application in clinical diagnostics.

### 3.4 Electrophoresis

Pathological situations lead to the modifications in size and/or electrical charge of the glycoproteins which can be easily monitored by capillary zone electrophoresis (CZE) because the size to charge ratio of the analyte generates differences in migration in turn altering the electrophoretic pattern. Glycoforms are the isoforms of glycoprotein. Enhanced CZE pattern of PSA was achieved using two complementary strategies by conditioning capillaries with HCl and optimizing the pH of background electrolyte to 8.0 along with 3 m urea on its composition.^[^ ^278^ ^]^

PSA in serum is the most used prostate cancer marker, but the limitation is its specificity. PSA specificity can be enhanced by monitoring the glycosylation change as the prostate cancer alters the PSA glycosylation. CZE and/or 2D electrophoresis (2DE) can be applied as the PSA glycosylation changes leads to the variations in PSA electrophoretic behaviour. Serum PSA is mostly complexed with  *α*‐1 antichymotrypsin, which must be released as free PSA and the total free PSA must be purified from the serum matrix before CZE analysis. A sample treatment approach was illustrated with ethanolamine for successfully isolating PSA from serum without altering the circular dichroism (CD) spectrum or the CZE pattern of PSA standard. Direct comparison of the electropherograms was feasible by choosing the effective electrophoretic migration instead of migration time as the migration parameter. The methodology was validated by demonstrating the separation of PSA from serum of a cancer patient with a high PSA content.^[^ ^279^ ^]^ Comparative performance of CE over 2DE was examined for resolving PSA subforms in seminal plasma and urine of a prostate cancer patient. Variations in the post‐translational modifications led to different PSA spots by 2DE as well as different CE profiles. This study revealed that CE and 2DE are both complementary to each other for PSA analysis.^[^ ^280^ ^]^

Simultaneous detection of multiple biomarkers is necessary in the cancer diagnosis. Microfluidic chip electrophoresis (MCE) detection of three cancer biomarkers AFP, CEA, and carbohydrate antigen 199 (CA199) was reported using endonuclease‐linked multiplex immunoassay. Various sandwich immunocomplexes of the selected cancer markers were formed by the interactions between the primary antibodies and the endonuclease labeled secondary antibodies which were incubated in 96‐pore plate. MCE detection was based on the separated fragments of DNA substrate strands, which were altered by the corresponding endonuclease. The observed detection limits were 0.35, 0.3 pg mL^−1^ for AFP, CEA, and 0.36 U mL^−1^ for CA199 in the concentration range of 1 pg mL^−1^ to 10 ng mL^−1^ (U mL^−1^ for CA199).^[^ ^281^ ^]^ The detection limits were further improved to 0.1, 0.2, 0.15 pg mL^−1^ of AFP, CEA, and carbohydrate antigen 125, respectively, using a catalytic hybrid assembly of aptamer‐functionalized magnetic beads (Fe_3_O_4_@AuNPs). Signal tags were constructed by single‐stranded DNA primers labeled with antibodies. Antibody‐tumor marker–aptamer sandwich complexes were formed by the interactions between the aptamer‐functionalized magnetic beads and the above signal tags. After magnetic separation, three pairs of hairpins as substrates were introduced to trigger catalytic hybrid assembly by the primers in the complex which led to many duplex DNA products of different length in the supernatant. The influencing parameters such as hairpin concentration, reaction time, and temperature were systemically optimized.^[^ ^282^ ^]^ Practical utility of both these materials was demonstrated successfully in human serum samples. These demonstrations proved that MCE can detect many such diagnostic markers.

### 3.5 Surface Plasmon Resonance

Selective detection of human cardiac troponin‐I (cTnI) was reported with immunomagnetic separation technology‐assisted SPR. AuNPs–polydopamine (PDA)–cAb/Au film as the capturing platform and Fe_3_O_4_–PDA–dAb as the sensing probe have offered the precise capture and magnetic separation of cTnI by minimizing nonspecific interference from complex matrices. The signal response was further enhanced with the introduction of MWCNTs–PDA–AgNPs/Ab2. The observed detection limit was 3.75 ng mL^−1^ cTnI in the range of 15–2500 ng mL^−1^ which was 320‐fold lower compared to that of PDA‐based assay.^[^ ^201^ ^]^ Hollow AuNPs‐PDA‐cAB/Au film on a glass slate as the capturing platform and magnetic MMWCNTs–PDA–dAb as the sensing probe were used toward the SPR detection of cTnI. Small variations in the capturing platform and the sensing probes led to much improved detection limits of cTnI. The observed detection limit was 1.25 ng mL^−1^ cTnI in the range of 15–2500 ng mL^−1^, which was 1000‐fold lower compared to that of PDA‐modified gold film. The improved SPR response was attributed to the electronic coupling of the surface plasmon waves originating from the hollow AuNPs and Au film.^[^ ^283^ ^]^

**Figure 3:**
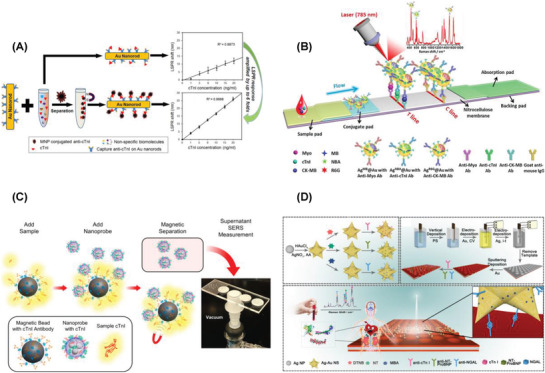
A) SPR detection of cTnI using Fe_3_O_4_ MNPs on Au nanorods. Reproduced with permission.^[^ ^284^ ^]^ Copyright 2013, American Chemical Society. Selective detection of cTnI using B) Ag^MB^@Au, Ag^NBA^@Au, and Ag^R6G^@AuNPs as the SERS probes. Reproduced with permission.^[^ ^293^ ^]^ Copyright 2018, Elsevier. C) Silica encapsulated Ag–DTNB nanoparticles as SERS nanotags. Reproduced with permission.^[^ ^294^ ^]^ Copyright 2017, American Chemical Society. D) Ag–Au nanostars@4‐MBA as SERS nanotags. Reproduced with permission.^[^ ^295^ ^]^ Copyright 2016, American Chemical Society.

A facile and ultrasensitive label‐free SPR detection of cTnI was reported using Fe_3_O_4_ magnetic nanoparticles and gold nanorods. The detection limit of 30 × 10^−12^ m was claimed as the lowest LOD for plasma cTnI based on label‐free SPR without complicated instrumentation (**Figure** **3**A). The robust and low‐cost instrument was promising in developing miniaturized lab‐on‐a‐chip sensor system for the point‐of‐care medical diagnostics. This study successfully demonstrated the advantages of SPR compared to fluorescence spectroscopy or magnetic resonance imaging with a low cost, clinical‐oriented detection of cTnI.^[^ ^284^ ^]^ SPR detection of cTnI was reported with biocompatible and water dispersible Fe_3_O_4_ magnetic nanoparticles on gold nanorod biochip. Sodium oleate (NaOL) not only facilitated the water dispersion of Fe_3_O_4_ MNPs, but also improved the biofunctionalization of the resultant aqueous MNPs. Superparamagnetism of the monodispersed MNPs varied slightly. The biofunctionalized MNPs displayed the enhanced plasmonic response of gold nanorods (GNRs) with varying concentrations of cTnI in the range of 2.5 to 30 ng mL^−1^. SPR sensitivity was three times better with MNPs compared to that of without functional MNPs. Further, the computational simulation studies revealed that the enhancement was distance dependent between the MNP and the surface of the GNRs.^[^ ^285^ ^]^

An ultrasensitive detection of cTnI was reported based on the SPR‐enhanced light scattering of AuNPs in a sandwich assay. AuNPs were functionalized with CALNN‐Peg4‐FYSHSFHENWPS peptide and the spacer CALNN. The calculated detection limit of cTnI was 0.2 ng mL^−1^. Theoretical simulations conveyed that the inherently higher field intensity for SPR was observed at low AuNPs densities.^[^ ^286^ ^]^

SPR performance of GNRs functionalized with two different crosslinkers was examined toward the detection of cTnI. Functionalization of GNRs with cetyltrimethyl‐ammoniumbromide (CTAB) and 11‐mercaptoundecanoic acid (MUDA) led to the formation of MUDA–GNRs and CTAB‐capped GNRs. The detection limit of cTnI was 1 ng mL^−1^ (30 × 10^−12^ m) with MUDA–GNRs, which was five times better compared to that of CTAB‐GNRs. The reason was attributed to the fact that the carboxyl groups facilitate the covalent conjugation of biological receptors and the smaller MUDA layers on the GNRs reduce the distance of target binding to the plasmonic nanostructure interface which in turn enhance the resultant SPR assay sensitivity and specificity.^[^ ^287^ ^]^

A label‐free immunosensor was constructed for the detection of AFP using the hetero‐structured Au–ZnO flower‐rods. It was reported that the visible light absorption of ZnO FRs was enhanced with the coexistence of AuNPs because of SPR and further improved the separation of photogenerated electron–hole pairs as well. Photocurrent of Au–ZnO FRs was increased under simulated sunlight. The photocurrent was reduced after the specific antibody‐antigen immune reaction. Based on the variation of photocurrent with AFP antigen concentration in the range of 0.005–50 ng mL^−1^ the LOD was calculated as 0.56 pg mL^−1^. The immunosensor was stable and exhibited very good anti‐interference property.^[^ ^288^ ^]^ These sensor systems demonstrated the potential application of SPR in clinical diagnosis.

### 3.6 Surface‐Enhanced Raman Spectroscopy

Raman spectroscopy helps to understand the conformational and structural changes of proteins. Selective detection of the cardiac marker myoglobin was reported with SERS using a nanohybrid of aptamer functionalized AuNPs on tungsten disulfide (WS_2_) nanosheets. In‐situ synthesis was applied to the preparation of nanohybrid plasmonic hotspots. SERS signal was generated using a 532 nm laser. The detection limit of myoglobin was 10 fg mL^−1^ in the concentration range of 10 fg mL^−1^–0.1 µg mL^−1^. The fabricated aptasensor has demonstrated the synergistic effect of WS_2_ and AuNPs in enhancing the SERS signal.^[^ ^289^ ^]^

Label‐free SERS detection of myoglobin was reported using a nanohybrid of 3D silver anisotropic nanopine tree on indium tin oxide (Ag NPT/ITO). Effect of different nanostructures in generating the best SERS signal was examined namely, nanoaggregates, nanorods, and nanobranched Ag/ITO. Numerous hotspots present in the junctions between the central rod and side arms of the 3D morphology of Ag NPT/ITO led to the highest SERS signal response. The resultant SERS sensor offered a detection limit of 10 ng mL^−1^ myoglobin in the range of 10 ng mL^−1^–5 µg mL^−1^.^[^ ^290^ ^]^ Sensor performance was demonstrated in urine samples.

SERS method was used to understand the radiation caused biological injury. AuNPs sputter coated on canonical anodic aluminium oxide nanotip arrays were used as the SERS substrate. The impact of total body irradiation was investigated with the mice serum samples which conveyed that the SERS peak intensity at 532 cm^−1^ increased as a function of duration or dose of total body irradiation. This particular Raman signature was attributed to the myoglobin changes in the muscle damage due to the radiation caused injury. The calculated detection limit of myoglobin was 0.01 ppm in the range of 0.01–1 ppm. The proposed method has been validated by testing the blood and urine specimen of cancer patients who received radiotherapy.^[^ ^291^ ^]^

A lateral flow assay based SERS detection of three cardiac biomarkers myoglobin, cTnI, and creatine kinase‐MB (CK‐MB) was reported. Nile blue A dye (NBA) encapsulated Ag@Au core–shell nanotags (Ag^NBA^@Au) were used as SERS labels. Sensor was constructed using a strip of three test lines. Core–shell SERS nanotags and the high surface area to volume ratio of porous nitrocellulose membrane were the reason behind the enhanced sensor performance. Ultrasensitive detection limits of 3.2, 0.44, and 0.55 pg mL^−1^ myoglobin, cTnI, and CK‐MB was achieved in the range of 0.01–500, 0.01–50, and 0.02–90 ng mL^−1^, respectively. The proposed lateral flow assay method displayed the potential application in point of care testing.^[^ ^292^ ^]^ This work was further extended to understand the effect of three different dyes namely, methylene blue (MB), Nile blue A, Rhodamine 6 G (R6G) by encapsulating in the core–shell nanotags and then immobilizing the resultant nanohybrids on a strip of single test line. Equimolar mixture of Ag^MB^@Au, Ag^NBA^@Au, and Ag^R6G^@AuNPs were employed as the SERS nanotags in the construction of sensor system (Figure 3B). The resultant lateral flow assay‐SERS system offered the detection limits of 4.2, 0.89, and 0.93 pg mL^−1^ for myoglobin, cTnI, and CK‐MB respectively in the concentration range of 0.01–500 ng mL^−1^ myoglobin, 0.01–50 ng mL^−1^ cTnI, and 0.02–90 ng mL^−1^ CK‐MB.^[^ ^293^ ^]^

In another report, lateral flow assay strip based SERS detection of cTnI was demonstrated using three types of citrate‐capped Au@AgNPs, Au@Ag–AuNPs, Ag–AuNPs along with the conventional AuNPs, which were further functionalized with a monoclonal antibody of cTnI. Nile blue A was also used during the preparation of these bimetallic NPs. Activity of all these SERS probes was comprehensively studied by experimental as well as theoretical analysis. Citrate‐capped Au@Ag–AuNPs has exhibited the best SERS activity among all the probes tested. The reason behind the best activity was attributed to the strong electromagnetic enhancement within the interior gap and the availability of large superficial area for the adsorption of Raman‐active molecules. The observed detection limit was 0.09 ng mL^−1^ in the concentration range of 0.09–50 ng mL^−1^ of cTnI.^[^ ^296^ ^]^ Such dye loaded core–shell bimetallic nanotags were called as gap‐enhanced Raman tags (GERTs) or nanomatryoshkas. 1,4‐nitrobenzenthiole (NBT) molecules embedded in a 1 nm gap between core and shell of Au, i.e., AuNR@NBT@Au were investigated as GERTs to address the nonspecific signal observed in the case of above dye loaded core–shell Ag@Au SERS nanotags. Such tags displayed a strong and uniform SERS response than that of other common SERS tags such as Au nanorods, nanostars, Au nanoshells with surface‐adsorbed Raman dyes. AuNR@NBT@Au tags were conjugated with monoclonal cTnI antibody. The resultant SERS sensor offered the detection limit of 0.1 ng mL^−1^ in the concentration range of 0.1–100 ng mL^−1^ cTnI.^[^ ^297^ ^]^

Ultrasensitive SERS detection of cTnI and CK‐MB was reported using a sandwich immunoassay platform of monoclonal‐antibody immobilized gold‐patterned chip. Malachite green isothiocyanate labeled Au@Ag core–shell nanoparticles, i.e., Au(MGITC)@Ag(MGITC) conjugated with polyclonal‐antibody were used as SERS nanoprobes. The observed detection limits were 8.9 pg mL^−1^ cTnI and 9.7 pg mL^−1^ CK‐MB in the concentration range of 0.01–100 ng mL^−1^.^[^ ^298^ ^]^

An interesting collection device was built for the detection of cTnI using magnetic immunoassay and nanoprobes. Ag NPs with the self‐assembled monolayers of 5,5′‐dithiobis(2‐nitrobenzoic acid) (DTNB) were encapsulated in silica, which were further conjugated with cTnI and bovine serum albumin (BSA) using silane‐PEG‐NHS linker to prepare the SERS nanoprobes. Immunoassay was constructed with cTnI antibodies coated on magnetic beads (Figure 3C). The collection device was built using 20 nm pore membranes placed on PDMS layer and acrylic plastic slides. The observed detection limit was 12.9 × 10^−15^ m for the nanoprobe in the range of 27.4 × 10^−15^ m–1.76 × 10^−12^ m. Ultralow detection limits were observed even though the amount of nanoprobes spread on the collection area was very low. The hotspots generated when the Ag NPs encapsulated in silica of each nanoprobe interacted with each other were the major reason behind the enhanced SERS signal of DTNB molecule. In contrast to that, there were less or no hotspots with Ag‐DTNB nanoparticles, which were not encapsulated in silica.^[^ ^294^ ^]^

A sandwich immunoassay platform was constructed for the simultaneous SERS detection of three cardiac biomarkers cTnI, N‐terminal prohormone of brain natriuretic peptide (NT‐ProBNP), and NGAL. Three different Raman nanotags were prepared by labeling Ag–Au nanostars with DTNB, 4‐mercaptobenzoic acid (4‐MBA), and 2‐naphthalenethiol (NT). A 3D ordered macroporous (3DOM) Au–Ag–Au plasmonic array was used as the substrate (Figure 3D). Enhanced SERS signal was observed because of the hot spots generated when these nanotags interact on the substrate. The observed detection limits were 0.76, 0.53, and 0.41 fg mL^−1^ for cTnI, NT‐ProBNP, and NGAL, respectively, in the range of 0.1–10 ng mL^−1^. The practical application of the sensor was demonstrated in human blood plasma from patients.^[^ ^295^ ^]^

SERS detection of NSE was reported with an inexpensive and disposable paper‐based lateral flow strip. SERS probes of Au nanostar@MBA@silica and Au nanostar@MGITC@silica sandwich nanoparticles were used in the immunoassay‐based sensor system. The calculated detection limit was 0.86 ng mL^−1^ NSE in the concentration range of 1.0–75.0 ng mL^−1^. Practical application of the sensor system was demonstrated in clinical blood plasma samples of patients.^[^ ^299^ ^]^

Rapid and sensitive detection of TNF‐*α* was achieved using a magnetic bead pull‐down assay. Three different Raman reporters MBA, DTNB, and TFMBA combined with the silica encapsulated AuNPs were used as the SERS probes. MBA‐based SERS labels in a magnetic bead pull‐down assay offered the LOD of 1 pg mL^−1^ TNF‐*α* in the concentration range of 1 pg mL^−1^ to 10 ng mL^−1^. The reason behind the high sensitivity was attributed to the use of SERS‐active small clusters of AuNPs.^[^ ^300^ ^]^

It was observed from **Table** **1** that the biomarkers can be detected even up to the levels of sub‐fg mL^−1^. The lowest detection limit value of 0.3 pg mL^−1^ PSA was observed with fluorescence spectroscopy using GQDs@Ag core–shell nanocrystals as the recognition matrix. The nanohybrid antigen/BSA/Ab/Au–ZnO flower‐rods have offered the LOD of 0.56 pg mL^−1^ AFP using SPR. The LOD value was further improved to 0.1 pg mL^−1^ AFP with the catalytic nanohybrid Fe_3_O_4_@AuNPs as the recognition matrix and microfluidic chip electrophoresis as transduction. SERS detection with AuNPs–WS_2_/antiMyo/aptamer nanohybrid has led to the LOD of 10 fg mL^−1^ myoglobin, whereas 3DOM Au–Ag–Au plasmonic array with three Raman tags has produced 0.76 fg mL^−1^ cTnI. CS@Fe_3_O_4_@GO@T‐Apt@HM hybrid has produced the LOD of 1.5 × 10^−12^ m thrombin with chemiluminescence. Compared to these techniques, electrochemiluminescence has offered the best LOD value of 0.0003 fg mL^−1^ CEA with GR‐IL/pPt composite. It can be concluded that the hybrid nanostructures comprising metal nanoparticle and/or their derivatives would serve as the excellent recognition matrices.

**Table 1:** Hybrid recognition matrix‐based detection of biomarkers (PSA, thrombin, cTnI, CEA, myoglobin, AFP, NSE, TNF‐*α*) using fluorescence spectroscopy, chemiluminescence, electrochemiluminescence, surface plasmon resonance, and surface‐enhanced Raman spectroscopy: summary

Biomarker	Hybrid recognition matrix	Method	Concentration range	LOD	Interferents	Real samples	Ref.
PSA	Ag@SiO_2_@SiO_2_–RuBpy core–shell nanoparticles	Fluorescence	0.1–100 ng mL^−1^	27 pg mL^−1^	BSA, CEA, AFP, thrombin, IgG	Serum	^[^ ^268^ ^]^
	Fe_3_O_4_@PEI@QDs MQBs	Fluorescence	0.01–100 ng mL^−1^	9 pg mL^−1^	CEA, AFP, CA199, CA125, CA153	Serum	^[^ ^265^ ^]^
	CdTe@SiO_2_/Ab2, Fe_3_O_4_–Ab1–PSA	Fluorescence	0.01–5 ng mL^−1^	3 pg mL^−1^	Phosphor‐p53^15^ antigen, BSA	Serum	^[^ ^262^ ^]^
	Cys‐capped CdTe QDs/anti‐PSA, AuNPs–PAMA/aptamer	Fluorescence	0.01–1 ng mL^−1^	1 pg mL^−1^	BSA, AFP	Serum	^[^ ^263^ ^]^
	5‐FAM labeled peptide/NH_2_–modified Fe_3_O_4_@SiO_2_–Au nanocomposite	Fluorescence	1 pg mL^−1^–1 ng mL^−1^	0.3 pg mL^−1^	MYO, thrombin, LYZ, GOD, *β*‐LAG, BSA	Serum	^[^ ^264^ ^]^
	GQDs@Ag core–shell nanocrystals	Fluorescence	1 pg mL^−1^–20 ng mL^−1^	0.3 pg mL^−1^	IgG, BSA, CA125	Serum	^[^ ^269^ ^]^
Thrombin	Cu–TCPP(Co) MOFs/Au–ssDNA	CL	0.89 × 10^−12^ m–0.59 × 10^−9^ m	0.22 × 10^−12^ m	5‐HT, Alb, LYZ, Glu, Epn	Serum	^[^ ^270^ ^]^
	HG–DNAzyme/T‐Apt/ SiO_2_@GO@CF	CL	25 × 10^−12^ m–15 × 10^−15^ m	6.3 × 10^−15^ m	BSA, HGB, LYZ, AJP, ALT, Gly, Cys, K^+^, Cl^−^	Serum	^[^ ^266^ ^]^
	CS@Fe_3_O_4_@GO@T‐Apt@HM	CL	0.25 × 10^−9^ m–5 × 10^−15^ m	1.5 × 10^−15^ m	HGB, GF, BSA, AA, GLU, GPT, Tyr, Cys, K^+^, Na^+^, Cl^−^	Serum	^[^ ^271^ ^]^
cTnI	Co^2+^/*N*‐(aminobutyl)‐*N*‐(ethylisoluminol)–Fe_3_O_4_@void @C‐CS/Au–Ab	CL	1 pg mL^−1^– 10 ng mL^−1^	0.50 pg mL^−1^	MYO, IgG, Copeptin, H‐FABP	Serum	^[^ ^272^ ^]^
	Co(II)–Ab2–luminol–AuNP	CL	0.5 pg mL^−1^– 1 µg mL^−1^	0.30 pg mL^−1^	Copeptin, H‐FABP	Serum	^[^ ^273^ ^]^
CEA	ZnS–CdS@MoS_2_/chitosan/ aptamer|GCE	ECL	0.05–20 ng mL^−1^	0.031 ng mL^−1^	HSA, IgG, IgE	Serum	^[^ ^276^ ^]^
	Fe_3_O_4_@SiO_2_@PS	ECL	0.001–80 ng mL^−1^	0.3 pg mL^−1^	AFP, IgG, BSA	Serum	^[^ ^274^ ^]^
	PDDA–rGO/ZnSe@ZnS QDs	ECL	0.1 pg mL^−1^– 100 ng mL^−1^	0.029 pg mL^−1^	AFP, NSE, IgG, BSA	Serum	^[^ ^267^ ^]^
	Ru@SiO_2_–cDNA–Fe_3_O_4_@Au composites	ECL	10 fg mL^−1^– 10 ng mL^−1^	3.5 fg mL^−1^	AFP, BSA, p24, Thrombin	Serum	^[^ ^275^ ^]^
	GR‐IL/pPt composite	ECL	0.001 fg mL^−1^– 1 ng mL^−1^	0.0003 fg mL^−1^	Vc, HSA, Glu, AFP, PSA, IgG, thrombin,	Serum	^[^ ^277^ ^]^
cTnI	Au nanorods–thiolated IgG–anti‐cTnI biochip, NaOL‐coated Fe_3_O_4_ MNPs	SPR	2.5–30 ng mL^−1^	15 ng mL^−1^	–	–	^[^ ^285^ ^]^
	AuNPs–PDA–cAb/Au film sensing platform, Fe_3_O_4_@PDA–dAb immune probe	SPR	15–2500 ng mL^−1^	3.75 ng mL^−1^	Mouse IgG, Bovine IgG	Serum	^[^ ^201^ ^]^
	Hollow AuNPs–PDA–cAB/Au film sensing platform, Fe_3_O_4_–MWCNTs–PDA/dAb immune probe	SPR	15–2500 ng mL^−1^	1.25 ng mL^−1^	Mouse IgG, Bovine IgG	Serum	^[^ ^283^ ^]^
	Fe_3_O_4_ MNPs–cTnI conjugates, Au nanorods–MUDA–anti‐cTnI bioprobe	SPR	0.05 × 10^−9^–10 × 10^−9^ m	30 × 10^−12^ m	Fibrinogen, serum albumin	Blood plasma	^[^ ^284^ ^]^
	MUDA–GNRs and PSS–CTAB–GNRs/anti‐cTnI	SPR	1–20 ng mL^−1^	1 ng mL^−1^	Fibrinogen, plasma, serum albumin, MYO	–	^[^ ^287^ ^]^
	TP–CALNN–AuNPs	LRSPR	10–1000 ng mL^−1^	0.2 ng mL^−1^	–	–	^[^ ^286^ ^]^
AFP	Antigen/BSA/Ab/Au–ZnO flower‐rods	SPR	0.005–50 ng mL^−1^	0.56 pg mL^−1^	CEA, DA	–	^[^ ^288^ ^]^
Myoglobin	Ag nano pine tree|ITO	SERS	10 ng mL^−1^– 5 µg mL^−1^	10 ng mL^−1^	–	Urine	^[^ ^290^ ^]^
	AuNPs|canonical anodic Al_2_O_3_ nanotip arrays	SERS	0.01–1 ppm	0.01 ppm	–	Serum, urine	^[^ ^291^ ^]^
	Ag^MB^@Au/antiMYO Ab, Ag^NBA^@Au/antiCK‐MB Ab, Ag^R6G^@AuNPs/anti‐cTnI Ab	SERS	0.01–500 ng mL^−1^	4.2 pg mL^−1^	CK‐MB, cTnI	Serum	^[^ ^293^ ^]^
	Ag^NBA^@Au/antiMYO Ab, Ag^NBA^@Au/antiCK‐MB Ab, Ag^NBA^@AuNPs/anti‐cTnI Ab	SERS	0.01–500 ng mL^−1^	3.2 pg mL^−1^	CK‐MB, cTnI	Serum	^[^ ^292^ ^]^
	AuNPs–WS_2_ nanohybrid/antiMYO/aptamer	SERS	10 fg mL^−1^– 0.1 µg mL^−1^	10 fg mL^−1^	BSA, HGB	–	^[^ ^289^ ^]^
cTnI	AuNR@NBT@Au/OPSS–PEG–NHS‐anti‐cTnI	SERS	0.1–100 ng mL^−1^	0.1 ng mL^−1^	–	–	^[^ ^297^ ^]^
	Au^NBA^@Ag–Au/cTnI Ab/BSA	SERS	0–50 ng mL^−1^	0.09 ng mL^−1^	CRP, BNP, MYO, CK‐MB	Serum	^[^ ^296^ ^]^
	AuNPs‐MGITC@Ag‐MGITC/ cTnIAb	SERS	0.01–100 ng mL^−1^	8.9 pg mL^−1^	BSA, IgG, HSA, MYO, CK, CK‐MB	Serum	^[^ ^298^ ^]^
	AgNPs–DTNB–silica/silane–PEG–NHS/cTnI Ab/BSA	SERS	27.4 × 10^−15^ m– 1.76 × 10^−12^ m	12.9 × 10^−15^ m	–	–	^[^ ^294^ ^]^
	Ag–Au nanostars‐DTNB, Ag–Au nanostars‐4‐MBA, Ag–Au nanostars‐NT	SERS	0.1–10 ng mL^−1^	0.76 fg mL^−1^	NT‐ProBNP, NGAL, MMP‐2, MMP‐9, IL‐6	Blood plasma, serum	^[^ ^295^ ^]^
NSE	Au nanostar@MBA@silica, Au nanostar@MGITC@silica	SERS	1.0–75.0 ng mL^−1^	0.86 ng mL^−1^	IgG	blood plasma	^[^ ^299^ ^]^
TNF‐*α*	AuNPs@MBA@silica, AuNPs@DTNB@silica, AuNPs@TFMBA@silica	SERS	1 pg mL^−1^– 10 ng mL^−1^	1 pg mL^−1^	BSA, IL‐1, IL‐8	–	^[^ ^300^ ^]^

CZE – capillary zone electrophoresis; ECL – electrochemiluminescence; CL – chemiluminescence; Fluorescence – fluorescence spectroscopy; SPR – surface Plasmon resonance; SERS – surface‐enhanced Raman spectroscopy; LRSPR – long‐range surface plasmon resonance; SP‐LS – surface‐plasmon‐resonance‐enhanced light scattering; MQBs – magnetic‐quantum dot nanobeads; NBA – Nile blue A dye; MB – methylene Blue; R6G – Rhodamine 6 G; NBT – 1,4‐nitrobenzenthiole; MGITC – malachite green isothiocyanate; DTNB – 5,5′‐dithiobis(2‐nitrobenzoic acid); 4‐MBA – 4‐mercaptobenzoic acid; NT – 2‐naphthalenethiol; GERTs – gap‐enhanced Raman tags; LYZ – lysozyme; Glu – glucose; GOD – glucose oxidase; *β*‐LAG – *β*‐lactoglobulin; HGB – hemoglobin; Alb – serum albumin; BSA – bovine serum albumin; HSA – human serum albumin; IgG – human immunoglobulin G; Cys – cysteine, AA – ascorbic Acid; UA – uric acid; DA – dopamine; Epn – epinephrine; 5‐HT – 5‐hydroxytryptamine; ALT – alanine aminotransferase; AJP – alkaline phosphatase; Tyr – tyrosine; GF – growth factor; GPT – glutamate pyruvate transaminase; H‐FABP – heart‐type fatty acid‐binding protein; PSS – polysodium 4‐styrenesulfonate; TP – CALNN‐Peg4‐FYSHSFHENWPS; CALNN – peptide spacer; TP–CALNN–AuNPs; CEA – carcinoembryonic antigen; AFP – *α*‐fetoprotein; PSA – prostate specific antigen; NSE – neuron‐specific enolase; CA 125 – carbohydrate antigen 125; CA15‐3 – carbohydrate antigen 15‐3; CA‐199 – carbohydrate antigen 199; MYO – myoglobin; CK – creatine kinase; NT‐ProBNP – *N*‐terminal prohormone of brain natriuretic peptide; NGAL – neutrophil gelatinase‐associated lipocalin

Though these techniques offer very good results, the major drawbacks are tedious procedures, extensive time consumption, and the sophisticated expensive instrumentation, which needs utmost care by qualified and well‐trained technicians. As an alternative to the above‐mentioned transduction methods, electrochemical techniques offer facile sampling, analysis, detection, and readout of biological binding data in a self‐contained portable system.

## 4 Electrochemical Sensors Based on the Hybrid Metallic Composite Nanostructures

Electroanalytical techniques (amperometry, cyclic, differential pulse, square wave voltammetry, and electrochemical impedance spectroscopy) were considered as the latest and novice friendly transduction methods, which offer portable instrumentation. Electrochemical sensors can be developed for any electroactive molecules, whereas impedance‐based sensors can be developed even for the nonelectroactive molecules. Electrochemical sensor provides the accurate and reliable information of the chemical composition of the materials involved. It also gives the reversible uninterrupted response without agitating the sample. It deals with the interrelationship between current/potential and the chemical composition of the components. Interaction of target analyte with the recognition matrix leads to the variation in the electrical signal.

Amperometric sensors have the advantages that they can operate at a fixed redox potential, provide a signal, which varies linearly with the concentration of the analyte, fast response time, high reproducibility, and high sensitivity. In addition, amperometric sensors are compact and can be used for the continuous monitoring. For these reasons, existing portable sensors were majorly constructed using amperometry (Amp) technique.

Cyclic voltammetry (CV) helps us to understand the basic redox characteristics of the analyte or recognition matrix. It can be used to monitor the variations in the composition of recognition matrix. Differential pulse voltammetry (DPV) and square wave voltammetry (SWV) give the readable signal response to quantify the selected analytes of sub‐picomolar concentrations. Hence, the reported limit of detection (LOD) values is in the range of sub‐pico‐ to femtomolar levels.

Electrochemical impedance spectroscopy (EIS) is able to measure resistive as well as capacitive properties at the electrode–electrolyte interface. EIS works by applying a sine wave with low amplitude to perturb the equilibrium. EIS is a nondestructive and robust technique, which gives the critical information about adsorption/desorption at the electrode surface. EIS not only helps in understanding the kinetic/mechanistic information but also helps in calculating the reaction rates at the electrode surface. Several biosensors have been constructed by applying the core concepts of EIS in combination with biological recognition elements. The use of affinity‐based biosensors in EIS makes it convenient for the direct and label‐free electrochemical immunosensing and potentially speeding up the analysis.

The following sections meticulously discuss various electrochemical sensor systems examined toward the detection of crucial biomedical markers predominantly the recognition matrices which contain hybrid metallic composite nanostructures.

### 4.1 Cancer Biomarkers

#### 4.1.1 Detection of PSA

**Figure 4:**
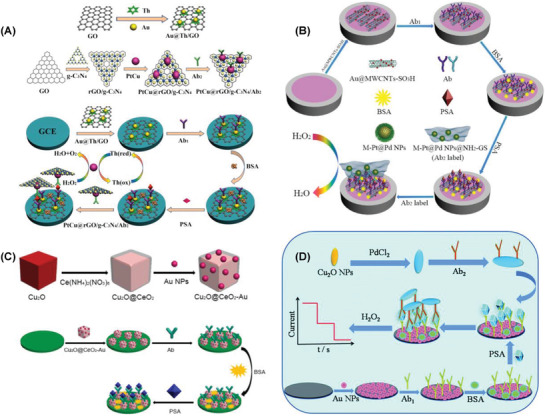
Strategies applied for the electrochemical detection of PSA using A) PtCu@rGO/g‐C_3_N_4_| nanoAu–GCE. Reproduced with permission.^[^ ^301^ ^]^ Copyright 2016, Elsevier. B) M‐Pd@Pt/NH_2_‐GS|GCE. Reproduced with permission.^[^ ^302^ ^]^ Copyright 2016, Elsevier. C) Cu_2_O@CeO_2_–Au|GCE. Reproduced with permission.^[^ ^303^ ^]^ Copyright 2016, Elsevier. D) Pd@Cu_2_O NPs|Au–NH_2_–GCE. Reproduced with permission.^[^ ^304^ ^]^ Copyright 2016, Royal Society of Chemistry.

The combination of bimetallic nanoparticles with highly conductive graphene derivatives was investigated toward the detection of PSA. An immunosensor was designed based on the PtCu bimetallic hybrid synthesized by a facile hydrothermal method (**Figure** **4**A). PtCu were stacked on 2D/2D‐reduced graphene oxide/graphitic carbon nitride along with an antibody immobilized on glassy carbon electrode attached with gold nanoparticles (PtCu@rGO/g‐C_3_N_4_|nano Au–GCE). Fabricated immunosensor exhibited a low detection limit of 16.6 fg mL^−1^ in the linear concentration range of 50 fg mL^−1^–40 ng mL^−1^ PSA.^[^ ^301^ ^]^

In another report, bimetallic nanoparticles loaded on functionalized graphene were investigated for the detection of PSA (Figure 4B). Core–shell Pd@Pt nanoparticles were synthesized in the initial step and then stacked on to the amino group functionalized graphene. Resultant nanocomposite along with a suitable aptamer was deposited on GCE (M‐Pd@Pt/NH_2_‐GS|GCE). The proposed sensor worked efficiently in the linear concentration range of 10 fg mL^−1^–50 ng mL^−1^ and the detection limit of PSA was reported as 3.3 fg mL^−1^.^[^ ^302^ ^]^ Better selectivity was achieved with the presence of relevant aptamer.

Voltammetric detection of PSA was reported using bimetallic dendritic core–shell AuPd@Au nanocrystals. One‐pot synthetic method was used to prepare AuPd@Au NCs. These nanocomposites in combination with suitable antibody were deposited on GCE (PSA/BSA/Ab/AuPd@Au NCs|GCE). Electrocatalytic reduction of H_2_O_2_ was used for quantifying the amount of PSA. Biocompatibility, superior catalytic activity of AuPd@Au NCs led to a highly sensitive PSA immunosensor. The proposed DPV sensor offered an LOD of 0.078 ng mL^−1^ in the concentration range of 0.1–50 ng mL^−1^.^[^ ^305^ ^]^ An interesting electrochemical immunosensor was proposed using trimetallic nanospheres of PdPtCu and AuNPs for quantifying the ultratrace levels of PSA. GCE was modified with the nanocomposites and suitable antibody to fabricate the m‐PdPtCu/AuNPs/Ab1/BSA/PSA/Ab2|GCE. DPV analysis of the sensor system offered an LOD value of 3.3 fg mL^−1^ in the concentration range of 10 fg mL^−1^–100 ng mL^−1^.^[^ ^306^ ^]^

Bimetallic oxide derivatives were synthesized and evaluated their sensor characteristics toward the detection of PSA. GCE was used as the platform for fabricating the immunosensor composed of Cu_2_O@CeO_2_–Au nanocomposite, suitable antibody, and BSA (PSA/BSA/Ab/Cu_2_O@CeO_2_–Au|GCE). The resultant immunosensor exhibited better electrocatalytic activity toward the reduction of hydrogen peroxide because of the synergetic effect of nanocomposite (Figure 4C). The amperometric immunosensor worked well in a wide linear range of 0.1 pg mL^−1^–100 ng mL^−1^ with a low detection limit of 0.03 pg mL^−1^.^[^ ^303^ ^]^ Metal‐doped bimetallic oxide derivatives were examined for the detection of PSA (Figure 4D). Amperometric quantification of PSA was reported using palladium‐doped cuprous oxide nanoparticles Pd@Cu_2_O NPs deposited on a GCE surface modified with Au‐NH_2_ nanoparticles (Pd@Cu_2_O NPs/Au|NH_2_‐GCE). The nanocomposite was further coated with an antibody and BSA to fabricate the immunosensor. Chronoamperometric analysis of PSA in the concentration range of 10 fg mL^−1^–100 ng mL^−1^ exhibited a low detection limit of 2 fg mL^−1^.^[^ ^304^ ^]^

Electrochemical analysis of PSA was reported using a novel sandwich‐type immunosensor. A hybrid nanocomposite of Au@Ag–Cu_2_O was used considering the synergism among Au, Ag, and Cu_2_O. The nanocomposite has exhibited excellent electrocatalytic activity along with good biocompatibility and high specific surface area. AuNPs‐functionalized nitrogen‐doped graphene quantum dots Au@N‐GQDs have also been used. The recognition matrix was deposited on GCE (Au@Ag–Cu_2_O/Ab2/PSA/BSA/Ab1/Au@N‐GQDs|GCE). Amperometric immunosensor of PSA offered an LOD of 0.003 pg mL^−1^ in the range of 0.01 pg mL^−1^–100 ng mL^−1^.^[^ ^307^ ^]^ Synergistic effect of metallic sulfides, oxides, and ionic liquid derivatives were examined toward the selective detection of PSA. A nanocomposite of Au–CoS/graphene and CeO_2_/ionic liquids doped with carboxymethyl chitosan complex was mixed with antibody and further coated on GCE (Au–CoS/graphene–CeO_2_/IL‐carboxymethyl chitosan|GCE). The resultant immunosensor displayed good voltammetric response with a low detection limit of 0.16 pg mL^−1^ in the linear concentration range of 0.5 pg mL^−1^–50 ng mL^−1^.^[^ ^308^ ^]^

Label‐free detection of PSA was reported using Cu_7_S_4_ nanocrystals, which were synthesized by a facile solvothermal method. Immunosensor was fabricated by using the relevant aptamer and BSA on GCE surface (BSA/anti‐PSA/Cu_7_S_4_ nanocrystal|GCE). Better electrocatalytic activity was observed with the Cu_7_S_4_ nanocrystals compared to CuS spheres and CuS flowers. The calculated LOD value was 0.001 ng mL^−1^ in the concentration range of 0.001–15 ng mL^−1^. The fabricated sensor displayed good sensitivity, selectivity, and stability toward the detection of PSA.^[^ ^13^ ^]^ Impedimetric sensor for the quantification of PSA was reported with the aid of a hybrid composite of Au nanoshells with a magnetic core MP@silica@Au. The hybrid nanocomposite was deposited on gold electrode (MP@silica@Au/CB/Ab|gold). The fabricated EIS sensor could detect PSA as low as 34 fg mL^−1^ in the concentration range of 0.01–1 pg mL^−1^.^[^ ^309^ ^]^

A dual functional aptasensor was reported using a hybrid nanocomposite of MoS_2_ QDs@g‐C_3_N_4_@CS–AuNPs deposited on gold electrodes (MoS_2_ QDs@g‐C_3_N_4_@CS–AuNPs|gold). SPR spectroscopy sensor and impedimetric aptasensors were fabricated with the hybrid nanocomposite. The fabricated EIS sensor offered an LOD value of 0.71 pg mL^−1^ whereas the SPR sensor offered 0.77 ng mL^−1^ in the concentration range of 1–250 ng mL^−1^.^[^ ^310^ ^]^ A robust electrochemical PSA sensor was reported using a hybrid nanocomposite of multifunctional hydroxyl pillar[5]arene@AuNP@g‐C_3_N_4_ combined with a suitable antibody deposited on GCE (HP5@AuNPs@g‐C_3_N_4_|GCE). The as‐prepared DPV‐based immunosensor displayed an LOD value of 0.12 pg mL^−1^ in the linear concentration range of 0.5 pg mL^−1^–10 ng mL^−1^. The proposed sensor possessed many advantages such as low cost, simple preparation and fast detection with a remarkable robustness, ultra‐sensitivity, excellent selectivity, and reproducibility.^[^ ^311^ ^]^

Metal oxide nanoparticles in combination with conducting CNT and imprinted polymers were examined toward the detection of PSA. CNT/MnO_2_ hybrids were first synthesized by a facile microwave irradiation method. Further, MnO_2_‐modified MWCNTs‐MIP matrix was prepared on pencil graphite electrode (CNT/MnO_2_‐MIP|PGE). Voltammetric analysis (DPV and SWV) of PSA was reported with a low detection of 3.04 fg mL^−1^ in the range of 0.01–10 pg mL^−1^ and 20–62 ng mL^−1^.^[^ ^312^ ^]^ A voltammetric aptasensor was described for the detection of PSA using hemin‐functionalized graphene–Pd NP nanocomposite combined with PSA aptamer which were deposited on GCE (PSAa‐SA‐DNA‐biotin/H‐Gr/PdNP|GCE). Synergism among the conductivity of graphene, catalytic property of Pd NPs, and selectivity attained by the PSA aptamer led to a highly sensitive electrochemical aptasensor. The resulting aptasensor has an LOD value of 8 pg mL^−1^ in the linear concentration range of 0.025–205 ng mL^−1^ PSA.^[^ ^313^ ^]^

Linear determination ranges and LODs of different methodologies accompanied by the relevant modification materials for the detection of PSA have been compiled in **Table** **2**. The results show that modification materials specifically comprising metallic oxides and sulfides, such as MoS_2_, CeO_2_, Cu_2_O, and CoS, and bimetallic nanoparticles had resulted superior low detection limits. The electrocatalytic currents for the determination of the analyte, PSA, were enhanced enormously with the presence of metallic oxides, sulfides and bimetallic nanoparticles leading to the LOD values as low as 0.16 pg mL^−1^ to 2 fg mL^−1^, despite of various transduction methods, DPV, SWV, EIS, and amperometry.

**Table 2:** Summary of the electrochemical biosensors reported for the detection of cancer biomarkers (PSA, CEA, AFP, NSE, and ferritin)

Biomarker	Recognition matrix|electrode	Method	Concentration range	LOD	Interferents	Real samples	Ref.
PSA	PSA/BSA/Ab/ AuPd@AuNCs|GCE	Amp	0.1–50 ng mL^−1^	0.078 ng mL^−1^	AA, BSA, CA125, CA153	Serum	^[^ ^305^ ^]^
	BSA/anti‐PSA/Cu_7_S_4_ nanocrystal|GCE	Amp	0.001–15 ng mL^−1^	0.001 ng mL^−1^	AFP, IgG, BSA	Serum	^[^ ^13^ ^]^
	PSAa–SA–DNA–biotin/H‐Gr/PdNP|GCE	DPV	0.025–205 ng mL^−1^	8 pg mL^−1^	Thrombin, IgG, HSA, CEA	Serum	^[^ ^313^ ^]^
	MoS_2_ QDs@g‐C_3_N_4_@CS‐AuNPs|gold	EIS	1–250 ng mL^−1^	0.71 pg mL^−1^	CEA, IgG, glucose	Serum	^[^ ^310^ ^]^
	Au–CoS/graphene, CeO_2_/IL‐carboxymethyl chitosan|GCE	DPV	0.5 pg mL^−1^– 50 ng mL^−1^	0.16 pg mL^−1^	CEA, AFP, AA, BSA	Serum	^[^ ^308^ ^]^
	HP5@AuNPs@g‐C_3_N_4_|GCE	DPV	0.5 pg mL^−1^–10 ng mL^−1^	0.12 pg mL^−1^	BSA, AA, GSH, CEA, AFP	Serum	^[^ ^311^ ^]^
	Cu_2_O@CeO_2_–Au|GCE	CA	0.1 pg mL^−1^–100 ng mL^−1^	0.03 pg mL^−1^	IgG, CEA, AFP	Serum	^[^ ^303^ ^]^
	Au@Ag–Cu_2_O/Ab2/PSA/BSA/ Ab1/Au@N‐GQDs|GCE	Amp	0.01 pg mL^−1^–100 ng mL^−1^	0.003 pg mL^−1^	HBS, CEA, IgG, BSA	Serum	^[^ ^307^ ^]^
	MP@silica@Au/CB/Ab|gold	EIS	0.01–1 pg mL^−1^	34 fg mL^−1^	–	Serum	^[^ ^309^ ^]^
	PtCu@rGO/g‐C_3_N_4_|nanoAu–GCE	Amp	50 fg mL^−1^–40 ng mL^−1^	16.6 fg mL^−1^	CEA, IgG, BSA, HBS, Glucose	Serum	^[^ ^301^ ^]^
	m‐Pd@Pt/NH_2_‐GS|GCE	Amp	10 fg mL^−1^–50 ng mL^−1^	3.3 fg mL^−1^	IgG, CEA, HBS, BSA	Serum	^[^ ^302^ ^]^
	m‐PdPtCu/AuNPs/Ab1/BSA/ PSA/Ab2|GCE	DPV	10 fg mL^−1^–100 ng mL^−1^	3.3 fg mL^−1^	IgG, CEA, HBe, HBS, BSA	Serum	^[^ ^306^ ^]^
	CNT/MnO_2_ hybrids |PGE	DPSV SWSV	0.01–10 pg mL^−1^ 20–62 ng mL^−1^	3.04 fg mL^−1^	Citric acid, Tyr, Arg, GA, His, Lys, Alb, Glo, UA, ferritin, urea, Ins, NaCl	Urine, serum	^[^ ^312^ ^]^
	Pd@Cu_2_O NPs|Au–NH_2_–GCE	CA	10 fg mL^−1^–100 ng mL^−1^	2 fg mL^−1^	BSA, CEA, SCCA, AFP	Serum	^[^ ^304^ ^]^
CEA	AgNPs‐rGO|SPE	CV	0.05–0.50 µg mL^−1^	35 ng mL^−1^	–	–	^[^ ^319^ ^]^
	Ab2–AuNPs–Fc|gold	SWV	0.05–20 ng mL^−1^	0.01 ng mL^−1^	BSA, Glu, Cys, IgG, PSA	Serum	^[^ ^318^ ^]^
	Au–Cu_2_S–CuS/graphene	DPV	0.1 pg mL^−1^–100 ng mL^−1^	0.78 pg mL^−1^	AA, BSA, AFP, PSA	Serum	^[^ ^314^ ^]^
	MoS_2_–PBNCs|GCE	DPV	0.5 pg mL^−1^–10 ng mL^−1^	0.54 pg mL^−1^	BSA, IgG, AFP, NSE	Serum	^[^ ^315^ ^]^
	Few‐layer black phosphorus–AuNP|GCE	EIS	1 pg mL^−1^ – 10 µg mL^−1^	0.20 pg mL^−1^	BSA, AFP, PSA	Serum	^[^ ^321^ ^]^
	Fe_3_O_4_@MnO_2_@Pt–Ab2|GCE	Amp	0.5 pg mL^−1^–20 ng mL^−1^	0.16 pg mL^−1^	BSA, Vitamin C, Glu, AFP, CA125	Serum	^[^ ^317^ ^]^
	AuNPs/CNOs/SWCNTs/CS|GCE	SWV	100 fg mL^−1^–400 ng mL^−1^	100 fg mL^−1^	BSA, AA, AFP, UA, DHEA, CRP, Leptin, Cortisol, hCG, Haptoglobin	Serum	^[^ ^207^ ^]^
	GS‐Fe_3_O_4_/Au@Ag/Ni^2+^|GCE	Amp	0.1 pg mL^−1^–100 ng mL^−1^	69.7 fg mL^−1^	BSA, IgG, AFP, TSH	Serum	^[^ ^316^ ^]^
	Ab/polyCBMA/PANI|GCE	DPV	10 fg mL^−1^–100 pg mL^−1^	3.05 fg mL^−1^	BSA, PSA, AFP, HSA, ssDNA	Serum	^[^ ^320^ ^]^
	Ag/MoS_2_/rGO|GCE	CA	0.01 pg mL^−1^–100 ng mL^−1^	1.6 fg mL^−1^	AFP, PSA, IgG, BSA	Serum	^[^ ^204^ ^]^
AFP	AFP–aptamer/TH/RGO/AuNPs |SPE	DPV	0.1–100.0 µg mL^−1^	0.050 µg mL^−1^	BSA, HSA, IgG, IgE	Serum	^[^ ^197^ ^]^
	Fe_3_O_4_@Au‐Ab1|GCE	Amp	20–100 ng mL^−1^	0.64 ng mL^−1^	BSA, CEA, IgG	Serum	^[^ ^202^ ^]^
	BSA/Ab/CS/IrO*_x_*|FTO	CV	1–250 ng mL^−1^	0.3 ng mL^−1^	AA, CEA, GLU, PSA	Serum	^[^ ^325^ ^]^
	Hep‐PGA‐PPy NPs|GCE	DPV	0.1–100 ng mL^−1^	0.099 ng mL^−1^	AA, BSA, UA, CEA, Glu	Whole blood	^[^ ^324^ ^]^
	N–GS–AuNP–Chit–anti‐AFP–BSA|GCE	DPV	5 pg mL^−1^–50 ng mL^−1^	1.6 pg mL^−1^	AA, PSA, Glu, BSA	Serum	^[^ ^323^ ^]^
	Pt NPs/Co_3_O_4_/graphene|GCE	Amp	0.1 pg mL^−1^–60 ng mL^−1^	0.029 pg mL^−1^	Glu, PSA, IgG, CEA, Vitamin C	Serum	^[^ ^322^ ^]^
	NanoAu–MoS_2_–Ab1‐BSA|GCE	DPV	50 fg mL^−1^–75 ng mL^−1^	2.0 fg mL^−1^	IgG, CEA, CRP, PSA	Serum	^[^ ^205^ ^]^
	MO/CNT–Au/Ab2–Ag–Ab1–AuPt‐VG|GCE	DPV	1 fg mL^−1^–100 ng mL^−1^	0.7 fg mL^−1^	CEA, CA‐125, CA‐199, CA‐153	Serum	^[^ ^206^ ^]^
NSE	NH_2_‐G/Thi/AuNPs|*μ*PADs	DPV	1–500 ng mL^−1^	10 pg mL^−1^	AA, DA, UA, LH, 5‐HT, CFP10, CEA	Serum	^[^ ^329^ ^]^
	MIP‐Poly(DPIMBr)/GNA‐MVIMBF4|GCE	DPV	0.01–1.0 ng mL^−1^	2.6 pg mL^−1^	HSA, IgG, HGB, AA, Gly, Cys, His	Serum	^[^ ^331^ ^]^
	Polymer‐Au/Pd‐SA‐AuNP|SPCE	SWV	0.01–200 ng mL^−1^	2.3 pg mL^−1^	UA, AA, IgG, AFP	Serum	^[^ ^330^ ^]^
	Polypyrrole–polythionine–NanoAu–Ab1–BSA|GCE	Amp	100 ng mL^−1^–1 pg mL^−1^	0.65 pg mL^−1^	UA, AA, glucose, CEA, AFP, PSA	Serum	^[^ ^327^ ^]^
	AuPd–MWCNT/CS–Fc |GCE	SWV	1 pg mL^−1^–100 ng mL^−1^	0.483 pg mL^−1^	AA, PSA, SCCA, IgG, CA125, CK19	Serum	^[^ ^332^ ^]^
	3DM rGO/PANI–Ab1–BSA|3D silica–gold	DPV	0.5 pg mL^−1^–10 ng mL^−1^	0.1 pg mL^−1^	AFP, BSA, CA724, CEA	Serum	^[^ ^326^ ^]^
	TB/WP6@PdPt‐Ab2	DPV	0.3 pg mL^−1^–100 ng mL^−1^	0.095 pg mL^−1^	BSA, PSA, AFP, CEA and SCCA	Serum	^[^ ^333^ ^]^
	MCH/DNA–SH/AuNP |GCE	SWV	0.0001–100 ng mL^−1^	52.14 fg mL^−1^	NSE, IgG, CEA, PSA, CA199, CA242	Serum	^[^ ^334^ ^]^
	H‐rGO–Thi–Au–Ab1–BSA|GCE	SWV	0.1 pg mL^−1^–100 ng mL^−1^	26 fg mL^−1^	AA, UA, DA, IgG, Glu, BSA, PSA, CEA, HSA	Serum	^[^ ^328^ ^]^
	BSA/NSE/Ab1/CS/3D‐GNS|GCE	ASV	0.02 pg mL^−1^–35 ng mL^−1^	8 fg mL^−1^	AFP, CEA, hCG	Serum	^[^ ^335^ ^]^
	P(Pyr‐Epx)/anti‐NSE/BSA|ITO	EIS	0.02–7.5 pg mL^−1^	6.1 fg mL^−1^	CDH 22, TNF‐*α*, RACK1, SOX 2	Serum	^[^ ^336^ ^]^
	BSA/anti‐NSE /P(Thi‐g‐MAm) |ITO	EIS	0.02–4 pg mL^−1^	6.1 fg mL^−1^	IL‐1*α*, IL‐1*β*, TNF *α*, VEGF, p53, CDH22	Serum	^[^ ^208^ ^]^
Ferritin	Ab/‐C_6_H_4_R/Fe@C NPs|Gold R = carboxyphenyl R = aminoethylophenyl	EIS DPV EIS DPV	0.1–30 µg dL^−1^ 0.01–20 µg dL^−1^	0.40 µg dL^−1^ 0.13^ ^µg dL^−1^ 0.03 µg dL^−1^ 0.02 µg dL^−1^	HGB, transferrin	Water, blood plasma	^[^ ^338^ ^]^
	Antiferritin–agarose hydrogel|GCE	DPV	50–500 ng mL^−1^	15 ng mL^−1^	–	Serum	^[^ ^342^ ^]^
	Au nanorods–GNR–Ab1|cotton Thread	ASV	5–5000 ng mL^−1^	1.58 ng mL^−1^	IgG, CEA, SCCA	Serum	^[^ ^337^ ^]^
	Cu_2_O–SiO_2_–Ab1–BSA|gold	DPV	1.0–5.0, 5.0–120.0 ng mL^−1^	0.4 ng mL^−1^	–	Serum	^[^ ^339^ ^]^
	NanoAu–Chit–Ab1–BSA|GCE	Potentiometry	1–500 ng mL^−1^	0.1 ng mL^−1^	AFP, BSA, CA 125, CA 19‐9	Serum	^[^ ^341^ ^]^
	Anti‐Ft/thionine/BSA/GOD‐Fe_3_0_4_–SiO_2_|SPCE	CV	0.1–400 ng mL^−1^	10 pg mL^−1^	–	Serum	^[^ ^340^ ^]^

Amp – amperometry; CA – chronoamperometry; SWSV – square wave stripping voltammetry; DPSV – differential pulse stripping voltammetry; ASV – anodic stripping voltammetric analysis; HBS – hepatitis B surface antigen; HBe – hepatitis B e antigen; CEA – carcinoembryonic antigen; AFP – *α*‐fetoprotein; SCCA – squamous cell carcinoma antigen; PSA – prostate specific antigen; NSE – neuron‐specific enolase; CA 125 – carbohydrate antigen 125; CA15‐3 – carbohydrate antigen 15‐3; CA‐199 – carbohydrate antigen 199; CA724 – carbohydrate antigen 724; CDH 22 – cadherin‐like protein 22; RACK 1 – receptor activated kinase 1; IL 8 – interleukin 8; Glo – globulin; IgG – immunoglobulin G; IgE – immunoglobulin E; Alb – albumin; HSA – human serum albumin; BSA – bovine serum albumin; HGB – hemoglobin; GSH – l‐glutathione; Tyr – tyrosine; Arg – arginine; GA – glutamic acid; His – histidine; Gly – glycine; Lys – lysine; Cys – cysteine; Ins – insulin; Glu – glucose; AA – ascorbic acid; UA – uric acid; DA – dopamine; 5‐HT – 5‐hydroxytryptamine; fer – ferritin; DHEA – dehydroepiandrosterone; hCG – human chorionic gonadotropin; TSH – thyroid stimulating hormone; ssDNA – single stranded DNA; CFP10 – culture filtrate protein 10; LH – luteinizing hormone;

#### 4.1.2 Selective Quantification of CEA

**Figure 5:**
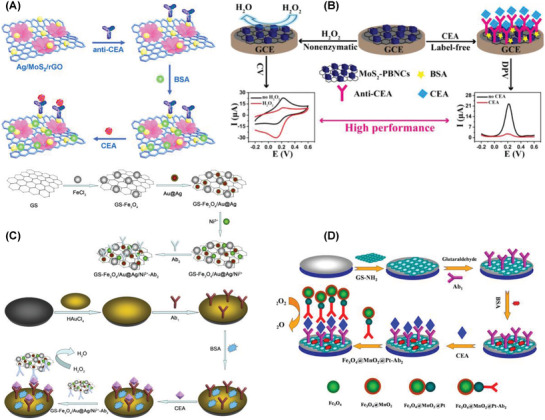
Fabrication of electrochemical sensors for the detection of CEA with the aid of A) Ag/MoS_2_/rGO|GCE. Reproduced with permission.^[^ ^204^ ^]^ Copyright 2018, Elsevier. B) MoS_2_–PBNCs–antiCEA|GCE. Reproduced with permission.^[^ ^315^ ^]^ Copyright 2017, American Chemical Society. C) Fe_3_O_4_/Au@Ag/Ni^2+^|GS. Reproduced with permission. ^[^ ^316^ ^]^ Copyright 2017, Elsevier. D) Fe_3_O_4_@MnO_2_‐Pt‐Ab_2_|GCE. Reproduced with permission.^[^ ^317^ ^]^ Copyright 2015, American Chemical Society.

Bimetallic sulfide derivatives in combination with gold nanoparticles were investigated for the selective detection of CEA. The immunosensor was constructed with the nanocomposite of gold nanoparticles, Cu_2_S–CuS and graphene as matrix material, which was further deposited on screen printed carbon electrodes (Au–Cu_2_S–CuS/graphene|SPCE). Au–CeO_2_ supported TB complex along with carboxymethyl chitosan‐doped ionic liquid and a suitable antibody (TB/Au–CeO_2_/CMC/ILs‐Ab2) was used as the signal label. DPV analysis of CEA offered a detection limit of 0.78 pg mL^−1^ in the linear concentration range of 0.1 pg mL^−1^–100^ ^ng mL^−1^.^[^ ^314^ ^]^ Due to the synergistic effect between molybdenum disulfide (MoS_2_), rGO, and Ag NPs, the resultant nanocomposite Ag/MoS_2_/rGO offered very good sensor characteristics toward the selective detection of CEA (**Figure** **5**A). The resultant nanocomposite along with anti‐CEA and BSA was immobilized on freshly polished GCE to prepare the immunosensor (Ag/MoS_2_/rGO|GCE). Chronoamperometric quantification of CEA at the constructed immunosensor displayed a low detection limit of 1.6 fg mL^−1^ in the linear concentration range of 0.01 pg mL^−1^–100 ng mL^−1^.^[^ ^204^ ^]^

Effect of controlled structural morphology on the sensor performance was investigated toward the detection of CEA (Figure 5B). Shape controlled Prussian blue nanocube‐decorated molybdenum disulfide (MoS_2_‐PBNCs) nanocomposite was synthesized by a facile microwave irradiation. An immunosensor was fabricated by immobilizing the above nanocomposite and anti‐CEA on a GCE surface (MoS_2_‐PBNCs‐anti‐CEA|GCE). The reported DPV sensor worked very efficiently in the selective detection of CEA and displayed a low detection limit of 0.54 pg mL^−1^ in the linear concentration range of 0.5 pg mL^−1^–10 ng mL^−1^.^[^ ^315^ ^]^

A hybrid nanocomposite was fabricated using gold and silver core–shell nanoparticles with nickel ion immobilized on amino functionalized magnetic graphene (Fe_3_O_4_/Au@Ag/Ni^2+^|GS) using electrodeposition (Figure 5C). Sandwich type immunosensor was constructed with the nanocomposite combined with a suitable antibody and BSA deposited on GCE surface. Proposed immunosensor worked selectively toward the amperometric detection of CEA with a low detection limit of 69.7 fg mL^−1^ in the range of 0.1 pg mL^−1^ to 100 ng mL^−1^.^[^ ^316^ ^]^ Mixed metallic oxides in conjunction with Pt nanoparticles were investigated toward the selective detection of CEA. Fe_3_O_4_@MnO_2_ nanoparticles were synthesized using a facile ultrasonication method. In the further step, Pt nanoparticles were incorporated into the above bimetallic oxides by in situ chemical reduction method. Resultant hybrid nanocomposite was mixed with an antibody (Fe_3_O_4_@MnO_2_‐Pt‐Ab_2_|GCE) and used as the signal amplifier for the detection of CEA (Figure 5D). An immunosensor was fabricated using amino‐functionalized graphene and antibody of CEA. Reported amperometric sensor system displayed excellent selectivity with a low detection limit of 0.16 pg mL^−1^ in the linear concentration range of 0.5 pg mL^−1^–20 ng mL^−1^ CEA.^[^ ^317^ ^]^

Electrochemical immunosensor of CEA was reported using a hybrid nanocomposite of AuNPs, carbon nano‐onions, SWCNTs, and chitosan (CS) deposited on GCE (AuNPs/CNOs/SWCNTs/CS|GCE). SWV analysis of CEA offered an LOD of 100 fg mL^−1^ in the range of 100 fg mL^−1^ to 400 ng mL^−1^. The reported immunosensor exhibited very good selectivity and resistant‐to‐interference attributed to the synergistic activity among the individual counterparts of the hybrid nanocomposite.^[^ ^207^ ^]^ A voltammetric immunosensor for the detection of CEA was reported using ferrocene‐labeled biofunctionalized AuNPs deposited on gold electrodes (Ab2‐AuNPs‐Fc|gold). The resultant nanohybrid provided internal electrochemical signals avoiding the necessity of external redox species in the electrolyte. SWV analysis of CEA has offered an LOD value of 0.01 ng mL^−1^ in the concentration range of 0.05–20 ng mL^−1^.^[^ ^318^ ^]^

Ag NPs were mixed with rGO to modify the surface of a screen‐printed carbon electrode (SPE), which further modified to form a sandwich type immunosensor using horseradish peroxidase (HRP)‐conjugated secondary antibody. Resultant immunosensor displayed promising results for the detection of CEA with a low detection limit of 35 ng mL^−1^ in the linear range of 0.05–0.50 µg mL^−1^ whereas the nonsandwich type sensor lead to the detection limit of 42 ng mL^−1^ in the linear range of 0.05–0.40 µg mL^−1^ CEA.^[^ ^319^ ^]^ Voltammetric quantification of CEA was reported using a nanocomposite of zwitterionic poly(carboxybetaine methacrylate) and polyaniline nanowires deposited on GCE surface (Ab/polyCBMA/PANI/GCE). The resultant nanocomposite of biocompatible materials contributed to the fabrication of a low fouling biosensor. DPV analysis of CEA was carried out and the observed LOD value was 3.05 fg mL^−1^ in the concentration range of 10 fg mL^−1^–100 pg mL^−1^. Practical application of the fabricated low fouling, label‐free CEA biosensor has been demonstrated successfully in the undiluted human serum samples.^[^ ^320^ ^]^

Gold nanoparticle/few‐layer black phosphorus (BP‐Au) hybrid nanocomposite was prepared by a facile chemical approach. The hybrid along with anti‐CEA loaded on GCE surface has been investigated toward the label‐free detection of CEA. Reported impedimetric sensor displayed a characteristic low detection limit of 0.20 pg mL^−1^ in a wide detection range of 1 pg mL^−1^–10 µg mL^−1^.^[^ ^321^ ^]^

Electrochemical sensors reported for the detection of CEA have been compiled in Table 2 along with the relevant linear determination ranges and LODs of different methodologies and modification materials. Similar to the observations arrived at for the detection of PSA, the results show that modification materials comprising metallic oxides and sulfides and bimetallic nanoparticles, such as MoS_2_, Fe_3_O_4_, Fe_3_O_4_@MnO_2_, Au@Ag, and Cu_2_S, had shown superior low detection limits. The electrocatalytic currents for the determination of the analyte, CEA, were enhanced enormously with the presence of metallic oxides, sulfides, and bimetallic nanoparticles, and the LOD values were as low as 0.78 pg mL^−1^ to 1.6 fg mL^−1^ with the various transduction methods, DPV, CA, SWV, and EIS.

#### 4.1.3 Electrochemical Analysis of AFP

**Figure 6:**
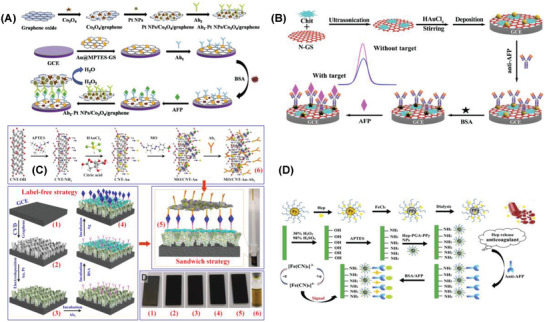
Electrochemical sensor interfaces reported for the detection of AFP using A) Au@MPTES–GS–Ab1–BSA|GCE. Reproduced with permission.^[^ ^322^ ^]^ Copyright 2017, Elsevier. B) N‐GS–AuNP–Chit–anti‐AFP–BSA|GCE. Reproduced with permission.^[^ ^323^ ^]^ Copyright 2017, Springer‐Verlag GmbH Austria. C) MO/CNT‐Au/Ab2‐Ag‐Ab1‐AuPt‐VG|GCE. Reproduced with permission.^[^ ^206^ ^]^ Copyright 2019, Elsevier. D) Hep‐PGA‐PPy NPs|GCE. Reproduced with permission.^[^ ^324^ ^]^ Copyright 2017, Elsevier.

Hybrid nanocomposite constructed with platinum nanoparticles, cobalt oxide, and graphene nanosheets (Pt NPs/Co_3_O_4_/graphene) was examined for the detection of AFP (**Figure** **6**A). Signal probe was constructed using gold nanoparticles adhered on 3‐mercaptopropyltriethoxysilane functionalized graphene sheets (Au@MPTES‐GS). Immunosensor was constructed by immobilizing the above hybrid nanocomposite, an antibody and BSA on GCE surface (Au@MPTES‐GS‐Ab1‐BSA|GCE). Amperometric detection of AFP exhibited a low detection limit of 0.029 pg mL^−1^ in a linear concentration range of 0.1 pg mL^−1^–60 ng mL^−1^.^[^ ^322^ ^]^

A highly selective immunosensor was reported for the detection of AFP. Hybrid nanocomposite was prepared by mixing N‐doped graphene, electrodeposited gold nanoparticles, and chitosan (N‐GS–AuNP–Chit) by temperature‐controlled reaction in a round bottom flask (Figure 6B). Sandwich type immunosensor was developed with the above synthesized nanohybrid, anti‐AFP and BSA immobilized on GCE surface (N‐GS–AuNP–Chit–anti‐AFP–BSA|GCE). Hexacyanoferrate was used as an electrochemical probe. DPV analysis revealed the low detection limit of AFP as 1.6 pg mL^−1^ in the linear analytical range of 5 pg mL^−1^–50 ng mL^−1^.^[^ ^323^ ^]^

Voltammetric quantification of AFP has been demonstrated with label‐free and sandwich‐type electrochemical immunosensors (Figure 6C). The recognition matrix composed of AuPt bimetallic nanoparticles, vertical graphene and a suitable antibody was deposited on a glassy carbon electrode (Ag‐Ab1‐AuPt‐VG|GCE and MO/CNT‐Au/Ab2‐Ag‐Ab1‐AuPt‐VG|GCE). Biocompatibility, conductivity of AuPt nanoparticles and the promoted electron transfer by VG sheets acted synergistically in the construction of a highly sensitive voltammetric immunosensor. Sensitivity of the sandwich‐type sensor was higher than that of the label‐free sensor. The calculated LOD value was 0.7 fg mL^−1^ in the range of 1 fg mL^−1^–100 ng mL^−1^.^[^ ^206^ ^]^

Nanohybrid was constructed with the combination of g‐polyglutamic, polypyrrole, and heparin deposited on the surface of GCE (Hep‐PGA‐PPy NPs|GCE). The hybrid was reported as a best recognition matrix, which improved the anti‐biofouling effect and also displayed better selectivity toward the detection of AFP (Figure 6D). DPV analysis of AFP using the proposed immunosensor displayed a low detection limit of 0.099 ng mL^−1^ in the linear range of 0.1–100 ng mL^−1^.^[^ ^324^ ^]^

Hybrid composite of gold nanoparticles‐hollow molybdenum disulfide with a unique structural morphology was synthesized by a facile hydrothermal approach. Sandwich type immunosensor was fabricated using the above hollow structured hybrid, an antibody, and BSA immobilized on to the pretreated GCE surface (nanoAu‐MoS_2_–Ab1–BSA|GCE). Reported voltammetric immunosensor exhibited an excellent selectivity toward AFP with an ultralow detection limit of 2.0 fg mL^−1^ in a linear concentration range of 50 fg mL^−1^–75 ng mL^−1^.^[^ ^205^ ^]^

A single‐step reduction process was reported to synthesize Fe_3_O_4_@Au composite using Fe_3_O_4_, which was prepared by a facile hydrothermal procedure. A pseudohomogeneous electrochemical immunosensor was developed using the above hybrid composite along with an antibody immobilized on magnetic GCE (Fe_3_O_4_@Au‐Ab1|GCE). Reported sensor for the specific detection of AFP displayed a low detection limit of 0.64 ng mL^−1^ in the range of 20–100 ng mL^−1^.^[^ ^202^ ^]^ A label‐free voltammetric detection of AFP was investigated using a hybrid nanocomposite composed of thionin/reduced graphene oxide/gold nanoparticles and a suitable aptamer deposited on screen‐printed carbon electrode surface (AFP–aptamer/TH/RGO/AuNPs|SPE). Thionin molecule has improved the sensor performance because of the special characteristics namely bridging molecule and electron transfer mediator. DPV analysis of AFP led to the LOD value of 0.050 µg mL^−1^ in the range of 0.1–100.0 µg mL^−1^.^[^ ^197^ ^]^ Label‐free voltammetric quantification of AFP was reported using biofunctionalized 3D ordered macroporous iridium oxides deposited on FTO electrodes (BSA/Ab/CS/IrO*_x_*|FTO). The proposed AFP immunosensor exhibited a very good LOD value of 0.3 ng mL^−1^ in the concentration range of 1–250 ng mL^−1^. Good selectivity toward AFP was demonstrated in the presence of ascorbic acid, CEA, glucose, and PSA.^[^ ^325^ ^]^

Different metallic nanocomposite materials examined for the electrochemical detection of AFP were summarized in Table 2. It was observed that the hybrid nanocomposites which involved AuNPs offered much improved LOD values such as 1.6 pg mL^−1^, 2.0 fg mL^−1^, and 0.7 fg mL^−1^ AFP.

#### 4.1.4 Quantitative Analysis of NSE

**Figure 7:**
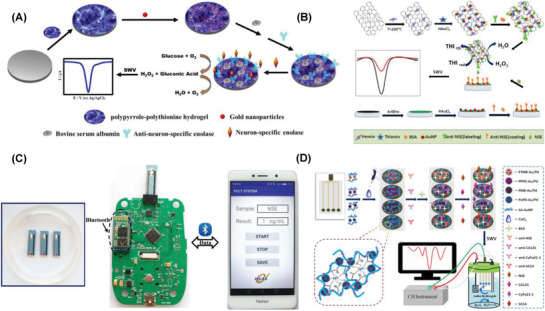
Sensor systems reported for the electrochemical detection of NSE based on A) polypyrrole–polythionine–nanoAu–Ab1–BSA|GCE. Reproduced with permission.^[^ ^327^ ^]^ Copyright 2018, Elsevier. B) H‐rGO–Thi–Au–Ab1–BSA|GCE. Reproduced with permission.^[^ ^328^ ^]^ Copyright 2018, Elsevier. C) NH_2_–G/Thi/AuNPs|*μ*PADs. Reproduced with permission.^[^ ^329^ ^]^ Copyright 2017, Elsevier. D) Polymer–Au/Pd‐SA‐AuNP|SPCE. Reproduced with permission.^[^ ^330^ ^]^ Copyright 2018, Elsevier.

A 3D macroporous reduced graphene oxide/polyaniline deposited on 3D silica‐modified gold electrodes (3DM rGO/PANI‐Ab1‐BSA|3D silica‐gold) based immunosensor has been proposed for the highly selective detection of NSE. DPV quantification of NSE using the proposed immunosensor displayed a low detection limit of 0.1 pg mL^−1^ in the range of 0.5 pg mL^−1^–10 ng mL^−1^. Reported sensor system exhibited a highly selective, highly stable, and a very good reproducible signal response toward NSE.^[^ ^326^ ^]^ A multifunctional conductive hydrogel based amperometric immunosensor was proposed (**Figure** **7**A) using the components pyrrole, thionine, ammonium persulfate, HAuCl_4_, and glucose oxidase (polypyrrole–polythionine–nanoAu–Ab1–BSA|GCE). The fabricated amperometric NSE sensor operated at 0 V (vs Ag|AgCl) exhibited an ultralow detection limit of 0.65 pg mL^−1^ in a wide liner range of 100 ng mL^−1^–1 pg mL^−1^.^[^ ^327^ ^]^ Reduced graphene oxide–thionine–hemin–Au (H‐rGO–Thi–Au) nanohybrid was prepared in a single step and facile microwave irradiation approach (Figure 7B). Immunosensor was constructed using the above hybrid, anti‐NSE, and BSA immobilized on the surface of GCE (H‐rGO–Thi–Au–Ab1–BSA|GCE). The reported immunosensor was highly selective toward the detection of NSE with a low detection limit of 26 fg mL^−1^ in the linear range of 0.1 pg mL^−1^ to 100 ng mL^−1^ using SWV as the method of transduction.^[^ ^328^ ^]^

A wireless point‐of‐care testing system connected to smartphone was proposed for the voltammetric detection of NSE (Figure 7C). Amino functionalized graphene, thionine, and gold nanoparticles (NH_2_‐G/Thi/AuNPs) based hydrogel was synthesized by a simple dispersion and centrifugation approach. *μ*PADs were fabricated by depositing the hybrid composite on cellulose filter paper using screen‐printing technology (NH_2_‐G/Thi/AuNPs|*μ*PADs). DPV analysis of NSE was achieved with a low detection limit of 10 pg mL^−1^ in the concentration range of 1–500 ng mL^−1^.^[^ ^329^ ^]^ This facile sensor design can encourage constructing different portable smartphone sensors, which can be extended to the detection of other biomedical markers as well.

Screen‐printed carbon electrodes modified with four different polymers combined with Au and Pd nanoparticles (polymer–Au/Pd–SA–AuNP|SPCE) were examined toward the detection of NSE (Figure 7D). Poly(o‐phenylene diamine)–Au/Pd, poly(methylene blue)–Au/Pd, poly(*N*,*N*′‐diphenyl‐p‐phenylenediamine)–Au/Pd and poly(3,3’,5,5’‐tetramethyl benzidine)–Au/Pd composites were investigated with SA–AuNP hydrogel as electron‐transfer accelerator toward the simultaneous detection of NSE in presence of three other tumor markers CA125, Cyfra21‐1, and SCCA. SWV as the method of transduction displayed excellent selectivity and stability with a low detection limit of 2.3 pg mL^−1^ in the range of 0.01–200 ng mL^−1^ NSE.^[^ ^330^ ^]^ A hybrid composite was reported for the detection of NSE using imprinted polymer, ionic liquid, and gold nanoarrays. Initially, gold nanoarrays were prepared by galvanostatic electrodeposition on porous polycarbonate modified GCE. Ionic liquid was immobilized on to the above electrodeposited gold nanoarrays. Further electropolymerization and imprinting in the presence of NSE lead to the MIP‐poly(DPIMBr)/GNA‐MVIMBF4|GCE sensor surface. Voltammetric quantification of NSE exhibited good selectively with a low detection limit of 2.6 pg mL^−1^ in the concentration range of 0.01–1.0 ng mL^−1^.^[^ ^331^ ^]^

Enzyme‐free and label‐free electrochemical immunosensor was constructed for the detection of NSE on the GCE surface using nanocomposite of AuPd nanoparticle–multiwalled carbon nanotube, ferrocenecarboxaldehyde, and chitosan hybrid hydrogel (AuPd–MWCNT/CS‐Fc|GCE). When H_2_O_2_ was present in the detection solution, enhanced signal response was achieved because of the synergetic catalysis of Fc‐CHO and AuPd–MWCNT composite toward H_2_O_2_. SWV analysis of NSE offered an LOD of 0.483 pg mL^−1^ in the concentration range of 1 pg mL^−1^–100 ng mL^−1^.^[^ ^332^ ^]^

Voltammetric detection of NSE was reported using the bioconjugates of water‐soluble pillar[6]arene functionalized PdPt porous core–shell octahedral nanodendrites, toluidine blue, and a suitable antibody (TB/WP6@PdPt‐Ab2). The proposed immunosensor system was composed of GCE/Au/Ab1/BSA/NSE. The macrocyclic host and biomimetic nanoenzymes have been effectively integrated to achieve the robust immobilization of signal molecules by host–guest molecular recognition and sensitively catalytic amplification of electrochemical signals. DPV analysis of NSE led to the LOD of 0.095 pg mL^−1^ in the concentration range of 0.3 pg mL^−1^–100 ng mL^−1^. The proposed immunosensor displayed good selectivity toward NSE in the presence of BSA, PSA, AFP, CEA, and SCCA. Practical applicability was demonstrated in the human serum samples.^[^ ^333^ ^]^

An electrochemical immunosensor for NSE was constructed using GCE modified with 6‐mercaptohexanol (MCH), thiol‐modified DNA, electrodeposited AuNPs (MCH/DNA‐SH/Au NP|GCE) as the iodide‐responsive sensing interface and the immunoprobes of bimetallic PtCu nanoparticles combined with NSE antibody (Ab2‐PtCu/NSE/Ab1‐MBs). The working principle of this immunosensor involved cascade reactions in which catalytic oxidation of iodide to iodine by PtCu nanoparticle based immunoprobes in the presence of H_2_O_2_. Subsequently, as a bridge between the tube and iodide‐responsive sensing interface, the residual iodide in tube was employed to catalyze the transition from thiol substances (RSH) to disulfide substances (RSSR) on the electrode surface. On the basis of this property, DNA‐SH and MCH reacted with H_2_O_2_ and the residual iodide to form disulfide substances and detach from the electrode surface, causing the decrease of interface resistance in different degrees. SWV analysis of NSE led to the detection limit of 52.14 fg mL^−1^ in the concentration range of 0.0001–100 ng mL^−1^. Selectivity of the sensor system was demonstrated in the presence of NSE, IgG, CEA, PSA, CA199, and CA242. The practical applicability was examined in the human serum samples.^[^ ^334^ ^]^

Ultrasensitive detection of NSE was reported by a hybrid nanocomposite of gold nanoparticles and ordered mesoporous carbon–silica which was labeled with a suitable antibody (Ab2/OMCSi‐AuNPs). An immunosensor was constructed on GCE surface with a biocompatible porous 3D graphene–starch architecture, chitosan, antibody, and BSA (BSA/NSE/Ab1/CS/3D‐GNS|GCE). Anodic stripping voltammetric (ASV) analysis of NSE led to the detection limit of 8 fg mL^−1^ in the linear range of 0.02 pg mL^−1^ to 35 ng mL^−1^.^[^ ^335^ ^]^

EIS‐based ultrasensitive detection of NSE was reported using the immunosensor composed of epoxy‐substituted‐polypyrrole P(Pyr‐Epx) polymer along with a suitable antibody and BSA which were placed on the ITO electrode surface (BSA/anti‐NSE/P(Pyr‐Epx)|ITO). The resultant bioelectrode displayed high specificity in the presence of other potential biomarkers such as CDH 22, TNF‐*α*, RACK 1, and SOX 2. The observed LOD value was 6.1 fg mL^−1^ NSE in the linear range of 0.02–7.5 pg mL^−1^.^[^ ^336^ ^]^ Similarly, another immunosensor constructed on ITO electrode modified with poly(thiophene)‐graft‐poly(methacrylamide), NSE antibody and BSA (BSA/anti‐NSE/P(Thi‐g‐MAm)|ITO). The reported impedimetric immunosensor displayed an LOD of 6.1 fg mL^−1^ NSE in the range of 0.02–4 pg mL^−1^. Selectivity was examined in the presence of IL‐1*α*, IL‐1*β*, TNF *α*, VEGF, p53, and CDH22. Practical applicability of both these impedimetric immunosensors was demonstrated in the analysis of human serum.^[^ ^208^ ^]^

Different electrochemical sensors developed for the detection of NSE using hybrid recognition matrices were summarized in Table 2. It was observed that the poly(thiophene)‐graft‐poly(methacrylamide) hybrid based immunosensor has exhibited the best LOD value of 6.1 fg mL^−1^.

#### 4.1.5 Electrochemical Sensors for Ferritin

Rapid and sensitive detection of ferritin was reported using natural cotton thread immunoassay. Colloidal gold nanorods were synthesized by a facile chemical approach. Natural cotton thread immunoassay was fabricated by depositing antibody on a cotton thread attached with glass fiber at one end (Au nanorods–GNR–Ab1|cotton thread). GNR–Ab1 bioconjugates were used as the probe. Anodic stripping voltammetric measurement of human ferritin exhibited a low detection limit of 1.58 ng mL^−1^ in the range of 5–5000 ng mL^−1^ with a response time of 30 min.^[^ ^337^ ^]^ Electrochemical detection of ferritin was investigated considering the influence of antibody orientation on the efficiency of antigen–antibody interaction. Among the examined —COOH or —NH_2_ functionalized phenyl films along with suitable antibody deposited on gold electrode (Ab/‐C_6_H_4_R/Fe@C NPs|gold), the carboxyphenyl films favored the better electron transfer by shortening the distance between redox center of the protein and the electrode surface. In addition, the external magnetic field helped to attain the best orientation which gave the enhanced signal response for ferritin. Impedimetric and Voltammetric analysis of the sensor system offered LOD values 0.40 ± 0.04 µg dL^−1^ (EIS) and 0.13 ± 0.04 µg dL^−1^ (DPV) of ferritin in the concentration range of 0.1–30 µg dL^−1^, with carboxyphenyl film. In the other case, aminoethylophenyl film offered LOD values 0.03 ± 0.002 µg dL^−1^ (EIS) and 0.02 ± 0.002 µg dL^−1^ (DPV) of ferritin in the concentration range of 0.01–20 µg dL^−1^.^[^ ^338^ ^]^

A novel immunosensor constructed with mixed metal oxide derivatives was investigated toward the selective detection of human ferritin. Cu_2_O–SiO_2_ nanostructures were achieved by magnetic stirring of the metal precursors along with CTAB using colloidal dispersion method. As synthesized composite, antiferritin and BSA were immobilized on pretreated gold electrodes (Cu_2_O–SiO_2_–Ab1–BSA|gold). DPV analysis of ferritin exhibited a detection limit of 0.4 ng mL^−1^ in two different ranges of 1.0–5.0 and 5.0–120.0 ng mL^−1^.^[^ ^339^ ^]^ An electrochemical immunosensor for the detection of ferritin was constructed using glucose oxidase, magnetic silica nanostructure, and a suitable antibody deposited on the surface of SPCE (anti‐Ft/thionine/BSA/GOD–Fe_3_0_4_–SiO_2_|SPCE). Reverse micelle method was employed for the preparation of doped nanostructures. Voltammetric analysis of ferritin with the proposed recognition matrix offered an LOD 10 pg mL^−1^ in the range of 0.1–400 ng mL^−1^.^[^ ^340^ ^]^ An immunosensor was fabricated using nanogold hollow microspheres, chitosan, antiferritin, and BSA, which were deposited on GCE surface (nanoAu–Chit–Ab1–BSA|GCE). The reported sensor successfully analyze human serum ferritin with a low detection limit of 0.1 ng mL^−1^ in the concentration range of 1–500 ng mL^−1^.^[^ ^341^ ^]^ A simple sensor interface for the detection of ferritin was reported by immobilizing antiferritin and agarose hydrogel on GCE surface (antiferritin–agarose hydrogel|GCE). Controlled experiments revealed that the reported voltammetric sensor exhibited a low detection limit of 15 ng mL^−1^ in a linear concentration range of 50–500 ng mL^−1^.^[^ ^342^ ^]^

Table 2 conveys that the biosensor systems which comprised novel metallic oxide nanoparticles and bimetallic nanoparticles have shown enhanced current sensitivity and thus superior low detection limits toward the determination of chosen vital cancer biomarkers. Selective determination of the analyte molecules has been imparted though with the use of aptamers, antibodies, etc., the current sensitivities were high enough when these metallic oxide and bimetallic nanoparticles were utilized irrespective of the signal transduction methods, CA, DPV, SWV, and EIS. Despite of the signal transduction method utilized, the LOD values of the vital cancer biomarkers have attained as low as a few femtogram per milliliter concentrations with the use of all SWV, CA, and DPV: 3.04 fg mL^−1^ PSA by SWV and DPV,^[^ ^312^ ^]^ 2 fg mL^−1^ by CA,^[^ ^304^ ^]^ 3.1 fg mL^−1^ CEA by DPV,^[^ ^320^ ^]^ 1.6 fg mL^−1^ CEA by CA,^[^ ^204^ ^]^ and 0.7 fg mL^−1^ AFP by DPV.^[^ ^206^ ^]^ From these observations, it is concluded that the signal transduction methodology could be chosen freely between these various methods, CA, DPV, and SWV, considering the practical requirements of specific applications, time of analyses, cost effectiveness, sample nature, ease‐to‐use methodology, and instrumentation.

### 4.2 Cardiac Biomarkers

#### 4.2.1 Electrochemical Quantification of Human Cardiac Troponin I

A label‐free impedimetric immunosensor was proposed for the specific detection of troponin I. The sensor surface was constructed using a hybrid nanocomposite of carboxylic acid‐functionalized third generation poly(amidoamine) dendrimer, tetra methyl benzidine, modified 6‐mercaptohexanoic acid, and antibody immobilized on gold electrode pretreated with self‐assembled monolayer (poly(amidoamine)‐TMB‐MHA‐Ab1|gold). EIS investigations lead to the selective detection of troponin I in serum with a low detection limit of 11.7 × 10^−15^ m in the concentration range of 42 × 10^−15^ m–42 × 10^−9^ m.^[^ ^343^ ^]^ A nanocomposite with unique flower‐shaped morphology was synthesized using manganese oxide and reduced graphene oxide by a facile hydrothermal method. A microfluidic immunosensor was constructed by immobilizing the above nanocomposite along with antibody on the surface of indium tin oxide (ITO) plates (MnO_2_–rGO–Ab1|ITO). Impedimetric sensor selectively detected troponin I with a low detection limit of 8.0 pg mL^−1^ in the range of 8 pg mL^−1^–20^ ^ng mL^−1^.^[^ ^344^ ^]^

**Figure 8:**
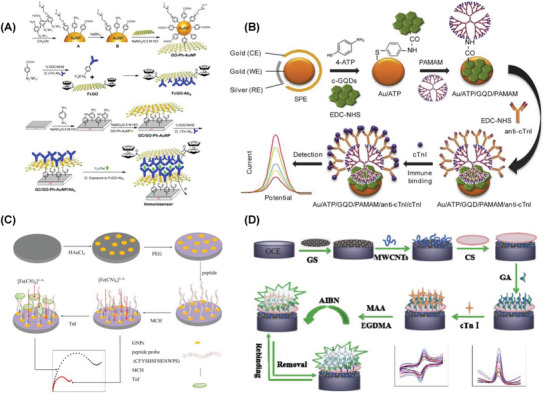
Electrochemical sensors reported for the detection of cardiac troponin I using A) GO–Ph–AuNP–Ab|GCE. Reproduced with permission.^[^ ^345^ ^]^ Copyright 2016, Elsevier. B) G‐QDs–PAMAM–Ab1|4‐ATP|SPGE. Reproduced with permission.^[^ ^347^ ^]^ Copyright 2017, Elsevier. C) AuNP–PEG–MH–CFYSHSFHENWPS|GCE. Reproduced with permission.^[^ ^348^ ^]^ Copyright 2016, Elsevier. D) Graphene–MIP–MWCNT–Chit–Glut|GCE. Reproduced with permission.^[^ ^349^ ^]^ Copyright 2017, Elsevier.

Selective voltammetric detection of cardiac troponin I was investigated using a hybrid composite of gold nanoparticles, phenyl diazonium derivatives, graphene oxide, and an antibody (**Figure** **8**A). The resultant hybrid nanocomposite was deposited on GCE surface (GO–Ph–AuNP–Ab|GCE). Ferrocene‐modified graphene oxide with antibody was used as the sensing interface. SWV quantification led to a low detection limit of 0.05 ng mL^−1^ in the concentration range of 0.05–3 ng mL^−1^ troponin I.^[^ ^345^ ^]^ Gold nanodumbbells were achieved on the gold electrode surface by facile electrodeposition (Au nanodumbbells–aptamer|gold). Resultant electrode is further modified with lyophilized aptamer. The developed voltammetric aptasensor exhibited high selectivity toward the detection of troponin I in the serum with a low detection limit of 8.0 pg mL^−1^ in the range of 0.05–500 ng mL^−1^.^[^ ^346^ ^]^

Rapid detection of troponin I was examined using a nanohybrid of graphene quantum dots, polyamidoamine deposited on screen‐printed gold electrode pretreated with 4‐aminothiophenol (G‐QDs‐PAMAM‐Ab1|4‐ATP|SPGE). Antibody was immobilized on the above‐modified electrode to construct portable immunosensor (Figure 8B). DPV analysis of troponin I led to a detection limit of 20 fg mL^−1^ in the range of 0.1–1 ng mL^−1^.^[^ ^347^ ^]^

Hydrothermal growth offered unique nanostructures of zinc oxide, which further modified with polyimide (ZnO–polyimide). A disposable EIS and Mott–Schottky sensor was developed using the above nanocomposite for the rapid and simultaneous screening of both cardiac troponins (T and I). Reported impedimetric sensor displayed a low detection limit of 1 pg mL^−1^ each troponin in human serum.^[^ ^350^ ^]^

Faradaic impedance detection of troponin I was proposed using a hybrid composite structure (Figure 8C). Electrochemically deposited gold nanoparticles and polyethylene glycol were immobilized on GCE surface (AuNP–PEG–MH–CFYSHSFHENWPS|GCE). The resultant electrode was further modified with 6‐mercapto‐1‐hexanol (MH) and special peptide CFYSHSFHENWPS served as the recognition probe. EIS analysis offered a low detection limit of 3.4 pg mL^−1^ in the concentration range of 15.5 pg mL^−1^–1.55 ng mL^−1^ troponin I.^[^ ^348^ ^]^ Voltammetric sensor was proposed for the selective detection of troponin I. A hybrid composite structure was prepared using graphene nanoplatelets, molecular imprinted polymer, multiwall carbon nanotubes, chitosan, and glutaraldehyde (Figure 8D). Imprinted polymer was synthesized on GCE surface (graphene–MIP–MWCNT–Chit–Glut|GCE). DPV analysis provided a low detection limit of 0.8 pg mL^−1^ in the linear detection range of 0.005–60 ng mL^−1^ of troponin I.^[^ ^349^ ^]^ A hybrid nanocomposite of tungsten trioxide‐reduced graphene oxide was biofunctionalized and then deposited on ITO electrode (EA/anti‐cTnI/APTES/WO_3_–RGO nanocomposite|ITO). The fabricated immunoelectrode was examined toward the detection of troponin I. A very good LOD value of 0.01 ng mL^−1^ was observed in the detection range of 0.01–250 ng mL^−1^. Good selectivity toward troponin I was achieved in the presence of CEA, cytokeratin‐19 antigen, endotheline one protein, CRP, and myoglobin.^[^ ^351^ ^]^

Impedimetric sensing of troponin I was reported using a hybrid composite of aptamer–MoS_2_ nanoconjugates and core–shell Au@SiO_2_@Au nanoparticles deposited on the surface of GCE (aptamer–MoS_2_|GCE and aptamer–Au@SiO_2_@Au|GCE). The recognition matrices displayed good sensor performance toward the selective detection of troponin I. EIS analysis of troponin I offered an LOD of 0.95 × 10^−12^ m in the range of 10 × 10^−12^ m–1 × 10^−6^ m with aptamer–MoS_2_|GCE, whereas the LOD value was 1.23 × 10^−12^ m in the range of 10 × 10^−12^ m–10 × 10^−6^ m with the aptamer–Au@SiO_2_@Au|GCE. Practical applicability of both the sensors has been demonstrated in human blood serums.^[^ ^352^ ^]^

Voltammetric immunosensor was reported for troponin I using nitrogen‐doped reduced graphene oxide which was modified with 1‐pyrenecarboxylic acid and poly(ethylene glycol) along with a suitable aptamer and then deposited on GCE surface (GCE|N‐prGO–aptamer‐(py‐PEG/py‐COOH = 20:1). Immobilization of the aptamer offered high selectivity toward the recognition of troponin I. DPV analysis of troponin I led to the LOD value of 1 pg mL^−1^ in the concentration range of 0.001–100 ng mL^−1^. The practical utility of the sensor has been demonstrated in human serum samples.^[^ ^353^ ^]^ Voltammetric quantification of troponin I was achieved using an imprinted polymer, boron nitride quantum dots and pyrrole electrodeposited on GCE surface (MIP/BNQDs|GCE). The fabricated sensor surface was successfully detected troponin I up to 0.5 pg mL^−1^ in the concentration range of 0.01–5 ng mL^−1^. Selectivity of the recognition matrix has been examined in the presence of myoglobin, BSA and cardiac troponin T. The sensor system displayed good stability, repeatability, reusability with high selectivity toward troponin I.^[^ ^354^ ^]^

A simple and sensitive electrochemical detection of troponin I was reported with a thiolated aptamer‐modified AuNPs deposited on Ti foil (Apt/AuNPs|Ti). Potential step deposition method helped to achieve pure, homogenous, and highly dense AuNPs on the Ti foil. The fabricated DPV‐based aptasensor offered an LOD value of 0.18 × 10^−12^ m in the concentration range of 1 × 10^−12^–1100 × 10^−12^ m troponin I.^[^ ^355^ ^]^ A label‐free voltammetric biosensor was reported to detect troponin I. Biorecognition matrix was prepared using a multifunctional DNA 3 way‐junction on Au nanospikes deposited on Au microgap electrode (DNA 3WJ/pAuNP|Au microgap electrode) combined with a printed circuit board chip. CV analysis of troponin I was carried out in HEPES solution and diluted human serum samples. The LOD value was 1.0 × 10^−12^ m in both the HEPES solution and in 20% diluted human serum. The reported sensor offered label‐free, simple fabrication, and easy‐to‐tailor detection elements for troponin I.^[^ ^356^ ^]^

#### 4.2.2 Selective Detection of Myoglobin

A novel “signal‐on” electrochemical immunosensor was proposed for the selective quantification of myoglobin. A sandwich‐type sensor was fabricated using copper sulfide–molybdenum disulfide hybrid nanostructures. In the second step, they were conjugated with polyclonal rabbit antihuman myoglobin antibody and then deposited on carbon fiber microelectrode (CuS–MoS_2_–PCR–Ab|CFME). The fabricated immunosensor offered a low detection limit of 1.2 pg mL^−1^ in the range of 0.005–20 ng mL^−1^ myoglobin.^[^ ^357^ ^]^

Gold nanoparticles and reduced graphene oxide based composite nanostructures were prepared by in situ electrochemical deposition on a screen‐printed electrode (AuNPs@rGO|SPCE). The characteristic reduction peak observed at ≈−0.5 V (vs Ag|AgCl) was attributed to the reduction of iron moiety present in the heme group of myoglobin. DPV‐based immunosensor displayed a low detection limit of ≈0.67 ng mL^−1^ cardiac myoglobin in the range of 1 ng mL^−1^–1400 ng mL^−1^.^[^ ^358^ ^]^ Electrochemical detection of myoglobin was reported using an aptasensor composed of gold nanoparticles and boron nitride nanosheets deposited onto the fluorine‐doped tin oxide electrode (Apt/AuNPs/BNNSs|FTO) by a spin‐coating method. A simple hydrothermal methodology was used for the synthesis of BNNSs. [Fe(CN)_6_]^3−/4−^ was used as a redox probe to monitor sensor performance. The fabricated sensor offered a detection limit of 34.6 ng mL^−1^ in the range of 0.1–100 µg mL^−1^ myoglobin. The reported sensor offered promising results for the point‐of‐care diagnosis in real samples.^[^ ^359^ ^]^ Poly(amidoamine) modified nanogold hybrid nanostructures deposited on glassy carbon electrode (AuNP‐PAMAM|GCE) was reported for the immunosensing of myoglobin with a detection limit of 3.8 pg mL^−1^ in a dynamic working range of 0.01–500 ng mL^−1^.^[^ ^360^ ^]^ Voltammetric quantification of myoglobin was reported using polypyrrole–Au nanocomposite deposited on GCE surface. The sensor surface modified with the myoglobin‐binding aptamer (PPy–Au NC‐Ab1|GCE) offered a low detection limit of 30.9 ng mL^−1^ in the wide detection range of 0.1 µg mL^−1^–0.15 mg mL^−1^.^[^ ^361^ ^]^ EIS analysis of myoglobin was reported using biosynthesized AuNPs modified with self‐assembled mono layers of 4‐ATP and an antibody deposited on ITO glass plates (antibody/4‐ATP SAM/ biosynthesized AuNP|ITO). AuNPs have been biosynthesized using a green algae *Pithophora oedogonia* as a reducing agent, using a rapid reaction (within 1 h) between Au salt and algal extract. EIS detection of myoglobin offered LOD of 5.5 ng mL^−1^ in the concentration range of 0.02–1 µg mL^−1^.^[^ ^362^ ^]^

Black phosphorus nanosheets were synthesized by liquid exfoliation approach using a surfactant. Such nanosheets were further modified with poly‐l‐lysine and an antimyoglobin aptamer and deposited on screen‐printed carbon electrodes (BP–poly‐lysine–Ab1|SPCE). Fabricated immunosensor offered the label‐free voltammetric detection of myoglobin with a record‐low detection limit of 0.13 pg mL^−1^ in a wide range of 1 pg mL^−1^ to 16 µg mL^−1^ in serum samples.^[^ ^363^ ^]^ Electrochemical detection of myoglobin was performed using an ionic liquid modified CNT. 1‐{3‐[(2‐aminoethyl)amino]propyl}‐3‐vinylimidazole bromide ionic liquid was attached on the multi‐walled carbon nanotubes and further deposited on GCE (AEAPVIB‐IL‐MWCNT|GCE). Hexacyanoferrate system was used as an electrochemical redox probe. The oxidation peak current at the potential of 0.3 V (vs SCE) was found linearly related to the myoglobin concentration. Voltammetric analysis of the fabricated sensor displayed a low detection limit of 9.7 × 10^−9^ m myoglobin in the concentration range of 60.0 × 10^−9^ m–6.0 × 10^−6^ m.^[^ ^364^ ^]^

#### 4.2.3 Electrochemical Sensors for Superoxide Radical and Superoxide Dismutase

Amperometric quantification of SOD was investigated using the nanoAu bioconjugates of cytochrome c with different alkanethiolate mono and mixed layers out of which nanoAu/MPA+MPO/Cyt c|gold offered a detection limit of 50 ng mL^−1^ SOD. Variation in the nanostructure and morphology of alkanethiolate layer at the nanoAu–Cyt c interface tremendously influenced the electrocatalytic current for superoxide which further varied sharply by the presence of superoxide dismutase. This investigation emphasized the importance of fine‐tuning the interfacial structure and morphology even at nanomaterial levels.^[^ ^365^ ^]^ Voltammetric detection of SOD1 was reported using bioconjugates of self‐assembled monolayers of gold nanoparticles, polypyrrole deposited on screen printed carbon electrode. Resultant electrode was biofunctionalized with monoclonal antibody anti‐SOD1 (anti‐SOD1‐SAM‐GNP‐PPy|SPCE) to fabricate the immunosensor. Voltammetric analysis offered a detection limit of 0.5 × 10^−9^ m SOD1 in a wide linear working range of 0.5 × 10^−9^ m –5 × 10^−6^ m.^[^ ^366^ ^]^

Amperometric detection of superoxide anion (O_2_^∙−^) was reported by bimetallic (Pt, Pd) nanocomposite in combination with MWCNT and SOD hybrid deposited on Screen‐printed gold film electrodes (Pt–Pd/MWCNTs‐SOD|SPGE). Nanohybrid was synthesized using a facile one‐step alcohol reduction process. Fabricated sensor provided high selectivity and excellent long‐term stability which offered a low detection limit of 0.71 × 10^−6^ m in a wide range of 40 × 10^−6^ –1550 × 10^−6^ m.^[^ ^367^ ^]^ Electrochemical sensor for SOD was constructed using bimetallic nanoparticles. A porous Pt–Pd nanocomposite was synthesized in two different approaches by electrodeposition on screen‐printed carbon electrodes (Pt–Pd|SPCE). SOD dispersed in nafion was deposited on those electrodes. Amperometric detection of O_2_^∙−^ offered a low detection limit of 0.13 × 10^−6^ m in the detection range of 16 × 10^−6^–1536 × 10^−6^ m.^[^ ^368^ ^]^ In another approach, bimetallic Pt and Pd nanoparticles were directly immobilized from the corresponding metal precursors on to the polydopamine coated reduced graphene oxide by magnetic stirring. The resultant hybrid nanocomposite along with SOD was deposited on screen‐printed gold electrodes (Pt–Pd–PolyDop–rGO–SOD|SPGE). Chronoamperometric detection of O_2_^∙−^ lead to a low detection limit of 2 × 10^−6^ m in the concentration range of 16–240 × 10^−6^ m.^[^ ^369^ ^]^ Metal oxide derivatives and bimetallic nanoparticles were examined toward the detection of superoxide. Enzyme‐free amperometric sensor was constructed using SOD conjugated with CuZn nanoparticles and gelatin on platinum screen printed electrodes (CuZn–SOD|PtSPE). Reported recognition matrix displayed an LOD of 0.31 × 10^−6^ m for O_2_^∙−^.^[^ ^370^ ^]^

Synergistic effect of silica, metal phosphates, and CNT toward the detection of superoxide was examined by constructing a hybrid composite structure. Initially SiO_2_ nanoparticles were prepared by reverse microemulsion. Manganese phosphate attached to silica was prepared by simple magnetic stirring of the individual metal precursors along with functionalized SiO_2_. The resultant hybrid was further treated with CNT and deposited on GCE surface. Amperometric detection of O_2_^∙−^ using SiO_2_–Mn_3_(PO_4_)_2_/MWCNT|GCE offered a low detection limit of 17.5 × 10^−9^ m in a wide linear range of 0.03 × 10^−6^–3.6 × 10^−6^ m.^[^ ^371^ ^]^ Chronoamperometric detection of O_2_^∙−^ was proposed by using a hybrid structure. A 3D DNA/Mn_3_(PO_4_)_2_ was immobilized on vertically aligned carbon nanotube array supported by polyethylene. In the next step, directly cultured living human breast carcinoma cells were attached to the above which lead to MDA‐MB‐231/Mn_3_(PO_4_)_2_/DNA/VACNT/PE hybrid film as the recognition matrix. The developed amperometric sensor offered a low detection limit of 30 × 10^−9^ m O_2_^∙−^ in a very wide concentration range of 65 × 10^−9^ m–31 × 10^−6^ m.^[^ ^372^ ^]^

#### 4.2.4 Electrochemical Quantification of Myeloperoxidase

An immunosensor was built with the aid of trimetallic CuPdPt nanowire networks for the selective detection of MPO. CuPdPt NWNWs were synthesized by a simple one‐step chemical reduction method. MPO antibody was mixed with the CuPdPt and immobilized on the GCE surface (CuPdPt‐antiMPO|GCE). A low detection limit of 33 fg mL^−1^ was achieved with the amperometric detection in a linear concentration range of 100 fg mL^−1^–50 ng mL^−1^ of MPO.^[^ ^373^ ^]^

Synergistic effect of CeO_2_ on gold nanoparticles and ionic liquid was investigated toward the detection of MPO. The resultant hybrid composite was deposited on pretreated gold electrode surface (nanoAu/CeO_2_–BMIMPF6/l–cysteine|gold). Amperometric analysis lead to a detection limit of 0.06 ng mL^−1^ in the range of 10–400 ng mL^−1^ of human serum MPO.^[^ ^374^ ^]^ A disposable electrochemical immunosensor was proposed for the selective detection of MPO. Indium tin oxide electrode was modified with an ionic liquid composite film containing gold nanoparticles, poly(o‐phenylenediamine), and carbon nanotubes (nano‐Au/PoPD–MWCNTs–IL|ITO). Voltammetric analysis offered a low detection limit of 0.07 ng mL^−1^ of MPO in the concentration range of 23.4–300 ng mL^−1^.^[^ ^375^ ^]^ A hybrid nanocomposite was reported for the detection of MPO. Layer‐by‐layer assembly approach was employed to deposit MWCNT, thionine, gold nanoparticles, and chitosan on GCE surface (MWCNT–thionine–nanoAu–Chit–antiMPO|GCE). MPO antibody was immobilized in the final step on the above‐modified electrode. Voltammetric analysis of human serum MPO exhibited a low detection limit of 1.425 ng mL^−1^ in the range of 2.5–125 ng mL^−1^.^[^ ^376^ ^]^

An amperometric immunosensor for the selective detection of human plasma MPO was proposed based on the magnetic beads. Photolithography was used for achieving the desired magnetic beads and mixed with antibody was further immobilized on screen‐printed carbon electrodes (magnetic beads–antiMPO|SPCE). The developed sensor interface displayed a detection limit of 0.4 ng mL^−1^ in a linear concentration range of 0.9–60 ng mL^−1^ MPO.^[^ ^377^ ^]^ Chronoamperometric detection of MPO in human serum was achieved using CNT wires incorporated on disposable SPCE. Antibody of MPO was further immobilized on to the modified SPCE (CNT wires–antiMPO|SPCE). Fabricated sensor system offered a detection limit of 6 and 55 ng mL^−1^ in PBS and undiluted human serum in a time duration of 30 min.^[^ ^378^ ^]^

#### 4.2.5 Electrochemical Analysis of Thrombin

**Figure 9:**
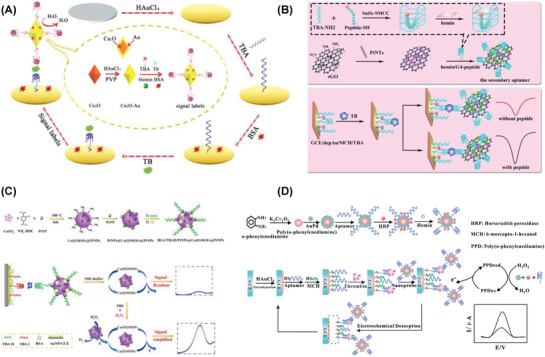
Construction of different electrochemical sensors for thrombin using A) Cu_2_O‐nanoAu–G‐quadruplex‐Ab1|GCE. Reproduced with permission.^[^ ^379^ ^]^ Copyright 2018, Elsevier. B) Hemin/G4–peptide–PtNTs@rGO|GCE. Reproduced with permission.^[^ ^199^ ^]^ Copyright 2017, Elsevier. C) PtNPs@Co(II)MOFs@PtNPs|GCE. Reproduced with permission.^[^ ^198^ ^]^ Copyright 2017, Elsevier. D) G‐quadruplex/hemin/HRP/AuPd/poly(o‐phenylenediamine)|GCE. Reproduced with permission.^[^ ^380^ ^]^ Copyright 2017, Springer‐Verlag Wien.

Selective detection of thrombin was reported using a novel octahedral Cu_2_O–Au nanocomposite with hemin/G‐quadruplex hybrid as the recognition matrix (**Figure** **9**A). In the initial step, Cu_2_O octahedral nanocrystals were synthesized from its chloride precursor by chemical precipitation method. In the second step, nanocomposite was prepared by vigorous magnetic stirring of the counterparts. In the next step, the above‐achieved nanocomposite was mixed with hemin and peptide to fabricate a signal label. Aptasensor surface was fabricated by immobilizing antibody and gold nanoparticles on pretreated GCE surface (Cu_2_O–nanoAu–G‐quadruplex–Ab1|GCE). Multiple signal amplification strategy was utilized based on the reduction of H_2_O_2_ and varying thrombin concentrations. Amperometric analysis of thrombin offered a low detection limit of 23 × 10^−15^ m in the range of 100 × 10^−15^ m–20 × 10^−9^ m.^[^ ^379^ ^]^

Highly sensitive, mediator‐free, proteinous enzyme‐free voltammetric aptasensor was proposed using hemin/G4–peptide–PtNTs@rGO bioconjugate (Figure 9B). Porous PtNTs were prepared using TeO_2_ precursor with Te@Pt core–shell structures, which further disintegrated to achieve the desired nanotubes. The nanocomposite was achieved by vigorous magnetic stirring for 16 h at normal conditions. It was further combined with hemin/G4 hybrid to construct the bionanoconjugate (hemin/G4–peptide–PtNTs@rGO|GCE). The fabricated sensor interface offered a very low detection limit of 15 × 10^−15^ m in the range of 0.05 × 10^−12^ m–60 × 10^−9^ m thrombin.^[^ ^199^ ^]^ A sandwich‐type DPV aptasensor was reported using the nanocomposite of Pt nanoparticles, functionalized cobalt‐based metal–organic frameworks (Figure 9C). Bioconjugates were fabricated using the above nanocomposite and antibody immobilized on to GCE surface (PtNPs@Co(II)MOFs@PtNPs|GCE). The developed aptasensor was highly sensitive toward thrombin which offered a low detection limit of 0.33 × 10^−15^ m in the range of 0.1 × 10^−12^ m–50 × 10^−9^ m.^[^ ^198^ ^]^

A regenerable and ultrasensitive voltammetric biosensor was proposed using G‐quadruplex/hemin/HRP/AuPd/poly(o‐phenylenediamine) bionanoconjugate (Figure 9D). Sandwich type immunosensor was prepared by immobilizing antibody and the hybrid bioconjugates on the surface of pretreated GCE (G‐quadruplex/hemin/HRP/AuPd/poly(o‐phenylenediamine|GCE). Reported recognition matrix exhibited a very good selectivity with a low detection limit of 20 × 10^−15^ m in the concentration range of 100 × 10^−15^ m–20 × 10^−9^ m thrombin.^[^ ^380^ ^]^ A novel dual‐signal ratiometric voltammetric sensor was proposed for the selective detection of thrombin using methyl blue labeled biobar‐coded AuNPs deposited on gold electrode (MB‐P3‐AuNPs|gold). A sensor probe was prepared with ferrocene labeled hairpin with “signal‐off” of MB and the “signal‐on” of ferrocene. Voltammetric analysis offered a detection limit of 1.1 × 10^−12^ m of thrombin in the range of 0.003 × 10^−9^–30 × 10^−9^ m.^[^ ^381^ ^]^ An ultrasensitive amperometric detection of thrombin was reported using the composite nanostructures of Au@GS and DNA–CoPd binary nanoparticles. CoPd nanoparticles were synthesized by heating the metal precursors at 260 °C in an inert atmosphere. Aptasensor was prepared by immobilizing the composite and an aptamer on GCE surface (Au@GS–DNA–CoPd–aptamer|GCE). The developed sensor system offered a low detection limit of 5 pg mL^−1^ in the range of 0.01–2 ng mL^−1^.^[^ ^382^ ^]^

Voltammetric analysis of thrombin was examined using Fe_2_O_3_–graphene hybrid composite with controlled and uniform structural morphology. A homemade atomic layer deposition system was used for the preparation of composite nanostructures. Immunosensor was fabricated using a thrombin aptamer mixed with the above composite, which further immobilized on GCE surface (TA/Fe_2_O_3_@graphene|GCE). The reported sensor offered a low detection limit of 1 × 10^−12^ m in the linear concentration range of 10 × 10^−12^ m to 4 × 10^−9^ m thrombin.^[^ ^383^ ^]^ Highly specific voltammetric detection of thrombin was proposed using a robust nanocomposite of TiO_2_, CNT, and 3‐[(2‐hydroxypropyl)imino]indoline‐2‐one based Schiff base deposited on GCE (TiO_2_/MWCNT/chitosan/SB|GCE). The sensor displayed high selectivity toward thrombin with a very low detection limit of 1 × 10^−15^ m in the detection range of 0.05 × 10^−12^ m–10 × 10^−9^ m.^[^ ^384^ ^]^

Bimetallic nanoparticles were investigated toward the voltammetric quantification of thrombin. Pd–Au nanoparticles were synthesized by a facile magnetic stirring of the metal precursors. An aptamer and horseradish peroxidase were mixed with the above nanoparticles and further deposited on a gold electrode surface (Pd–Au–HRP|gold). Resultant immunosensor offered a low detection limit of 3 × 10^−12^ m in the concentration range of 0.05 × 10^−9^–50 × 10^−9^ m.^[^ ^385^ ^]^

A magnetic force assisted amperometric sensor was reported for the selective detection of thrombin using functionalized magnetic nanoparticles with a suitable antibody, Toluidine blue O and a conducting polymer layer (poly‐(2,2′:5´,5″‐terthiophene‐3′‐p‐benzoic acid) deposited on the surface of SPCE (pTBA/Apt/thrombin/MNP@Ab‐TBO|SPCE). Magnetic field was used for controlling the reaction and the removal of unbound bioconjugates from the sensor surface without washing. Amperometric detection of thrombin using the proposed sensor offered an LOD of 0.49 × 10^−9^ m in the dynamic range of 1.0 × 10^−9^ to 500 × 10^−9^ m.^[^ ^386^ ^]^

Voltammetric detection of thrombin was reported based on rolling circle amplification using AuNPs, polyadenine, aptamer immobilized on the gold electrode surface (Apt/polyadenine/AuNP|gold). Electrocatalytic reduction of H_2_O_2_ was considered for evaluating the sensor performance. The observed LOD value was 35 × 10^−15^ m in the concentration range of 0.1 × 10^−12^ m–10 × 10^−9^ m thrombin. The sensor displayed very good selectivity toward thrombin. The sensor system facilitated the detection of thrombin without any fussy modification process.^[^ ^387^ ^]^ Impedimetric signal amplification strategies were proposed for the ultrasensitive quantification of thrombin using Ag and Au nanoparticles. A sandwich type immunosensor was constructed by immobilizing the aptamers along with the metal nanoparticles. Enhancement treatments by silver and gold resulted the lowest detection limits 0.3 × 10^−12^ and 0.45 × 10^−12^ m thrombin, respectively, in the linear detection range of 0.1 × 10^−12^–100 × 10^−12^ m.^[^ ^388^ ^]^ A label‐free impedimetric detection of *α*‐thrombin was reported by an aptamer‐functionalized gold‐coated nanoporous anodized alumina or aluminum oxide (thiolated aptamer/Au|NAAO) membranes with a four‐electrode setup. The achieved LOD was 10 × 10^−12^ m of *α*‐thrombin in the presence of 500 × 10^−6^ m HSA. The reported sensor offered very good selectivity and sensitivity in the presence of high concentrations of interfering molecules. The authors claimed that the proposed scheme used minimum reagents/sample preparation steps, and it could be readily integrated to the miniaturized sensor systems.^[^ ^389^ ^]^ A novel impedimetric sensor for thrombin was reported using a nanohybrid of fluorinated graphene oxide and iron‐based metal−organic gel along with a suitable aptamer deposited on disposable electrically printed electrodes (Apt/FGO@Fe–MOG|DEP chip). Highly selective detection of thrombin was reported with an LOD of 2.2 ng mL^−1^ in the concentration range of 2–14 ng mL^−1^. Practical applicability of the proposed aptasensor was demonstrated successfully in human serum samples.^[^ ^390^ ^]^ An electrochemical sensor was constructed for thrombin by using novel silver nanoclusters. Ag NCs were achieved by in situ chemical reduction using NaBH_4_. Multiple amplification strategy was employed for the label‐free voltammetric detection of thrombin. The reported sensor system exhibited a low detection limit of 0.1 × 10^−15^ m in the linear concentration range of 1 × 10^−15^ m–10 × 10^−9^ m.^[^ ^391^ ^]^

**Table** **3** summarizes the electrochemical sensors for the selected cardiac biomarkers—troponin I, myoglobin, superoxide anion, myeloperoxidase, and thrombin. It was observed that the hybrid recognition matrix containing black phosphorus nanosheets offered LOD value of 0.524 pg mL^−1^ for myoglobin (CV) and trimetallic CuPdPt nanostructures offered LOD value of 33 fg mL^−1^ for myeloperoxidase (CA). Polymer‐based hybrid nanocomposites offered the LOD of 0.5 × 10^−9^ m superoxide anion (CV) and 11.7 × 10^−15^ m troponin I (EIS). Ag nanoclusters based hybrid has exhibited the best LOD of 0.1 × 10^−15^ m for thrombin (DPV). It can be concluded that the hybrid metallic nanocomposites offered promising ultrasensitive LOD values for the selected cardiac biomarkers.

**Table 3:** Summary of the electrochemical biosensors reported for the detection of cardiac biomarkers (troponin I, myoglobin, superoxide anion, myeloperoxidase, thrombin)

Biomarker	Recognition matrix|electrode	Method	Conc range	LOD	Interferents	Real samples	Ref.
Troponin I (cTnI)	NanoAu–phenyl diazonium derivative–GO|GCE	SWV	0.05–3 ng mL^−1^	0.05 ng mL^−1^	Glu, AA, UA, BSA, IgG, cTnT, avidin, MYO, creatine, creatinine	Serum	^[^ ^345^ ^]^
	EA/anti‐cTnI/APTES/WO_3_‐RGO nanocomposite|ITO	DPV	0.01–250 ng mL^−1^	0.01 ng mL^−1^	CEA, CYFRA, ET, CRP, MYO	Serum	^[^ ^351^ ^]^
	nMn_3_O_4_‐rGO|ITO	EIS	8 pg mL^−1^–20^ ^ng mL^−1^	8.0 pg mL^−1^	MYO, cTnC, cTnT, BNP	Serum	^[^ ^344^ ^]^
	Gold nanodumbbells‐lyophilized aptamer|gold	DPV	0.05–500 ng mL^−1^	8.0 pg mL^−1^	Bilirubin, EDTA, HGB, HSA, heparin	Serum	^[^ ^346^ ^]^
	6‐mercapto‐1‐hexanol‐CFYSHSFHENWPS‐NanoAu–PEG|GCE	EIS	15.5 pg mL^−1^–1.55 ng mL^−1^	3.4 pg mL^−1^	AFP, IgG, BSA, egg protein	Serum	^[^ ^348^ ^]^
	ZnO‐polyimide|Sensor Array	EIS	0.1 pg mL^−1^–100 ng mL^−1^	1 pg mL^−1^	*α*‐cTnT, *α*‐cTnI, BSA	Serum	^[^ ^350^ ^]^
	N‐prGO–aptamer–(py‐PEG/py‐COOH = 20:1)|GCE	DPV	0.001–100 ng mL^−1^	1 pg mL^−1^	BSA, BNP, lyzoyme	Serum	^[^ ^353^ ^]^
	Graphene–MIP‐MWCNT–Chit–Glut|GCE	DPV	0.005–60 ng mL^−1^	0.8 pg mL^−1^	CEA, BSA, NSE, HIV‐p24, HCG	Serum	^[^ ^349^ ^]^
	MIP/BNQDs|GCE	DPV	0.01–5 ng mL^−1^	0.5 pg mL^−1^	MYO, BSA, cTnT	Plasma	^[^ ^354^ ^]^
	Aptamer–MoS_2_|GCE aptamer–Au@SiO_2_@Au|GCE	EIS	10 × 10^−12^ m–10 × 10^−6^ m 10 × 10^−12^ m–1 × 10^−6^ m	1.23 × 10^−12^ m 0.95 × 10^−12^ m	CK‐MB, MYO	Serum	^[^ ^352^ ^]^
	DNA 3WJ/pAuNP/Au microgap electrode	CV	0 × 10^−12^ m –100 × 10^−9^ m	1.0 × 10^−12^ m	cTnI, hemocyanin, MYO, HGB, albumin	Serum	^[^ ^356^ ^]^
	Apt/AuNPs|Ti	DPV	1 × 10^−12^–1100 × 10^−12^ m	0.18 × 10^−12^ m	Chol, BSA, MYO, GOx, Ins, IgG	Serum	^[^ ^355^ ^]^
	4‐aminothiophenol‐GQDs‐polyamidoamine|SPGE	DPV	0.1–1 ng mL^−1^	20 fg mL^−1^	HsC, CCP, IL‐1*β*, Gliadin	Serum	^[^ ^347^ ^]^
	MHA/TMB/Den/anti‐TnI|Gold	EIS	42 × 10^−15^ m–42 × 10^−9^ m	11.7 × 10^−15^ m	cTnT, sk‐TnI, CEA, IL‐6, PSA, TB, CRP	Serum	^[^ ^343^ ^]^
Myoglobin	Apt/AuNPs/BNNSs|FTO	DPV	0.1–100 µg mL^−1^	34.6 ng mL^−1^	HGB, GOx, Ins, SOX	Serum	^[^ ^359^ ^]^
	MBA/(PPy–Au)/APTES|GCE	DPV	0.1 µg mL^−1^–0.15 mg mL^−1^	30.9 ng mL^−1^	GOx, Cyt c, HGB, hemin	Muscles	^[^ ^361^ ^]^
	Antibody/4‐ATP SAM/biosynthesized AuNP/APTES|ITO	EIS	0.02–1 µg mL^−1^	5.5 ng mL^−1^	Zn^2+^, Cu^2+^, SO_4_^2−^, Glu, sucrose, AA, UA, amylum	Serum	^[^ ^362^ ^]^
	AuNPs@rGO|SPCE	DPV	1 ng mL^−1^–1400 ng mL^−1^	0.67 ng mL^−1^	BSA, avidin, cTnI	Serum	^[^ ^358^ ^]^
	APVIMBr ionic liquid–MWCNT|GCE	DPV	60.0 × 10^−9^ m–6.0 × 10^−6^ m	9.7 × 10^−9^ m	HGB, BSA, Cyt C, Ova, AA, Cys, His	Serum	^[^ ^364^ ^]^
	AuNP–PAMAM|GCE	CSV	0.01–500 ng mL^−1^	3.8 pg mL^−1^	CA 19‐9, CA 125, CEA, AFP, IgG	Serum	^[^ ^360^ ^]^
	CuS–MoS_2_/carbon fiber microelectrode	CV	0.005–20 ng mL^−1^	1.2 pg mL^−1^	CA 19‐9, CA 125, CEA, AFP	Serum	^[^ ^357^ ^]^
	Poly‐l‐lysine‐antimyoglobin–black phosphorus nanosheets|SPCE	CV	1 pg mL^−1^–16 µg mL^−1^	0.13 pg mL^−1^	HGB, BSA	Serum	^[^ ^363^ ^]^
Superoxide anion O_2_^∙−^	PtPd–polydopamine–rGO|SPGE	CA	16 × 10^−6^ m–240 × 10^−6^ m	2 × 10^−6^ m	AA, AP, DA, UA, Glu	DMEM medium	^[^ ^369^ ^]^
	Pt–Pd/MWCNTs‐SOD|SPGE	Amp	40 × 10^−6^ –1550 × 10^−6^ m	0.71 × 10^−6^ m	AA, UA, AP, DA, Glu, Fru,	DMEM medium	^[^ ^367^ ^]^
	Gelatin CuZn–SOD|Pt SPE	Amp	–	0.31 × 10^−6^ m	Acetylsalicylic acid, aspirin, aspirin C	Brain tissue	^[^ ^370^ ^]^
	Pt–Pd nanocomposite|SPCE	Amp	16 × 10^−6^–1536 × 10^−6^ m	0.13 × 10^−6^ m	AA, DA, UA, AP, GSH, Glu	DMEM medium	^[^ ^368^ ^]^
	NanoAu/MPA+MPO/Cyt c|gold	Amp	–	50 ng mL^−1^	–	–	^[^ ^365^ ^]^
	MDA–MB‐231/Mn_3_(PO_4_)_2_/ DNA/VACNT|PE	CA	65 × 10^−9^ m–31 × 10^−6^ m	30 × 10^−9^ m	AA, K^+^, Na^+^, Cl^−^, CO_3_^2−^, NO_3_^−^, UA, NO, H_2_O_2,_ ClO^−^	AMEM medium	^[^ ^372^ ^]^
	SiO_2_–Mn_3_(PO_4_)_2_/MWCNTs|GCE	Amp	0.03 × 10^−6^–3.6 × 10^−6^ m	17.5 × 10^−9^ m	UA, AA, DA, Cys, H_2_O_2_	HeLa cells	^[^ ^371^ ^]^
	Anti‐SOD1‐SAM‐GNP‐PPy|SPCE	CV	0.5 × 10^−9^ m–5 × 10^−6^ m	0.5 × 10^−6^ m	MYO, Cyt c	Keratino‐cyte cells	^[^ ^366^ ^]^
Myeloperoxidase	CNT wires|SPCE	CA	0–4 µg mL^−1^	55 ng mL^−1^	–	Serum	^[^ ^378^ ^]^
	MWCNT–thionine–AuNP–chitosan|GCE	CV	2.5–125 ng mL^−1^	1.42 ng mL^−1^	–	Serum	^[^ ^376^ ^]^
	Magnetic beads|SPCE	Amp	0.9–60 ng mL^−1^	0.4 ng mL^−1^	BSA	Serum, plasma	^[^ ^377^ ^]^
	NanoAu/PoPD–MWCNTs‐IL|ITO	Amp	23.4–300 ng mL^−1^	0.07 ng mL^−1^	cTnI, CRP, CK‐MB, MYO	Serum	^[^ ^375^ ^]^
	Nanogold/CeO_2_–BMIMPF6/l‐gysteine|gold	Amp	10–400 ng mL^−1^	0.06 ng mL^−1^	cTnI, CK‐MB, Cys, MYO, CRP, HCG	Serum	^[^ ^374^ ^]^
	Trimetallic CuPdPt|GCE	Amp	100 fg mL^−1^–50 ng mL^−1^	33 fg mL^−1^	BSA, Cys, Glu, DA	Serum	^[^ ^373^ ^]^
Thrombin	Apt/FGO@Fe−MOG|DEP chip	EIS	2–14 ng mL^−1^	2.2 ng mL^−1^	BSA, IgG, streptavidin	Serum	^[^ ^390^ ^]^
	pTBA/Apt/thrombin/MNP@Ab‐TBO|SPCE	Amp	1.0 × 10^−9^ m–500 × 10^−9^ m	0.49 × 10^−6^ m	–	Serum	^[^ ^386^ ^]^
	Au@GS and DNA‐CoPd|GCE	Amp	0.01–2 ng mL^−1^	5 pg mL^−1^	BSA, HGB, muramidase	Serum	^[^ ^382^ ^]^
	Thiolated aptamer/Au|NAAO	EIS	0× 10^−9^–500 × 10^−9^ m	10 × 10^−12^ m	HSA	Serum	^[^ ^389^ ^]^
	Nano Pd–Au|Gold	DPV	0.05 × 10^−9^–50 × 10^−9^ m	3 × 10^−12^ m	BSA, IgG, HSA	Serum	^[^ ^385^ ^]^
	MB‐P3‐AuNPs|gold	ACV	0.003 × 10^−9^–30 × 10^−9^ m	1.1 × 10^−12^ m	BSA, MYO, HGB	Serum	^[^ ^381^ ^]^
	TA/Fe_2_O_3_@graphene|GCE	DPV	10 × 10^−12^ m–4 × 10^−9^ m	1 × 10^−12^ m	BSA, lysozyme, HSA, insulin	–	^[^ ^383^ ^]^
	NanoAg or nanoAu–aptamers|AvGECs	EIS	0.1 × 10^−12^–100 × 10^−12^ m	0.3 × 10^−12^ m 0.45 × 10^−12^ m	Fbr, Alb, Cyt c, IgG, ProThr	Serum	^[^ ^388^ ^]^
	Apt/polyadenine/AuNP|gold	DPV	0.1 × 10^−12^ m–10 × 10^−9^ m	35 × 10^−15^ m	BSA, IgG, HGB	Serum	^[^ ^387^ ^]^
	Cu_2_O–Au nanocomposite|GCE	CA	100 × 10^−15^ m–20 × 10^−9^ m	23 × 10^−15^ m	BSA, HGB, CEA	Serum	^[^ ^379^ ^]^
	G‐quadruplex/hemin/HRP/AuPd/poly(o‐phenylenediamine)|GCE	DPV	100 × 10^−15^ m–20 × 10^−9^ m	20 × 10^−15^ m	BSA, AFP, Arg	Serum	^[^ ^380^ ^]^
	Hemin/G4–peptide–PtNTs@rGO bioconjugate|GCE	DPV	0.05 × 10^−12^ m–60 × 10^−9^ m	15 × 10^−15^ m	CEA, AFP, HGB	Serum	^[^ ^199^ ^]^
	TiO_2_/MWCNT/chitosan/SB|GCE	DPV	0.05 × 10^−12^ m–10 × 10^−9^ m	1 × 10^−15^ m	BSA, OVA, BHGB, lysozyme, IgG	Serum	^[^ ^384^ ^]^
	PtNPs@Co(II)MOFs @PtNPs|GCE	DPV	0.1 × 10^−12^ m–50 × 10^−9^ m	0.33 × 10^−15^ m	Hb, PCT, cystatin C, influenza	Serum	^[^ ^198^ ^]^
	Ag nanoclusters	DPV	1 × 10^−15^ m–10 × 10^−9^ m	0.1 × 10^−15^ m	BSA, CEA, AFP	Serum	^[^ ^391^ ^]^

CSV – cathodic stripping voltammetry; ACV – alternating current voltammetry; cTnC – cardiac troponin C; cTnT – cardiac troponin T; CK‐MB – creatin kinase; CYFRA – cytokeratin‐19 antigen; ET – endotheline one protein; CRP – C‐reactive protein; MYO – myoglobin; BNP – B‐type natriuretic peptide; EDTA – ethylenediaminetetraacetic acid; HIV‐p24 – human immunodeficiency virus p24; HCG – human chorionic gonadotropin; Chol – cholesterol; GOx – glucose oxidase; SOX – sarcosine oxidase; Ins – insulin; HsC – hepatitis C antigen; CCP – cyclic citrullinated peptide antigen; IL‐1*β* – interleukin 1‐beta; Cyt c – cytochrome c; OVA – ovalbumin; AP – 4‐acetaminophen; Fru – fructose; NO – nitric oxide, H_2_O_2_ – hydrogen peroxide; ClO^−^ – hypochlorite; Fbr – fibrinogen; ProThr – prothrombin; PCT – procalcitonin, AvGECs – avidin graphite epoxy conductive paste electrodes; DMEM medium – Dulbecco's modified Eagle's medium (DMEM) cell culture medium containing 10% fetal bovine serum and 1% penicillin/streptomycin; AMEM medium – advance minimum essential medium (MEM) containing 10% fetal bovine serum and 1% penicillin.

### 4.3 Inflammatory Disease Biomarkers

#### 4.3.1 Selective Detection of Nitric Oxide

**Figure 10:**
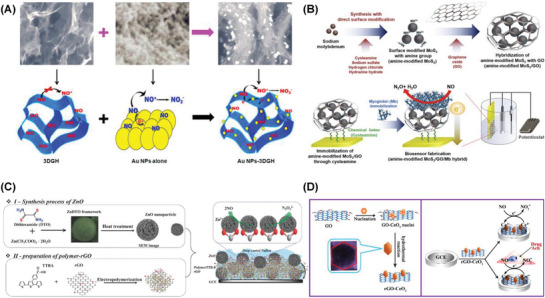
Different approaches toward the electrochemical detection of nitric oxide using A) AuNPs−3DGH|GCE. Reproduced with permission.^[^ ^393^ ^]^ Copyright 2015, American Chemical Society. B) Amine‐MoS_2_–GO–Myo|Cys–gold. Reproduced with permission.^[^ ^394^ ^]^ Copyright 2017, Elsevier. C) ZnO–polyPBA‐TT‐rGO|GCE. Reproduced with permission.^[^ ^395^ ^]^ Copyright 2017, Wiley‐VCH Verlag GmbH & Co. KGaA, Weinheim. D) rGO–CeO_2_|GCE. Reproduced with permission.^[^ ^396^ ^]^ Copyright 2015, Elsevier.

Graphene–gold nanocomposite was synthesized sonochemically in an inert atmosphere. Obtained nanocomposite was deposited on freshly polished GCE surface (graphene–nanoAu|GCE) and investigated its application toward the detection of nitric oxide. Sensor performance was demonstrated using linear sweep voltammetry and amperometry as transduction methods. The fabricated enzyme‐free sensor displayed a very high selectivity toward nitric oxide with a low detection limit of 0.048 × 10^−6^ m and in the range of 10 × 10^−6^–5000 × 10^−6^ m using LSV whereas Amperometry led to a low detection limit of 0.25 × 10^−6^ m in the range of 1 × 10^−6^–10 × 10^−6^ m.^[^ ^392^ ^]^ Highly selective detection of nitric oxide was reported using the hybrid composite constructed by gold nanoparticles deposited on 3D graphene hydrogel (**Figure** **10**A). A facile one‐step hydrothermal approach was employed for the preparation of hydrogel. Nanocomposite was obtained by a simple in situ chemical reduction. Resultant nanocomposite was immobilized on GCE surface using nafion as binder (AuNPs–3DGH|GCE). The fabricated enzyme‐free amperometric sensor displayed a very good low detection limit of 9 × 10^−9^ m nitric oxide in the range of 0.2 × 10^−6^–6 × 10^−6^ m.^[^ ^393^ ^]^

Selective amperometric quantification of nitric oxide was reported by using hybrid composite of amine‐modified molybdenum disulfide nanoparticles, graphene oxide, and myoglobin (Figure 10B). Resultant hybrid was deposited on gold electrodes pretreated with cysteamine (amine–MoS_2_–GO–Myo|Cys–gold). Fabricated sensor displayed a very good low detection limit of 3.6 × 10^−9^ m nitric oxide in the range of 3.6 × 10^−9^–36 × 10^−9^ m.^[^ ^394^ ^]^

Synergistic effect of zinc oxide, conductive polymer, and carbon nanomaterials was examined toward the selective detection of nitric oxide (Figure 10C). Porous ZnO nanoparticles were derived from zinc‐dithiooxamide by calcination at 700 °C. A hybrid nanocomposite was prepared by mixing ZnO, poly(3´‐(p‐benzoic acid)‐2,2´:5´,2´´‐terthiophene), and reduced graphene oxide. Resultant hybrid was deposited on GCE surface (ZnO–polyPBA‐TT–rGO|GCE). Amperometric sensor probe displayed a low detection limit of 7.7 × 10^−9^ m in a wide dynamic range of 0.019 × 10^−6^–76 × 10^−6^ m nitric oxide. The practical applicability was demonstrated by quantifying the amount of nitric oxide in the living cells.^[^ ^395^ ^]^

In another report, the combination of cerium oxide and reduced graphene oxide was examined for the detection of nitric oxide (Figure 10D). Nanocomposite with controlled hexagonal nanocrystals was achieved by hydrothermal approach. The obtained nanocomposite was deposited on GCE (rGO–CeO_2_|GCE). Amperometric sensor displayed a low detection limit of 9.6 × 10^−9^ m in a wide dynamic range of 18.0 × 10^−9^ m–5.6 × 10^−6^ m and also exhibited a good practical applicability in the detection of nitric oxide released from living cells.^[^ ^396^ ^]^

Gold and silver bimetallic nanoparticles were examined for the detection of nitric oxide. Au/Ag nanoclusters were synthesized from the relevant metal precursors and poly(acrylamide‐*co*‐diallyl dimethylammonium chloride) by vigorous magnetic stirring at normal temperatures. Resultant Au/Ag nanoclusters embedded in matrix were deposited on GCE surface (Au–Ag NC|GCE). Amperometric quantification of nitric oxide displayed high selectivity and response time of 1 s with a low detection limit of 10 × 10^−9^ m in the range of 10 × 10^−9^ m–0.9 × 10^−6^ m.^[^ ^397^ ^]^ A hybrid constructed with gold nanoparticles, chitosan, and cellulose acetate was reported for the voltammetric detection of nitric oxide. Obtained composite was immobilized on transparent carbon ultramicroelectrode arrays (CA/CS/GNP|1.54T‐CUMEA). SWV analysis of nitric oxide displayed a low detection limit of 0.2 × 10^−6^ m in the range of 1 × 10^−6^–100 × 10^−6^ m.^[^ ^398^ ^]^ Electrocatalytic effect of two different composite nanostructures was investigated toward the detection of nitric oxide. Fe_2_O_3_ and Prussian blue modified graphene oxide were deposited on Pt electrodes (GO–Fe_2_O_3_|Pt and GO–PB|Pt). Among the both composites, GO–Fe_2_O_3_|Pt gave the best performance toward electrocatalytic oxidation of nitric oxide with a low detection limit of 13.04 × 10^−6^ m in the range of 90.9 × 10^−6^–444.4 × 10^−6^ m.^[^ ^399^ ^]^

A novel third‐generation nitric oxide biosensor was reported with an enzyme functionalized hybrid nanocomposite of MWCNTs, 1‐*n*‐butyl‐3‐methylimidazolium tetrafluoroborate (BMIMBF_4_) deposited on a pyrolytic graphite electrode surface (NOR/MWCNTs/BMIMBF_4_|PGE). SWV analysis of nitric oxide offered an LOD value of 0.07 × 10^−6^ m in the concentration range of 0.23 × 10^−6^–4.76 × 10^−6^ m. The reported nanocomposite NOR/MWCNTs/BMIMBF_4_ has displayed excellent bioactivity.^[^ ^400^ ^]^ Selective amperometric and in situ monitoring of nitric oxide was achieved using a hybrid nanocomposite of iron phthalocyanine and nitrogen‐doped graphene, nafion, and poly‐l‐lysine deposited on ITO electrode surface (N‐G/FePc/nafion/PLL|ITO). A very good electrocatalytic activity toward the detection of nitric oxide was observed due to the synergetic effect of N‐G and FePc. LOD of 0.18 × 10^−6^ m was reported in the concentration range of 0.18 × 10^−6^–400 × 10^−6^ m. The proposed sensor system offered promising results in the in vivo monitoring of nitric oxide in the complex biological system.^[^ ^401^ ^]^ SWV analysis of nitric oxide was reported using a hybrid nanocomposite of carboxylated single‐walled carbon nanotubes, lipid bilayer [1,2‐di‐(9Z‐octadecenoyl)‐sn‐glycero‐3‐phosphoethanolamine (DOPE), 1,2‐di‐(9Z‐octadecenoyl)‐3‐trimethylammonium‐propane (DOTAP), 1,2‐distearoyl‐sn‐glycero‐3‐phosphoethanolamine‐polyethylene glycol (DSPE‐PEG)] and nitric oxide reductase enzyme deposited on pyrolytic graphite electrode (NOR/(DOPE:DOTAP:DSPE‐PEG)SWCNT|PGE). The reported sensor matrix displayed high selectivity toward nitric oxide with an LOD of 0.13 × 10^−6^ m in the range of 0.44 × 10^−6^–9.09 × 10^−6^ m. The proposed sensor exhibited excellent biomimetic features.^[^ ^402^ ^]^

Amperometric analysis of nitric oxide was reported using AuNPs, mercaptopropionic acid, chitosan, Cyt c, and nafion deposited on gold electrode (nafion/Cyt c/CS‐3‐MPA‐AuNPs/cysteamine‐MPA|gold). Good electrocatalytic activity was observed toward the reduction of nitric oxide. Reported amperometric sensor displayed an LOD value of 45 × 10^−9^ m in the range of 10 × 10^−6^–215 × 10^−6^ m. The developed sensor system was quite selective and stable.^[^ ^403^ ^]^ An interesting voltammetric sensor was proposed for the detection of nitric oxide using honeybee silk heme, MWCNT‐modified glassy carbon electrodes (heme‐silk/MWCNT|GCE) as the sensing platform. Proposed sensor exhibited a low detection limit of 2 × 10^−9^ m in the range of 19 × 10^−9^ m–1.9 × 10^−6^ m nitric oxide.^[^ ^404^ ^]^

#### 4.3.2 Electrochemical Quantification of Tumor Necrosis Factor *α*

**Figure 11:**
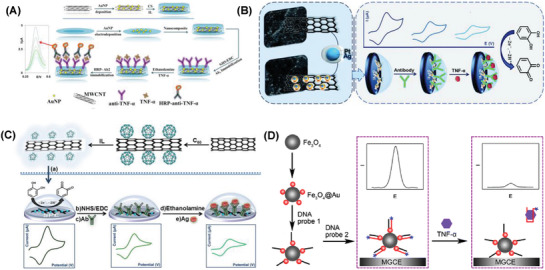
Fabrication approaches of electrochemical sensors for TNF‐*α* using A) nanoAu–CNT–IL/Ab|GCE. Reproduced with permission.^[^ ^406^ ^]^ Copyright 2015, Wiley‐VCH Verlag GmbH & Co. KGaA, Weinheim. B) Ag/Pt–CNTs|SPCE. Reproduced with permission.^[^ ^407^ ^]^ Copyright 2015, Royal Society of Chemistry. C) C60–CNT–IL/Ab|SPE. Reproduced with permission.^[^ ^408^ ^]^ Copyright 2015, Elsevier. D) Fe_3_O_4_–nanoAu–DNA|GCE. Reproduced with permission.^[^ ^409^ ^]^ Copyright 2017, Springer‐Verlag GmbH Austria.

Hybrid nanocomposite of gold nanoparticles and graphene derivatives was reported for the voltammetric detection of TNF‐*α*. Nanocomposite achieved with gold nanoparticles, reduced graphene oxide, antibody, 4‐carboxyphenyl, 4‐aminophenyl phosphoryl choline was deposited on gold electrode (nanoAu–graphene derivatives/gold). The label‐free voltammetric sensor based on aryl diazonium salt coupling chemistry displayed a low detection limit of 0.1 pg mL^−1^ TNF‐*α* in the concentration range of 0.1–150 pg mL^−1^.^[^ ^405^ ^]^ Another voltammetric sensor was reported with a robust nanocomposite produced by a single synthesis step using gold nanoparticles instead of fullerene, MWCNT and 1‐buthyl‐3‐methylimidazolium bis(trifluoro‐methyl sulfonyl)imide ionic liquid (**Figure** **11**A). A sandwich immunoassay was constructed along with the hybrid composite and antibody immobilized on GCE surface (nanoAu–CNT–IL/Ab|GCE). Resultant immunosensor displayed a low detection limit of 2.0 pg mL^−1^ TNF‐*α* in the range of 6.0–100 pg mL^−1^.^[^ ^406^ ^]^

Hybrid nanocomposite constructed with bimetallic nanoparticles was developed for the voltammetric measurement of TNF‐*α* (Figure 11B). Bimetallic Ag/Pt core–shell nanoparticles were achieved using step‐by‐step mixing of the metal precursors to the mixture of MWCNTs and chitosan. Resultant composite was coated on graphite screen‐printed electrodes (Ag/Pt–CNTs|SPCE). The enzyme‐free sensor displayed a very good electrocatalytic activity with a low detection limit of 1.6 pg mL^−1^ in the range of 6.0–60 pg mL^−1^ TNF‐*α*.^[^ ^407^ ^]^ A hybrid nanocomposite of fullerene, MWCNT, and 1‐butyl‐3‐methylimidazolium‐bis(trifluoromethyl sulfonyl)imide ionic liquid was obtained by ultrasonic agitation (Figure 11C). A suitable antibody was mixed with the above composite and further immobilized on graphite screen printed electrode (C60–CNT–IL/Ab|SPE). The reported electrochemical immunosensor offered a low detection limit of 2.0 pg mL^−1^ in the range of 5.0–75 pg mL^−1^ TNF‐*α*.^[^ ^408^ ^]^

An impedance sensor was proposed for the specific detection of TNF‐*α* using a hybrid nanostructure formed by gold nanoparticles and reduced graphene oxide. In the next step, the composite and an antibody were mixed which further deposited on an indium tin oxide microelectrode array (nanoAu–rGO–antibody|ITO). The impedimetric sensor exhibited a detection limit of 0.43 pg mL^−1^ in the range of 1–1000 pg mL^−1^ using [Fe(CN)_6_]^3−/4−^ couple as the redox probe.^[^ ^410^ ^]^ The combination of Fe_3_O_4_ magnetic nanoparticles, gold nanoparticles, and DNA were examined toward the selective detection of TNF‐*α* (Figure 11D). Resultant composite was deposited on GCE (Fe_3_O_4_–nanoAu–DNA|GCE). A signal label was prepared with DNA and methylene blue. The reported SWV sensor displayed a low detection limit of 10 pg mL^−1^ in the range of 10 pg mL^−1^–100 ng mL^−1^.^[^ ^409^ ^]^ The electrochemical aptasensor has exhibited a good selectivity toward TNF‐*α* in the coexistence of other interleukins.

An amperometric sensor for the simultaneous detection of TNF‐*α* and interleukin‐*β* was reported by using nanobioconjugates. 4‐carboxyphenyl‐functionalized double‐walled carbon nanotubes and suitable antibody were deposited on SPCE (4‐carboxyphenyl‐DWCNT|SPCE) and poly‐HRP‐streptavidin. The fabricated sandwich type immunosensor exhibited a very good selectivity with a low detection limit of 0.85 pg mL^−1^ TNF‐*α* in the range of 1–200 pg mL^−1^ and 0.38 pg mL^−1^ IL‐1*β* in the range of 0.5–100 pg mL^−1 [^ ^411^ ^]^

Impedimetric detection of TNF‐*α* in human saliva was reported using a fully integrated electrochemical biosensor platform with eight gold microelectrodes. The approach offered enhanced sensitivity at very less time of analysis. The recognition matrix was composed of electrodeposited 4‐carboxymethylaniline, antibody functionalized with carboxyl diazonium deposited on gold microelectrodes (CMA/Ab|gold microelectrode). EIS analysis of the sensor system offered an LOD of 3.1 pg mL^−1^ in the range of 1–100 pg mL^−1^ TNF‐*α*. The sensor performance was examined in PBS buffer, artificial saliva, and real human saliva.^[^ ^412^ ^]^ EIS analysis of TNF *α* was reported using poly(3‐thiophene acetic acid) combined with an antibody deposited on hydroxy‐functionalized ITO surface (BSA/Ab/P3|ITO). LOD value was 3.7 fg mL^−1^ in the range of 0.01 pg mL^−1^–2 pg mL^−1^. Practical utility of the sensor was demonstrated in human serum and saliva samples.^[^ ^413^ ^]^ Impedimetric sensor for TNF‐*α* was reported using reduced graphene oxide, AuNPs and a suitable antibody deposited on ITO microdisk electrodes (Ab/rGO/AuNP|ITO). [Fe(CN)_6_]^3−/4−^ was used as a redox probe for the quantification of TNF‐*α* in human serum based on the resistance changes at 2 Hz (Δ*R* at 2 Hz). The proposed EIS‐based sensor displayed a good LOD of 0.67 pg mL^−1^ in the range of 1–1000 pg mL^−1^.^[^ ^414^ ^]^

#### 4.3.3 Electrochemical Sensors for CRP

**Figure 12:**
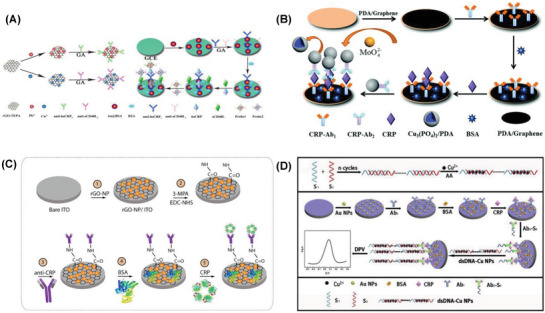
Strategies employed in the fabrication of CRP immunosensors using A) Ab1S/GA/Au@BSA|GCE. Reproduced with permission.^[^ ^415^ ^]^ Copyright 2015, Elsevier. B) Cu_3_(PO_4_)_2_/PDA/Ab2|GCE. Reproduced with permission.^[^ ^416^ ^]^ Copyright 2017, Royal Society of Chemistry. C) rGO–AuNP|ITO. Reproduced with permission.^[^ ^417^ ^]^ Copyright 2016, Elsevier. D) Ab1/AuNPs|GCE. Reproduced with permission.^[^ ^418^ ^]^ Copyright 2017, Elsevier.

Hybrid nanostructures of gold nanoparticles and glutaraldehyde were investigated toward the detection of CRP (**Figure** **12**A). Gold nanoparticles–bovine serum albumin nanospheres combined with antibody were deposited on GCE surface (Ab1S/GA/Au@BSA|GCE). A sensing probe was constructed by Pb^2+^/Cu^2+^ ions on reduced graphene oxide–tetraethylenepentamine with glutaraldehyde and antibody (rGO–TEPA–GA–Ab2). The developed sensor system displayed a very good low detection limit of 16.7 pg mL^−1^ CRP in the range of 0.05–100 ng mL^−1^ and also demonstrated a good practical validity in the clinical serum samples.^[^ ^415^ ^]^

A hybrid nanocomposite was constructed with copper phosphate nanospheres, polymerized dopamine hydrochloride, and antibody Ab2, which was further deposited on GCE surface (Cu_3_(PO_4_)_2_/PDA/Ab2|GCE) (Figure 12B). Square wave voltammetric detection of CRP was achieved using molybdophosphate as the redox signal probe with a low detection limit of 0.13 pg mL^−1^ in the linear range of 0.5 pg mL^−1^–1 ng mL^−1^.^[^ ^416^ ^]^

Impedimetric detection of CRP was reported using a hybrid nanocomposite of GO and AuNPs electrodeposited on ITO plates (rGO–AuNP|ITO) (Figure 12C). [Fe(CN)_6_]^3−/4−^ was employed as the redox couple lead to a detection limit of 0.08 ng mL^−1^ in the range of 1–1000 ng mL^−1^ CRP in serum.^[^ ^417^ ^]^ Voltammetric immunosensor was reported for CRP using copper nanoparticles–HCR as a signal probe and gold nanobioconjugates (Figure 12D) as the recognition matrix. A sandwich type bioconjugates were prepared using electrodeposited gold nanoparticles and antibody Ab1 immobilized on GCE surface (Ab1/AuNPs|GCE). The immunosensor offered a low detection limit of 0.33 fg mL^−1^ in the range of 1.0 fg mL^−1^ to 100 ng mL^−1^ with good practical demonstration in the human serum samples.^[^ ^418^ ^]^

Nonenzymatic voltammetric sensor for CRP was reported using the hybrid nanocomposite based on molybdenum disulfide–polyaniline–gold nanoparticles deposited on GCE surface (MoS_2_–PANI–GNPs|GCE). The developed DPV sensor exhibited a detection limit of 40 pg mL^−1^ in the range of 0.2–80 ng mL^−1^.^[^ ^419^ ^]^

Amperometric detection of CRP was reported with the aid of sandwich nanobioconjugates of bimetallic nanoparticles. Hollow Ag/Pt nanoparticles were prepared by mixing the metal precursors at 160 °C. They were combined with an antibody and further deposited on GCE surface pretreated with amine‐functionalized graphene oxide (Ag/Pt–Ab|NH_2_–GO–GCE). Sandwich electrochemical immunosensor displayed a low detection limit of 0.17 ng mL^−1^ CRP in the range of 0.5–140 ng mL^−1^.^[^ ^420^ ^]^

Square wave voltammetric detection of CRP was reported using a hybrid bioconjugate at a nanocomposite modified electrode. The bioconjugate constructed using 3‐aminopropyl‐triethoxysilane, gold nanoparticles, antibody loaded with Zn^2+^ ions on silica microspheres (Zn^2+^/Ab/AuNPs@Si MSs) was used as the immunoprobe. The sensing electrode was constructed by using RNA aptamer, gold nanoparticles, 6‐Mercapto‐1‐hexanol, and CRP antibody which were deposited on GCE surface (CRP/MCH/RNA/AuNPs|GCE). The proposed sensor system displayed a very good selectivity toward CRP and the observed detection limit was 0.0017 ng mL^−1^ in the range of 0.005–125 ng mL^−1^ CRP.^[^ ^421^ ^]^ Voltammetric analysis of CRP was carried out on screen‐printed graphene electrode modified with l‐cysteine, AuNPs and a suitable antibody (Ab/l‐Cys/Au|SPGE). DPV analysis of CRP led to an LOD of 1.5 ng mL^−1^ in the range of 0.01–150 µg mL^−1^. Practical application was demonstrated in the human serum samples.^[^ ^422^ ^]^ Voltammetric detection of CRP was achieved using a hybrid composite of ZnO/porous carbon matrix and ionic liquid which was incorporated in a carbon paste electrode (BSA/anti‐CRP/ZnO/MPC/IL|CPE). The calculated LOD value was 5.0 pg mL^−1^ in the range of 0.01–1000 ng mL^−1^. Selectivity of the reported sensor was assessed in the presence of BSA, IgG, glucose, ascorbic acid, and uric acid.^[^ ^423^ ^]^

A metal‐free, antibody‐free sensor was constructed using an electropolymerized poly(3,4‐ethylenedioxythiophene)‐zwitterionic phosphoryl choline on GCE surface (PEDOT‐PC|GCE). DPV as the method of transduction offered a low detection limit of 37 × 10^−9^ m CRP in the concentration range of 10 × 10^−9^–160 × 10^−9^ m. Fabricated electrode displayed high selectivity with a very good stable sensor response.^[^ ^424^ ^]^ A disposable EIS sensor was reported for CRP using disposable ITO sheets coated with 11‐cyanoundecyltrimethoxysilane, PAMAM dendrimers and an antibody (BSA/anti‐CRP PANAM/11‐CUTMS/OH|ITO). EIS analysis led to the LOD value of 0.34 fg mL^−1^ in the range of 21–6148 fg mL^−1^ CRP. Recognition behavior was monitored by a single frequency impedance technique.^[^ ^425^ ^]^

#### 4.3.4 Electrochemical Analysis of Interleukin‐6 (IL‐6)

**Figure 13:**
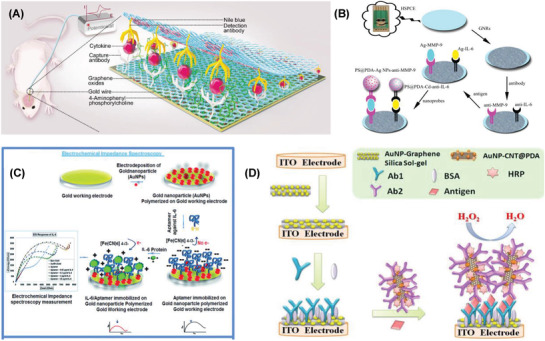
Immunosensors reported for the electrochemical analysis of IL‐6 using A) Ph‐NH_2_ Au‐ph‐GO‐PPC/Ab1|gold. Reproduced with permission.^[^ ^426^ ^]^ Copyright 2017, American Chemical Society. B) Ab_2_/Ag/Ab_1_|GNR‐SPCE. Reproduced with permission.^[^ ^427^ ^]^ Copyright 2014, Elsevier. C) NanoAu–IL‐6 aptamer|Alkanethiol–gold. Reproduced with permission.^[^ ^428^ ^]^ Copyright 2016, Royal Society of Chemistry. D) IL‐6/Ab_1_/AuNP–graphene–silica|ITO. Reproduced with permission.^[^ ^429^ ^]^ Copyright 2014, Elsevier.

A hybrid sandwich immunosensor was reported for the in vivo detection of IL‐6. Gold electrode surface was modified with 4‐aminophenyl derivatives, which further attached with 4‐aminophenyl phosphoryl choline, graphene oxide, and IL‐6 monoclonal antibody (Ph‐NH_2_ Au‐ph‐GO‐PPC/Ab1|gold). A redox probe constructed with graphene oxide and Nile blue nanocomposite has offered a low detection limit of 1 pg mL^−1^ in the range of 1–300 pg mL^−1^ IL‐6. In addition, a gold wire‐based nanosandwich device was successfully demonstrated for the in vivo monitoring of IL‐6 secretion in cells and live mice (**Figure** **13**A).^[^ ^426^ ^]^

Square wave voltammetric sensor for IL‐6 was reported using a nanocomposite of polystyrene, polydopamine, silver or cadmium nanoparticles (PS@PDA–Ag/Cd NPs) as the label (Figure 13B). Screen‐printed carbon electrode modified with graphene nanoribbon, antibody–silver nanoparticles–antibody sandwich (Ab_2_/Ag/Ab_1_|GNR–SPCE) was used as the sensor probe. The reported immunosensor displayed a detection limit of 0.1 pg mL^−1^ IL‐6 in the concentration range of 1 pg mL^−1^–1 µg mL^−1^. Sensor performance was successfully demonstrated in the clinical samples of patients.^[^ ^427^ ^]^

An interesting impedimetric sensor was reported for the detection of IL‐6 in sweat (Figure 13C). A hybrid was fabricated using electrodeposited gold nanoparticles and IL‐6 aptamer which were attached on the gold electrode pretreated with HS‐(CH2)_11_(OCH_2_CH_2_)_3_OH alkane thiol (nanoAu–IL‐6 aptamer|alkanethiol–gold). Potassium ferricyanide was used as the redox couple for observing the variation in EIS signal. The proposed label free nanoaptasensor offered a low detection limit of 0.02 pg mL^−1^ in the range of 0.02–20 pg mL^−1^ IL‐6. Practical applicability has been demonstrated with different concentrations of IL‐6 spiked in the artificial sweat.^[^ ^428^ ^]^

EIS‐based aptasensor was constructed for the detection of IL‐6 using a nanocomposite of polypyrrole, gold nanoparticles, and aptamer immobilized on SPGE (Apt/AuNPs/PPy|SPGE). The aptasensor exhibited a good impedimetric response with an LOD of 0.33 pg mL^−1^ in the range of 1 pg mL^−1^–15 µg mL^−1^. The sensor performance was tested in human serum samples.^[^ ^430^ ^]^ Selective amperometric quantification of IL‐6 was reported using a hybrid composite constructed with gold nanoparticles‐graphene‐silica sol–gel, antibody, and bovine serum albumin deposited on ITO electrode (Figure 13D) (IL‐6/Ab_1_/AuNP–graphene–silica|ITO). In addition to that, a highly selective bioconjugate label was prepared using gold nanoparticles‐polydopamine on carbon nanotubes bound with antibody (HRP–Ab_2_–AuNP–PDA@CNT). Reported IL‐6 electrochemical sensor displayed a low detection limit of 0.3 pg mL^−1^ in the working range of 1–40 pg mL^−1^ with a fast response time of 3 s.^[^ ^429^ ^]^

Differential pulse voltammetric analysis of IL‐6 was demonstrated using a hybrid bioconjugate of silver nanoparticle–hollow titanium phosphate functionalized with antibody Ab_2_–AgNP–TiP as a signal probe. Electrode was constructed with iron oxide and antibody (Fe_3_O_4_–Ab1 conjugates) deposited on 96‐well magnetized microplate. DPV response was measured based on the amount of Ag ions released after treating with nitric acid. The proposed sensor exhibited a low detection limit of 0.1 pg mL^−1^ in the range of 0.5 pg mL^−1^–10 ng mL^−1^ IL‐6.^[^ ^431^ ^]^

Bimetallic nanoparticles based immunosensor was developed for the sub‐picomolar voltammetric quantification of IL‐6. Au–Pd bimetallic nanoparticles were achieved by electrodeposition using cyclic voltammetry at 25 mV s^−1^ in 20 cycles. Graphene oxide was electrochemically reduced and combined with the AuPd and an antibody which further deposited on electrically heated carbon electrode. A bionanolabel was constructed with silver nanoparticles functionalized polystyrene and polydopamine which further coated with IL‐6 antigen (IL‐6‐PS@PDA–AgNPs). LSV as transduction method offered a detection limit of 0.059 pg mL^−1^ IL‐6 in the wide linear range of 0.1 pg mL^−1^–0.1 µg mL^−1^.^[^ ^432^ ^]^ In another approach, bimetallic Pd–Pt nanoparticles were examined toward the voltammetric detection of IL‐6. Pd–Pt nanoparticles were synthesized from their metal precursors by vigorous magnetic stirring. Hybrid nanocomposite was constructed with the Pd–Pt, an antibody, and bovine serum albumin that further deposited on GCE surface (BSA/anti‐IL‐6/Pt–Pd NPs|GCE). LSV analysis lead to a low detection limit of 0.032 pg mL^−1^ in the range of 0.1 pg mL^−1^–2 ng mL^−1^ with a very good selectivity toward IL‐6.^[^ ^433^ ^]^ Amperometric magneto immunosensor was developed for the selective detection of IL‐6 using a hybrid conjugate constructed with carboxyl‐functionalized magnetic microparticles, antibody, and poly‐HRP–streptavidin conjugates deposited on screen‐printed carbon electrode (poly‐HRP–strept–biotin–anti‐IL‐6–IL‐6–anti‐IL‐6–MB). The proposed magnetic immunosensor exhibited a low detection limit of 0.39 pg mL^−1^ IL‐6 in the concentration range of 1.75 to 500 pg mL^−1^. This sensor was demonstrated for the successful detection of IL‐6 in saliva and urine and also exhibited very strong stable and reproducible signal response.^[^ ^434^ ^]^

Electrochemical sensors reported for the inflammatory disease biomarkers were summarized in **Table** **4**. It was observed that the recognition matrix containing amine functionalized MoS_2_–GO hybrid displayed the LOD value of 3.6 × 10^−9^ m nitric oxide. Whereas, the metallic nanocomposite comprising either gold nanoparticles alone or in combination with graphene derivatives displayed the LOD values of 0.1 pg mL^−1^ for TNF‐*α*, 0.02 pg mL^−1^ for IL‐6, and 0.33 fg mL^−1^ for CRP. It can be concluded from Table 4 that the best electrochemical sensor for inflammatory disease biomarkers can be fabricated with the help of hybrid metallic nanocomposite derivatives.

**Table 4:** Summary of the electrochemical biosensors reported for the detection of inflammatory disease biomarkers (nitric oxide, tumor necrosis factor‐*α*, C‐reactive protein, Interleukin‐6)

Biomarker	Recognition matrix|electrode	Method	Concentration range	LOD	Interferents	Real samples	Ref.
Nitric oxide	GO–Fe_2_O_3_ |Pt	LSV	90.9 × 10^−6^–444.4 × 10^−6^ m	13.04 × 10^−6^ m	Cl^−^, NO_3_^−^, NO_2_^−^, SO_3_^2−^, SO_4_^2−^	–	^[^ ^399^ ^]^
	Graphene–Au nanocomposite|GCE	LSV an Amp	10 × 10^−6^ –5000 × 10^−6^ m 1 × 10^−6^ –10 × 10^−6^ m	0.048 × 10^−6^ m 0.25 × 10^−6^ m	UA, AA, DA, Glu, H_2_O_2_	–	^[^ ^392^ ^]^
	CA/CS/GNP|1.54T‐CUMEA	SWV	1 × 10^−6^ –100 × 10^−6^ m	0.2 × 10^−6^ m	UA, AA, DA, Glu, FBS, NO_2_^−^, H_2_O_2_	Endothelial cells	^[^ ^398^ ^]^
	N‐G/FePc/nafion/PLL |ITO	Amp	0.18 × 10^−6^–400 × 10^−6^ m	0.18 × 10^−6^ m	Cl^−^, NO_3_^−^, NO_2_^−^, SO_4_^2−^, Glu	Endothelial cells	^[^ ^401^ ^]^
	NOR/(DOPE:DOTAP: DSPE‐PEG) SWCNT| PGE	SWV	0.44 × 10^−6^–9.09 × 10^−6^ m	0.13 × 10^−6^ m	NO_3_^−^, NO_2_^−^, Arg, AA, Glu	–	^[^ ^402^ ^]^
	NOR/MWCNTs/ BMIMBF_4_|PGE	SWV	0.23–4.76 µM	0.07 × 10^−6^ m	NO_3_^−^, NO_2_^−^, Glu, AA	–	^[^ ^400^ ^]^
	Nafion/Cyt c/CS‐3‐MPA‐AuNPs/cysteamine‐MPA|gold	Amp	10 × 10^−6^–215 × 10^−6^ m	45 × 10^−9^ m	NO_2_^−^, AA	–	^[^ ^403^ ^]^
	PADA‐Au_25_/Ag_75_ NCs|GCE	Amp	10 × 10^−9^ m–0.9 × 10^−6^ m	10 × 10^−9^ m	Glu, CO_2_, urea, oxalate, Cl^−^, NO_3_^−^, NH_3_	–	^[^ ^397^ ^]^
	rGO–CeO_2_|GCE	Amp	18.0 × 10^−9^ m–5.6 × 10^−6^ m	9.6 × 10^−9^ m	K^+^, Na^+^, Ca^2+^, Cl^−^, CO_3_^2−^, NO_3_^−^, AA, UA, DA	A549 cells	^[^ ^396^ ^]^
	AuNPs–3DGH|GCE	Amp	0.2 × 10^−6^–6 × 10^−6^ m	9 × 10^−6^ m	K^+^, Na^+^, Cl^−^, NO_2_^−^, NO_3_^−^, SO_4_^2−^, AA, DA	B16‐F10, JB6‐C30 cells	^[^ ^393^ ^]^
	Poly(TTBA‐rGO)/ZnO|GCE	Amp	0.019 × 10^−6^–76 × 10^−6^ m	7.7 × 10^−6^ m	AA, AP, UA, Arg, O_2_^−,^ H_2_O_2_	DMEM medium	^[^ ^395^ ^]^
	Amine‐modified MoS_2_–GO–myoglobin |cysteamine–gold	Amp	3.6 × 10^−9^–36 × 10^−9^ m	3.6 × 10^−6^ m	NO_2_^−^, HCO_3_^−^, AA	–	^[^ ^394^ ^]^
	Heme‐silk/ MWCNT|GCE	LSV	19 × 10^−9^ m–1.9 × 10^−6^ m	2 × 10^−6^ m	NO_2_^−^, NO_3_^−^, DA, AP, UA, FBS, dissolved O_2_	–	^[^ ^404^ ^]^
Tumor necrosis factor‐*α* (TNF‐*α*)	Fe_3_O_4_–nanoAu–DNA|GCE	SWV	10 pg mL^−1^–100 ng mL^−1^	10 pg mL^−1^	IL‐1, IL‐2, IL‐6, IL‐12, IFN‐*γ*	Serum	^[^ ^409^ ^]^
	CMA/Ab|gold microelectrode	EIS	1–100 pg mL^−1^	3.1 pg mL^−1^	IL‐1, IL‐8	Artificial, real saliva	^[^ ^412^ ^]^
	C60–CNT–IL/Ab|SPCE	DPV	5.0–75 pg mL^−1^	2.0 pg mL^−1^	BSA	Serum	^[^ ^408^ ^]^
	NanoAu–CNT–IL/Ab|GCE	DPV	6.0–100 pg mL^−1^	2.0 pg mL^−1^	BSA, HGB	Serum	^[^ ^406^ ^]^
	Ag@Pt‐CNTs core–shell nanocomposite|SPCE	DPV	6.0–60 pg mL^−1^	1.6 pg mL^−1^	BSA	Serum	^[^ ^407^ ^]^
	4‐carboxyphenyl‐DWCNT|SPCE	Amp	1–200 pg mL^−1^	0.85 pg mL^−1^	IL‐1*α*, IL‐1*β*, TNF‐*β*, TNF‐RI, TNF‐TII, APN, BSA, Chol, CRP, HGB, IL‐6, IL‐8, TGF‐*β*1, BIL, Cp, GHRL, LEP	Saliva, serum	^[^ ^411^ ^]^
	Ab/rGO–AuNP|ITO	EIS	1–1000 pg mL^−1^	0.67 pg mL^−1^	Serum, BSA, CRP	Serum	^[^ ^414^ ^]^
	NanoAu–rGO–antibody|ITO	EIS	1–1000 pg mL^−1^	0.43 pg mL^−1^	–	–	^[^ ^410^ ^]^
	NanoAu–graphene derivatives|gold	SWV	0.1–150 pg mL^−1^	0.1 pg mL^−1^	BSA, PSA, CA125, IgG	Live BV2 cells	^[^ ^405^ ^]^
	BSA/Ab/P3|ITO	EIS	0.01 pg mL^−1^–2 pg mL^−1^	3.7 fg mL^−1^	SOX2, MAGE1, RACK1, HER3, VEGFR, Alb, biotin	Saliva, serum	^[^ ^413^ ^]^
C‐reactive protein (CRP)	Ab/l‐Cys/Au/SPGE	DPV	0.01–200 µg mL^−1^	1.5 ng mL^−1^	–	Serum	^[^ ^422^ ^]^
	Hollow Ag/Pt–Ab/NH_2_–fGO|GCE	Amp	0.5–140 ng mL^−1^	0.17 ng mL^−1^	BSA, Gly, HCG, Glutamic Acid	Serum	^[^ ^420^ ^]^
	CRP/ MCH/RNA/AuNPs|GCE	SWV	0.005–125 ng mL^−1^	1.7 pg mL^−1^	AFP, CEA, PSA, Glu	Serum	^[^ ^421^ ^]^
	poly(3,4‐EDT)‐phosphorylcholine|GCE	DPV	10 × 10^−9^–160 × 10^−9^ m	37 × 10^−9^ m	BSA	Serum	^[^ ^424^ ^]^
	MoS_2_–PANI–GNPs|GCE	DPV	0.2–80 ng mL^−1^	40 pg mL^−1^	BSA, Gly, HCG, Glu	Serum	^[^ ^419^ ^]^
	BSA/anti‐CRP/ZnO/ MPC/IL|CPE	DPV	0.01–1000 ng mL^−1^	5.0 pg mL^−1^	AA, UA, BSA, IgG, Glu	Serum	^[^ ^423^ ^]^
	NanoAu–BSA–Ab|GCE	DPV	0.05–100 ng mL^−1^	16.7 pg mL^−1^	AA, UA, BSA, Glu, SAA, MCP‐1, Ntn1	Serum	^[^ ^415^ ^]^
	Cu_3_(PO_4_)_2_/PDA/Ab|GCE	SWV	0.5 pg mL^−1^–1 ng mL^−1^	0.13 pg mL^−1^	CEA, CA125, cTnI	Serum	^[^ ^416^ ^]^
	rGO‐AuNP|ITO	EIS	1–1000 ng mL^−1^	0.08 ng mL^−1^	–	Serum	^[^ ^417^ ^]^
	BSA/anti‐CRP PANAM/ 11‐CUTMS/OH|ITO	EIS	21–6148 fg mL^−1^	0.34 fg mL^−1^	HER‐3, HSP70, Haptoglobin	Serum	^[^ ^425^ ^]^
	Electrodeposited nanoAu–Ab1|GCE	DPV	1.0 fg mL^−1^–100 ng mL^−1^	0.33 fg mL^−1^	AFP, CEA, l‐Cys, Lys, UA	Serum	^[^ ^418^ ^]^
Interleukin‐6 (IL‐6)	Au‐ph‐NH_2_ Au‐ph‐GO‐PPC/Ab1|gold	SWV	1–300 pg mL^−1^	1 pg mL^−1^	BSA, CA125, PSA, IgG	Mouse brain	^[^ ^426^ ^]^
	poly‐HRP–strept‐biotin–anti‐IL‐6–IL‐6–anti‐IL‐6–MB|SPCE	Amp	1.75–500 pg mL^−1^	0.39 pg mL^−1^	IL‐8, IgG, PRL, cortisol	Saliva, urine	^[^ ^434^ ^]^
	Apt/AuNPs/PPy|SPGE	EIS	1 pg mL^−1^–15 µg mL^−1^	0.33 pg mL^−1^	BSA, CEA, MUC1, MUC4, MUC16	Serum	^[^ ^430^ ^]^
	IL‐6/Ab_1_/AuNP–graphene–silica|ITO	Amp	1–40 pg mL^−1^	0.3 pg mL^−1^	AFP, HCG, PSA	Serum	^[^ ^429^ ^]^
	PS@PDA–Ag/Cd NPs|GCE	SWV	1 pg mL^−1^–1 µg mL^−1^	0.1 pg mL^−1^	BSA, CEA, cTnI, IgG	Serum	^[^ ^427^ ^]^
	Fe_3_O_4_–Ab_1_|magnetized microplate	DPV	0.5 pg mL^−1^ –10 ng mL^−1^	0.1 pg mL^−1^	TNF‐*α*, CEA, IgG	Serum	^[^ ^431^ ^]^
	Au–Pd–rGO–Ab1|carbon electrode	LSV	0.1 pg mL^−1^–0.1 µg mL^−1^	0.059 pg mL^−1^	BSA, CEA, cTnI, MMP2	Serum	^[^ ^432^ ^]^
	BSA/anti‐IL‐6/Pt–Pd NPs|GCE	LSV	0.1 pg mL^−1^–2 ng mL^−1^	0.032 pg mL^−1^	BSA, CEA, cTnI, MMP2	Serum	^[^ ^433^ ^]^
	Electrodeposited nanoAu–aptamer/AT|gold	EIS	0.02–20 pg mL^−1^	0.02 pg mL^−1^	BSA	Artificial sweat	^[^ ^428^ ^]^

A549 – Human lung carcinoma cells; APN – Adiponectin; Chol – cholesterol; CRP – C‐reactive protein; IL‐6 – interleukin 6; IL‐8 – interleukin‐8; TGF‐b1 – transforming growth factor b1; BIL – bilirubin; Cp – ceruloplasmin; GHRL – ghrelin; LEP – leptin; MCP‐1 – Monocyte chemoattractant protein‐1; SOX2 – sex determining region Y‐box 2; MAGE1 – Melanoma‐associated antigen 1; RACK1 – receptor for activated C kinase 1; HER3 – human epidermal growth factor receptor; VEGFR – vascular endothelial growth factor receptor; MUC1 – Mucin 1; MUC4 – Mucin 4; MUC16 – Mucin 16; MMP‐2 – matrix metalloproteinase‐2; HSP70 – heat shock protein; PRL – prolactin.

### 4.4 Diabetes Biomarkers

#### 4.4.1 Glucose Electrochemical Sensors

Amperometric quantification of glucose was proposed using a highly ordered rhizobia‐like nickel nanoparticles/titanium oxide nanowires composite array. Hydrothermal synthesis at 210 °C and the annealing in inert atmosphere yielded highly ordered nanowires. The fabricated Amperometric sensor system (rhizobia‐like Ni–TiO_2_|GCE) offered a low detection limit of 0.18 × 10^−6^ m in a wide linear range of 1 × 10^−6^ m–7 × 10^−3^ m glucose.^[^ ^435^ ^]^

Mixed metallic oxide derivatives were tested toward the detection of glucose. A nonenzymatic electrochemical glucose sensor was developed using TiO_2_/Co_3_O_4_|FTO composite. The resultant sensor displayed a low detection limit of 0.3396 × 10^−6^ m in a wide linear range up to 3.0 × 10^−3^ m with a fast response time less than 5 s.^[^ ^436^ ^]^ A binder‐free electrode was constructed by cobalt oxide nanosheets, polypyrrole nanowires core–shell 3D micro/nanoheterostructures on nickel foam (Co_3_O_4_/PPy|NF). Chronoamperometric detection of glucose lead to a low detection limit of 0.74 × 10^−6^ m in the concentration range of 2.0 × 10^−6^ m to 5.0 × 10^−3^ m.^[^ ^437^ ^]^ In another binderless approach, Zn‐doped Co_3_O_4_ was deposited on fluorine‐doped tin oxide plates (Zn–Co_3_O_4_|FTO). Amperometric analysis displayed a very good selectivity and the sensor response was rapid. The sensor worked efficiently in the linear range of 5 × 10^−6^ –0.62 × 10^−3^ m and offered a low detection limit of 2 × 10^−6^ m.^[^ ^438^ ^]^

In the similar procedure, Ni‐doped molybdenum disulfide nanoparticles/reduced graphene oxide (Ni‐MoS_2_/rGO) composite was prepared for the detection of glucose. The developed nonenzymatic amperometric glucose sensor (Ni‐MoS_2_/rGO|GCE) was highly selective toward glucose. Low detection limit of 2.7 × 10^−6^ m was observed in the range of 0.005 × 10^−3^ –8.2 × 10^−6^ m.^[^ ^439^ ^]^ Rapid response time, wide linear range, good reproducibility and ideal stability were claimed as the advantages of the proposed sensor.

Impedimetric sensor for the selective detection of glucose was proposed using 3‐aminophenylboronic acid (APBA) and reduced graphene oxide‐based nanocomposite. One‐step electrochemical deposition was used to deposit the composite on screen‐printed electrodes (APBA‐rGO|SPCE). Reported sensor displayed a low detection limit of 30 × 10^−6^ m in the range of 0.1 × 10^−3^–50 × 10^−3^ m.^[^ ^440^ ^]^

#### 4.4.2 Selective Quantification of Glycated Hemoglobin (HbA1c)

A composite film of gold nanoparticles embedded on N‐doped graphene nanosheets was deposited on fluorine‐doped tin oxide glass electrode (nanoAu–N–graphene|FTO). A very stable and selective sensor performance was observed with a low detection limit of 0.2 × 10^−6^ m HbA1c in the linear range of 0.3 × 10^−3^ –2 × 10^−3^ m.^[^ ^441^ ^]^ Selective voltammetric detection of HbA1c was reported using electrochemically deposited bimetallic Au–Pt hybrid nanocomposite. Bioconjugates of fructosyl amine oxidase and polyindole‐5‐carboxylic acid mixed with Au–Pt were immobilized on gold electrode surface (FAO/AuNPs–PtNPs/PIN5COOH|gold). The fabricated voltammetric sensor displayed a low detection limit of 0.2 × 10^−6^ m in the range of 0.1 × 10^−6^ m–1 × 10^−3^ m HbA1c.^[^ ^442^ ^]^ Square wave voltammetric detection of HbA1c was reported using the bioconjugates of G20 aptamer modified gold nanoparticles. Screen‐printed carbon electrodes array modified with the hybrid and an aptamer was used as the working electrode (nanoAu–G20|SPCE). The voltammetric aptasensor exhibited a low detection limit of 0.2 ng mL^−1^ of HbA1c in the concentration range of 100 pg mL^−1^–100 ng mL^−1^.^[^ ^443^ ^]^

Amperometric quantification of HbA1c was reported using the electropolymerized composite formed by poly(2,2′:5′,5″‐terthiophene‐3′‐p‐benzoic acid), multiwalled carbon nanotubes, toluidine blue O, and an aptamer on a screen printed carbon electrode (aptamer/TBO/pTBA@MWCNT|SPCE). The proposed disposable sensor exhibited a low detection of 3.7 × 10^−9^ m in the range of 0.006 × 10^−6^–0.74 × 10^−6^ m HbA1c.^[^ ^444^ ^]^ A nonenzymatic voltammetric sensor for HbA1c was reported using PtNPs/rGO–MWCNT nanocomposite deposited on gold surface (PtNPs/rGO–MWCNT|gold). The sensor system exhibited a low detection limit of 0.1 × 10^−6^ m in the concentration range of 0.05 × 10^−6^ m–1 × 10^−3^ m with a quick sensor response time of less than 3 s.^[^ ^445^ ^]^

Voltammetric quantification of HbA1c was achieved using gold nanoparticles, 12‐phosphotungstic acid and tubular TiO_2_ and fructosyl amino acid oxidase‐based hybrid composite. The mixed composite was deposited on ITO glass plate (FAO/GNPs–PTA–TiO_2_|ITO). The reported sensor displayed a low detection limit of 0.5 × 10^−6^ m in the range of 0.5 × 10^−6^ m–2 × 10^−3^ m with a fast response time of 3 s.^[^ ^446^ ^]^

Electrochemical sensors reported for the detection of selected diabetic and renal biomarkers were summarized in **Table** **5**. It was observed that the recognition matrix containing Ni@PANI/MIP hybrid displayed the LOD value of 0.2 × 10^−9^ m creatinine. Whereas, the metallic nanocomposite comprising rhizobia‐like Ni–TiO_2_ displayed the LOD value of 3.7 × 10^−9^ m HbA1c and aptamer/TBO/pTBA@MWCNT hybrid offered the LOD of 0.18 × 10^−6^ m glucose. Table 5 conveys that the best electrochemical sensor for diabetic and renal biomarkers can be fabricated with the help of hybrid metallic nanocomposite derivatives.

**Table 5:** Summary of the electrochemical biosensors reported for the detection of diabetic (glucose, HbA1c) and renal biomarkers (creatinine)

Biomarker	Recognition matrix | electrode	Method	Concentration range	LOD	Interferents	Real samples	Ref.
Glucose	APBA–rGO|SPCE	EIS	0.1 × 10^−3^–50 × 10^−3^ m	30 × 10^−6^ m	NaCl, KCl, CaCl_2_, GA, Cys, BSA, AA, HSA, DA, urea, LA	Serum	^[^ ^440^ ^]^
	Ni–MoS_2_/rGO|GCE	Amp	0.005× 10^−3^–8.2 × 10^−3^ m	2.7 × 10^−6^ m	NaCl, DA, AA, UA, V_B_, acetamidophenol	Serum	^[^ ^439^ ^]^
	Zn–Co_3_O_4_|FTO	Amp	5 × 10^−6^ m–0.62 × 10^−3^ m	2 × 10^−6^ m	AA, AC, UA, Fru	–	^[^ ^438^ ^]^
	Co_3_O_4_/PPy|NF	Amp	2.0 × 10^−6^ m–5.0 × 10^−3^ m	0.74 × 10^−6^ m	AA, UA, Fru, H_2_O_2_	Urine	^[^ ^437^ ^]^
	TiO_2_/Co_3_O_4_|FTO	Amp	0× 10^−3^–3.0 × 10^−3^ m	0.3396 × 10^−6^ m	AA, UA	Serum	^[^ ^436^ ^]^
	Rhizobia‐like Ni–TiO_2_|GCE	Amp	1 × 10^−6^ m–7 × 10^−3^ m	0.18 × 10^−6^ m	AA, UA	–	^[^ ^435^ ^]^
HbA1c	FAO/GNPs‐PTA‐TiO_2_|ITO	CV	0.5 × 10^−6^ m–2 × 10^−3^ m	0.5 × 10^−6^ m	AA, UA, BIL, Glu, triglycerides, urea, Cys	Whole blood	^[^ ^446^ ^]^
	NanoAu–N–graphene |FTO	CV	0.3 × 10^−3^ –2 × 10^−3^ m	0.2 × 10^−6^ m	AA, BIL, urea, UA, triglycerides, Glu	Whole blood	^[^ ^441^ ^]^
	FAO/AuNPs‐PtNPs/ PIN5COOH|gold	CV	0.1 × 10^−6^ m–1 × 10^−3^ m	0.2 × 10^−6^ m	AA, UA, BIL, urea, triglycerides, Cys, Glu	Whole blood	^[^ ^442^ ^]^
	FAO/PtNPs/rGO–MWCNT|gold	CV	0.05 × 10^−6^ m–1 × 10^−3^ m	0.1 × 10^−6^ m	BIL, triglycerides, urea, AA, Cys, UA, Glu	Whole blood	^[^ ^445^ ^]^
	NanoAu–G20|SPCE	SWV	0.1–100 ng mL^−1^	0.2 ng mL^−1^	HGB, BSA	Whole blood	^[^ ^443^ ^]^
	Aptamer/TBO/pTBA@MWCNT|SPCE	Amp	0.006 × 10^−6^–0.74 × 10^−6^ m	3.7 × 10^−9^ m	AP, AA, DA, UA, HGB, Glu	Blood	^[^ ^444^ ^]^
Creatinine	TMSPMA‐GO‐*co*‐HEMA/MMA|GCE	DPV	0.5–3.0 mg dL^−1^	0.1878 mg dL^−1^	AA, AP, UA, Gly, creatine	Serum, urine	^[^ ^450^ ^]^
	CS/nafion/Sox+CA+CI/CS–COOH–SWCNT|Pt	Amp	0 × 10^−3^–0.5 × 10^−3^ m	7.8 × 10^−6^ m	AP, UA, sarcosine	Serum	^[^ ^457^ ^]^
	CD/nafion/nsPANi |Au/Al_2_O_3_	Amp	0.005 × 10^−3^ –0.4 × 10^−6^ m	1300 µA mm ^−1^ cm^−2^	NH_4_^+^	–	^[^ ^451^ ^]^
	CdSe QD‐IL|PGE	DPV	0.442 × 10^−6^ m–8.84 × 10^−3^ m	0.229 × 10^−6^ m	UA	Serum, urine	^[^ ^453^ ^]^
	Electrodeposited Cu|SPCE	LSV	6.25 × 10^−6^–378.5 × 10^−6^ m	0.0746 × 10^−6^ m	AP, DA, UA, urea, Glu	Serum	^[^ ^455^ ^]^
	Poly(ethyleneimine)/phosphotungstic acid|ITO	CV	0.125 × 10^−6^–62.5 × 10^−6^ m	0.06 × 10^−6^ m	–	Urine	^[^ ^452^ ^]^
	Cre–MA–MIP|CPE	EIS	20–670 ng mL^−1^	23 ng mL^−1^	AA, Try, UA, urea, His, DA, Epn, tyrosine, creatine	Serum, urine	^[^ ^447^ ^]^
	PMB–PVAc–Cu–CNF|ACF	DPV	0.5–900 ng mL^−1^	0.2 ng mL^−1^	DA, AA, UA, Chol, urea, Glu, glutamine, BIL	Saliva, CSF, Serum	^[^ ^456^ ^]^
	Au–SPE/PVC–COOH/MIP|SPGE	EIS DPV	0.1 ng mL^−1^–1 µg mL^−1^	0.016 ng mL^−1^ 0.081 ng mL^−1^	Urea, Glu	Urine	^[^ ^448^ ^]^
	MWCNT‐inulin‐TiO_2_ |CPE	DPV	0.2 × 10^−6^ –12 × 10^−3^ m	60 × 10^−9^ m	AA, UA, urea, Glu, K^+^, Na^+^	Urine	^[^ ^454^ ^]^
	CuNPs/PDA‐rGO‐NB|GCE	SWV	0.01 × 10^−6^–100 × 10^−6^ m	2 × 10^−9^ m	Chol, urea, creatine, AA, UA, Gly, DA, NaDH^+^, Glu	Serum, urine	^[^ ^176^ ^]^
	Ni@PANI/MIP|GCE	DPV	40 × 10^−9^–800 × 10^−9^ m	0.2 × 10^−9^ m	Tyr, UA, DA, creatine, AA	Urine	^[^ ^449^ ^]^

GA – glutamic acid; LA – lactic acid; ACFs – activated carbon microfibers; CSF – cerebral spinal fluid

### 4.5 Renal Biomarkers

#### 4.5.1 Creatinine Electrochemical Biosensors

A novice friendly and reusable carbon paste electrode was prepared using creatinine molecular imprinted polymer based on methyl acrylates monomer (Cre–MA–MIP|CPE). EIS quantification of creatinine offered a very good low detection limit of 23 ng mL^−1^ in the range of 20–670 ng mL^−1^. Selectivity of the sensor was checked in the presence of potential and biological interferents. The lab made carbon paste electrode offered a fresh electrode surface for each experiment and also a good reproducible sensor response.^[^ ^447^ ^]^

A molecularly imprinted polymer deposited on carboxylic polyvinyl chloride modified gold screen‐printed gold electrodes (PVC–COOH/MIP|Au–SPE) was demonstrated toward the selective detection of creatinine. EIS and DPV responses show a limit of detection of 0.016 and 0.081 ng mL^−1^, respectively, in the range of 0.1 ng mL^−1^–1 µg mL^−1^. Recognition matrix displayed highly selective response, simplicity of operation and low cost.^[^ ^448^ ^]^ Differential pulse voltammetric quantification of creatinine was proposed using magnetic molecularly imprinted polymer, nickel nanoparticles and polyaniline composite nanostructures deposited on GCE (Ni@PANI/MIP|GCE). A low detection limit of 0.2 × 10^−9^ m was observed in the concentration range of 40–800 × 10^−9^ m. The imprinted polymer‐based electrode displayed good selectivity and excellent reproducible sensor response toward creatinine.^[^ ^449^ ^]^

Voltammetric detection of creatinine was reported using a nanohybrid of trimethyl silane propyl methacrylate–GO copolymerized with 2‐hydroxymethacrylate/methyl methacrylate deposited on GCE [TMSPMA‐GO‐*co*‐HEMA/MMA|GCE]. The sensor performance was examined in the supporting electrolyte (pH 7.4) of 50 × 10^−3^ m NaClO_4_ and 5 × 10^−3^ m K_4_[Fe(CN)_6_]. DPV analysis of creatinine offered an LOD of 0.1878 mg dL^−1^ in the concentration range of 0.5–3.0 mg dL^−1^. The imprinted polymer‐based sensor displayed high selectivity with good reusability. The practical feasibility of the sensor was demonstrated in human blood serum and urine samples.^[^ ^450^ ^]^

Amperometric detection of creatinine was reported using a hybrid composite of creatinine deiminase enzyme, nafion, and nanostructured PANI on Au‐functionalized Al_2_O_3_ electrodes (CD/nafion/nsPANi|Au/Al_2_O_3_). Electropolymerization was used to achieve the composite film and the parameters were systematically investigated using voltammetry. Sensitivity of the sensor was calculated as 1300 µA mm ^−1^ cm^−2^ in the concentration range of 0.005 × 10^−3^–0.4 × 10^−3^ m of creatinine.^[^ ^451^ ^]^ Voltammetric quantification of creatinine was reported using indium tin oxide conductive substrate coated with poly(ethyleneimine)/phosphotungstic acid multilayer ((PEI/PTA)_20_|ITO). An electrolyte made of copper (II) ions lead to the formation of Cu(creatinine)_2_ complex. The proposed method of transduction offered a detection limit of 0.06 × 10^−6^ m creatinine in the range of 0.125 × 10^−6^–62.5 × 10^−6^ m.^[^ ^452^ ^]^

DPV sensor for the selective detection of Creatinine in the presence of uric acid has been demonstrated using quantum dots. CdSe quantum dots/ionic liquid deposited on hollow fiber‐pencil graphite electrode (CdSe/IL|PGE) were investigated. The microfabricated disposable sensor displayed a low detection limit of 0.229 × 10^−6^ m in the concentration range of 0.442 × 10^−6^ m–8.84 × 10^−3^ m creatinine.^[^ ^453^ ^]^

An enzyme‐less creatinine sensor was reported using carbon paste electrode prepared with a bionanocomposite of MWCNT, insulin, and TiO_2_ (MWCNT–insulin–TiO_2_|CPE). The resultant sensor matrix displayed a good selectivity toward creatinine. DPV analysis of creatinine offered an LOD value of 60 × 10^−9^ m in the range of 0.2 × 10^−6^ m–12 × 10^−3^ m.^[^ ^454^ ^]^

Voltammetric quantification of creatinine was reported using copper electrodeposited on screen printed carbon electrode (Cu|SPCE) which reacts with creatinine and forms copper–creatinine complex. The reported sensor displayed a low detection limit of 0.0746 × 10^−6^ m in the range of 6.25 × 10^−6^ –378.5 × 10^−6^ m.^[^ ^455^ ^]^ A carbon nanofiber electrode was decorated with the hybrid nanocomposite comprised of Cu nanoparticles, polyvinyl acetate, and polymethylene blue (PMB–PVAc–Cu–CNF|ACF). PMB was deposited on the electrode by electropolymerization. Chronoamperometry as well as DPV analysis of creatinine was carried out in the concentration range of 0.5–900 ng mL^−1^ which displayed an LOD value of 0.2 ng mL^−1^. Good selectivity was observed in the coexistence of dopamine, ascorbic acid, uric acid, cholesterol, urea, d‐glucose, l‐glutamine, and bilirubin. Practical applicability was demonstrated in the real biological body fluids namely, serum and saliva.^[^ ^456^ ^]^ An enzymeless electrochemical sensor was constructed for the detection of creatinine using a multifunctional nanohybrid of polydopamine, rGO, Nile blue, and electrodeposited copper nanoparticles on the surface of GCE (CuNPs/PDA–rGO–NB|GCE). One‐step hydrothermal reaction of graphene oxide with dopamine offered the PDA–rGO composite. NB was immobilized onto PDA–rGO with simple ultrasonication. Further, CuNPs were electrodeposited on the PDA–rGO–NB surface to achieve the desired sensing platform. The sensor performance was established based on the specific interactions of creatinine with Cu^2+^ ions on the sensor surface. SWV analysis led to the LOD of 2 × 10^−9^ m in the concentration range of 0.01 × 10^−6^–100 × 10^−6^ m creatinine. The reported enzymeless sensor matrix displayed high selectivity toward creatinine in the presence of potential interferents.^[^ ^176^ ^]^

A facile and robust recognition matrix was reported for the electrochemical detection of creatinine and sarcosine using chitosan, SWCNT, MWCNT, sarcosine oxidase, creatinase, and creatininase enzymes and nafion immobilized on the electrode surface of Pt (CS/nafion/SOX+CA+CI/CS–COOH–SWCNT|Pt). Amperometric analysis of creatinine offered the sensitivity 0.57 µA mm ^−1^ and LOD of 7.8 × 10^−6^ m in the range of 0.5 × 10^−3^ m. Capping of the recognition matrix with additional layer of chitosan offered high sensitivity toward the detection of both the analytes sarcosine and creatinine.^[^ ^457^ ^]^

Recognition matrices composed of metallic nanoparticles and/or mixed metallic derivatives yielded much better current sensitivity and thus superior low‐detection‐limits of selected vital biomedical markers. Even though the presence of aptamers, antibodies, etc., facilitated the selective determination of such biomarkers, the enhanced current sensitivities were achieved when these mixed metallic derivatives were utilized irrespective of the signal transduction methods, CA, DPV, SWV, and EIS. From these observations, it can be concluded that the signal transduction methodology could be chosen freely between these methods—CA, DPV, SWV, and EIS considering the practical requirements of specific applications, time of analyses, cost effectiveness, sample nature, ease‐to‐use methodology, and instrumentation.

## 5 Challenges in Electrochemical Sensors for Vital Biomarkers

The key challenges involved in the electrochemical detection of biomarkers are i) extremely difficult direct analysis of trace levels of biomarkers from complex physiological matrices with high specificity and sensitivity,^[^ ^211^ ^]^ ii) biofouling of the sensor interface due to the nonspecific adsorption of proteins in physiological body fluids,^[^ ^155^, ^458^ ^]^ iii) biocompatibility of the recognition matrix,^[^ ^459^ ^]^ iv) reproducibility of the sensor performance,^[^ ^207^ ^]^ v) reusability or storage stability of the sensor for long time,^[^ ^368^ ^]^ vi) continuous monitoring of biomarkers,^[^ ^108^ ^]^ vii) measurement of multiple biomarkers utilizing a little single serum sample,^[^ ^226^ ^]^ viii) construction of portable, noninvasive, wearable, and point‐of‐care oriented devices,^[^ ^460^ ^]^ ix) difficulty in drawing conclusions from the results of biomarker analysis as many biomarkers are correlated to more than one form of cancer or disease conditions,^[^ ^226^ ^]^ x) wide changes in background protein concentrations of serum with individuals, age and/or disease conditions, xi) simultaneous measurement of some biomarkers, which present at low concentrations (<pg mL^−1^) and the others at high concentrations (>ng mL^−1^).^[^ ^226^ ^]^ Furthermore, other challenges involved in the development of modified electrodes with nanostructured materials and composites as catalytic interfaces are i) heterogeneity at the nanoscale level, ii) instability of shape, size, and surface modification of nanomaterials, iii) gradual change/deterioration of the catalytic activity/sensitivity, iv) unoptimized/nonreproducible synthetic procedures of immunosensors,^[^ ^461^ ^]^ and v) rare but random fouling of disposable sensor chips involving detrimental false positive or negative responses.

One of the diagnostic challenges is the accurate and selective measurement of vital biomarkers from complex physiological body fluids such as blood, urine, and tissue samples. To overcome this challenge, protein contents of the biological samples were removed by the centrifugal filtration.^[^ ^204^ ^]^ Modified sensor interfaces with multiple layers of enzymes, antibodies, catalytic materials, etc. help to overcome both the complexity of the sample matrices and the trace levels of biomarkers. Magnetic nanoparticle‐based recognition systems facilitated the magnetic separation of target analytes from the physiological body fluids by using external magnet.^[^ ^284^ ^]^

Coagulation, hemolysis ratio, and whole blood adhesion tests were applied to examine the biocompatibility and antibiofouling property of the recognition matrix.^[^ ^324^, ^405^, ^458^, ^462^, ^463^, ^464^ ^]^

Reproducibility of the sensor performance was demonstrated with different number (>5) of electrodes^[^ ^207^, ^317^, ^330^, ^351^, ^406^, ^411^ ^]^ prepared under the same experimental conditions. Reusability^[^ ^354^ ^]^ or storage stability^[^ ^206^ ^]^ of the sensor was examined with the same electrode at different time intervals—weeks^[^ ^423^, ^442^, ^445^ ^]^ or months^[^ ^431^, ^441^, ^446^ ^]^ to validate the long term usage of the constructed sensor system.

Consequently, the continuous monitoring of various biomarkers in patients is an important criterion especially for long‐term diseases such as diabetes, Parkinson's disease, etc. In such cases, continuous evaluation of biomarkers is a great asset to monitor and alert the actual condition of patient, to initiate timely medical treatment (clinical processes), to stir the future course of medical treatment, and to predict the future course of patient's progress and recovery. Minimal invasive wearable electrochemical sensors were proved to play very important role in the continuous monitoring of glucose and lactose levels in body fluids. Further development in wearable patch sensors,^[^ ^465^ ^]^ microneedle sensors,^[^ ^466^ ^]^ and textile sensors can fulfill such needs. Here, biocompatible materials like chitosan, lignin, sodium oleate, and cellulose‐based materials play major role.^[^ ^285^, ^335^ ^]^ Furthermore, coupling these noninvasive electrochemical biosensors with microfluidic devices is expected to enhance further on‐body measurements of the dynamically changing biomarker signals.

Different sensor systems comprising hybrid nanostructures developed for the simultaneous detection of multiple biomarkers in serum samples^[^ ^295^, ^411^, ^415^, ^467^, ^468^, ^469^, ^470^ ^]^ were successfully demonstrated, which proved the possibility of simultaneous detection of multiple biomarkers.

Miniaturized electrochemical sensing through portable instrumentation for point of care analysis is emerging toward “lab on chip to point of care analysis to wearable devices.” There is a good correlation of biomarker levels between blood and other biofluids (interstitial fluid, sweat, tears, and saliva^[^ ^471^ ^]^). Hence, the monitoring of trace levels in other biofluids with minimal invasion would facilitate frequent timely analyses, continuous monitoring, real‐time backup during surgical or intensive‐care treatments. This will help to realize comprehensive patient's health care steadily in fast and reliable manner. With the use of wearable sensing technologies (glove, mouthguard, ring, spectacles, microneedle, textile, tattoo sensors, etc.), the challenges in the analysis of biomarkers would be overcome and thus reliable, timely, and comprehensive medical diagnosis and treatment would be achieved.

Further advancements in the preparation of hybrid nanostructured materials with rational approach are expected to offer the most efficient recognition matrix toward the selective detection of crucial biomarkers.

## 6 Summary, Conclusions, and Perspectives

In this review, we have collectively summarized the recent developments in the selective detection of cancer (Prostate‐specific antigen, Carcinoembryonic antigen, *α*‐fetoprotein, neuron‐specific enolase, Ferritin), cardiac (troponin I, myoglobin, superoxide anion, myeloperoxidase, thrombin), inflammatory (nitric oxide, tumor necrosis factor ‐*α*, C‐reactive protein, interleukin‐6), diabetic (glucose, glycated hemoglobin), and renal (creatinine) biomarkers. Numerous approaches used in the synthesis of advanced hybrid metallic composite nanostructures have been reviewed meticulously. Sensor systems which involved electrochemical (Amp, CV, DPV, SWV, EIS) as well as other transduction methods (fluorescence, CL, ECL, electrophoresis, SPR, SERS) have been summarized scrupulously to present an overview of the current research works. Various challenges involved in the construction of electrochemical sensor systems have been listed. Strategies resulting to enhanced sensitivity, high selectivity, reduced analytical times, and reusability have been proposed.

It was observed that the mixed metallic nanoparticles and/or mixed metallic derivatives (metallic oxides/sulfides/selenides/tellurides) along with functionalized carbon nanomaterials yielded much better current sensitivity toward the detection of selected vital biomarkers. Synergism between the catalytic activity of mixed metallic derivatives and the improved conductivity of nanocarbon derivatives has allowed to achieve ultralow‐detection‐limits even up to femtomolar levels. Furthermore, the hybrid nanostructured materials facilitated the homogeneous and uniform immobilization of biorecognition elements which helped to achieve enhanced selectivity by incorporating the suitable antibody/aptamer. Thus, the researchers used numerous combinations of hybrid nanostructured materials as the recognition matrices to get such properties. But, the selection of nanocomposite materials was random akin to changing the dopant material or metal or metallic derivatives. This type of selection is inappropriate and there should be a certain logical approach behind the selection of different hybrid nanomaterials. The selection criteria must be concerned about the properties of biorecognition elements such as hydrophilicity, dielectric constants, zeta potentials, and most importantly loading. In addition, biocompatibility of the hybrid nanomaterials is an important parameter which would be helpful in constructing on‐body wearable electrochemical sensing devices. Usage of the environment hazardous materials must also be reduced during the synthesis of materials. These considerations would be helpful in the selection of a hybrid nanomaterial toward the fabrication of successful electrochemical biosensor systems.

Superior low detection limits of biomarkers have been reported with the hybrid nanostructures irrespective of the signal transduction methods, CA, DPV, SWV, and EIS. From these observations, it can be concluded that the signal transduction methodology could be chosen freely between these electrochemical techniques—CA, DPV, SWV, and EIS considering the practical requirements of specific applications, time of analyses, cost effectiveness, sample nature, ease‐to‐use methodology, and instrumentation. Hence any of these techniques can be used in the construction of point of care devices or portable sensor systems.

Monitoring of salivary biomarkers has become the latest trend in molecular diagnostics at the biomedical, basic, and clinical research level.^[^ ^411^, ^413^, ^456^, ^471^ ^]^ Recent inventions in the saliva collection devices have made the collection of clinical samples not just effortless but also secure and noninvasive. Further extensive research in the salivary diagnostics will definitely provide complementary results to those from serum samples, which help to draw the flawless conclusions from the analysis of biomarkers. In addition to that, advancements in the simultaneous detection of multiple (four or more) biomarkers in clinical samples is expected to improve the molecular diagnostics.

Integration of biosensors to smart phones or point of care devices can facilitate the construction of portable and user‐friendly analytical devices. Portable sensors can simplify the medical procedures including early detection, diagnostics, and treatment of severe diseases. Users can perform quick, robust, and easy bioanalysis at any time. This can boost the medical and public health service delivery in low‐resource settings, and improve access to medical services globally. Despite the large number of research reports in the field of biosensors, there are no much portable products available in the market apart from glucose sensors. There is a desperate need of such sensor systems for the selective quantification of biomarkers to monitor severe diseases. We hope the advanced hybrid composite nanostructures would play pivotal role in the construction of such portable sensor systems.

Recent reports in the detection of biomarkers using point of care devices^[^ ^126^, ^292^, ^472^ ^]^ and smart phones^[^ ^329^, ^473^ ^]^ with the aid of hybrid composite nanostructures is expected to facilitate the construction of such portable sensor systems not only for the biomarkers but also for the other target analytes in food quality control and environmental applications. With the promising nature of the electrochemical sensors developed for biomarkers with high sensitivity, selectivity, trace analysis, low‐sample volumes, direct analysis in real samples and miniature and portable instrumentation, research and development for large‐scale industrial and commercial biosensor chips would necessarily lead to promising commercial smart sensing devices. Innovative approaches demonstrated for the detection of selected vital biomarkers indicate that the electrochemical biosensors would become promising devices that fill existing technology gaps in the near future. The next generation sensor systems will be governed by the forthcoming advancements in wearable sensing technologies (glove, mouthguard, ring, spectacles, microneedle, textile, tattoo sensors, etc.). Collaborative works between the researchers working in the areas of electrochemical sensors, materials chemistry and biotechnology will lead to construct such electrochemical sensor systems successfully.

## Conflict of Interest

The authors declare no conflict of interest.
